# 4‐Iodopyrimidine Labeling Reveals Nuclear Translocation and Nuclease Activity for Both MIF and MIF2**

**DOI:** 10.1002/chem.202103030

**Published:** 2021-11-22

**Authors:** Zhangping Xiao, Deng Chen, Fabian Mulder, Shanshan Song, Petra E. van der Wouden, Robbert H. Cool, Barbro N. Melgert, Gerrit J. Poelarends, Frank J. Dekker

**Affiliations:** ^1^ Department Chemical and Pharmaceutical Biology Groningen Research Institute of Pharmacy (GRIP) University of Groningen Antonius Deusinglaan 1 9713 AV Groningen The Netherlands; ^2^ Molecular Pharmacology Groningen Research Institute of Pharmacy (GRIP) University of Groningen Antonius Deusinglaan 1 9713 AV Groningen The Netherlands; ^3^ University Medical Center Groningen Groningen Research Institute of Asthma and COPD University of Groningen Hanzeplein 1 9713 GZ Groningen The Netherlands

**Keywords:** 4-Iodopyrimidine, D-dopachrome tautomerase, DNA cleavage, fluorescent probe, macrophage migration inhibitory factor

## Abstract

Macrophage migration inhibitory factor (MIF) and its homolog MIF2 (also known as D‐dopachrome tautomerase or DDT) play key roles in cell growth and immune responses. MIF and MIF2 expression is dysregulated in cancers and neurodegenerative diseases. Accurate and convenient detection of MIF and MIF2 will facilitate research on their roles in cancer and other diseases. Herein, we report the development and application of a 4‐iodopyrimidine based probe **8** for the selective labeling of MIF and MIF2. Probe **8** incorporates a fluorophore that allows in situ imaging of these two proteins. This enabled visualization of the translocation of MIF2 from the cytoplasm to the nucleus upon methylnitronitrosoguanidine stimulation of HeLa cells. This observation, combined with literature on nuclease activity for MIF, enabled the identification of nuclease activity for MIF2 on human genomic DNA.

## Graphical abstract

**Protein trafficking** is an essential step for the functionalization of proteins. We report a 4‐iodopyrimidine based probe **8** for the selective labeling of MIF and MIF2. This enabled monitoring of the translocation of MIF2 from the cytoplasm to the nucleus upon methylnitronitrosoguanidine stimulation, which indicates a nuclease activity for MIF2 on human genomic DNA. Thus, we provide a tool for MIF and MIF2 localization study, as well as demonstrate a nuclease activity for MIF2.

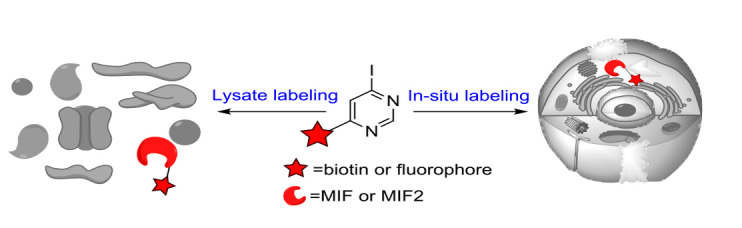

Macrophage migration inhibitory factor (MIF) is a multifaceted protein that plays key roles in cell growth and immune responses.^[1]^ The dysregulation of MIF expression has been implicated in many diseases, including cancers and inflammatory diseases.^[2]^ Several MIF‐targeted therapeutics provided beneficial effects for cancer treatment in models ranging from cell‐based studies, up to animal models, and even clinical trials.^[3]^ Importantly, progression in the development of chemical tools enables advances in MIF‐oriented research.^[4]^ D‐dopachrome tautomerase (DDT or MIF2) is a structural and functional homolog of MIF with both overlapping and distinct properties and functions. MIF and MIF2 share 34 % sequence identity and have almost identical 3D structures as homotrimers.^[5]^ Moreover, MIF2 interacts with several binding partners of MIF such as CD74 and JAB1, which may suggest MIF2 has functional redundancy to MIF.^[6]^ Our team has shown that MIF2 is involved in lung epithelial cell proliferation and other researchers recognized MIF2 as a potential target for cancer therapeutics.^[7]^ Further investigation of the functions of MIF‐family proteins would benefit from convenient tools to visualize their subcellular localization.

The covalent modification of proteins by small molecules has widespread applications in drug design, activity‐based protein profiling and protein labeling.^[8]^ The effort to develop a covalent inhibitor or probe of a protein typically involves identifying a potent noncovalent inhibitor, to which a tempered electrophile, such as an acrylamide or chloroacetamide, is installed to facilitate covalent bond formation with proximal nucleophilic amino acid residues.^[9]^ Most covalent inhibitors react with lysine and cysteine amino acid residues.^[9]^ However, these modifiers cause labeling at multiple sites, as lysine and cysteine are highly abundant on the protein surface.^[10]^ Proline is a unique amino acid which provides a reactive secondary amine only if it exists as a N‐terminal residue. This property endows an N‐terminal proline with the potential for selective bioconjugation.^[11]^ However, it remains difficult to exploit the reactivity of the N‐terminal proline in living cells, because of the need to use a reagent with high reactivity, multiple reaction components, or harsh conditions. A group of promising scaffolds that react specifically with N‐terminal proline residues under mild conditions are electrophilic aromatic fragments, whose reactivity can be predictably tuned by variation of electron withdrawing or donating groups, or by changing the leaving groups.^[12]^ The potential of aromatic electrophiles for biorthogonal labeling of an N‐terminal proline has been demonstrated by Waldmann et al. who reported Woodwards reagent K‐derived activity‐based probes that label the proline‐1 residue of MIF remarkably selective in living cells_._ ^[4]^

Electron‐deficient halogenated (hetero)aryls have been employed for covalent labeling of nucleophilic cysteine or lysine residues in cellular proteins. The nucleophilic aromatic substitution reaction required for labeling can be tuned by the aromatic substitution pattern.^[13]^ In this perspective, the irreversible MIF and MIF2 inhibitor 4‐iodo‐6‐phenylpyrimidine (4‐IPP) holds promise for development of probes for covalent modification of the nucleophilic N‐terminal proline residue.^[14]^ 4‐IPP proved to be a promising inhibitor since treatment with 4‐IPP exhibited inhibition of cancer growth and osteoclast formation in cell‐based studies and animal models.^[15]^ Nevertheless, target engagement of 4‐IPP in the proteome remains unknown.

In this study, we aimed to employ 4‐iodopyrimidine inhibitors to explore 4‐IPP target engagement in a cellular environment. We firstly developed potent inhibitors that were capable of highly selective labeling of MIF and/or MIF2 in cell lysates. Subsequently, these optimized 4‐iodopyrimidines were exploited as probes to visualize the subcellular localizations of MIF and/or MIF2, which provided important clues about their functions. Thus, these probes expand the toolbox to unravel the diverse biological roles of MIF and MIF2, which opens up new opportunities to exploit these protein targets in drug discovery.

We explored the structure‐activity relationships of 4‐IPP derivatives for inhibition of MIF family proteins in order to estimate their potency and selectivity against MIF and MIF2. Moreover, we aimed to identify a point of attachment for biotin or a fluorophore that provides minimal interference with binding. Towards this aim, a focused compound collection was synthesized through a two‐step synthetic route. Firstly, a Suzuki reaction was employed to couple 4,6‐dichloropyrimidines with various phenylboronic acids to prepare chloro‐substituted intermediates.^[16]^ Subsequently, the intermediates were iodinated by HI to afford 4‐IPP (**1**) and its analogs (**2**–**7**).^[17]^ Compounds **1**–**7** were tested for inhibition of MIF and MIF2 tautomerase activity using assay conditions as described previously.^[4]^ The IC_50_ values are shown in Table 1. For 4‐IPP, an IC_50_ of 4.0±0.98 μM was observed for MIF and >250 μM for MIF2, respectively, which is in line with its potency for MIF and MIF2 inhibition in literature.^[18]^ Carboxylic acid substitution of the phenyl *para*‐position provides inhibitor **2**, which preserved MIF inhibition and enhanced MIF2 inhibition. In contrast, methyl (**3**), morpholinyl (**4**), or phenyl (**5**) substitution at the pyrimidine 2‐position or methyl (**6**) substitution at 4‐position of pyrimidine decrease the inhibitory potency on both MIF and MIF2. Changing the carboxyl group of **2** to a nitro group (**7**) results in a significant increase of MIF inhibition, while **7** loses potency on MIF2, thus providing **7** as a discriminating inhibitor that shows >400‐fold improved selectivity for MIF over MIF2. In addition, we found that compounds **1**, **2**, **4**, **6** and **7** are at least two times more potent than their corresponding chloro‐substituted intermediates (Table S1). Taken together, the SARs demonstrate that the iodo‐substituted pyrimidine is critical for binding to MIF and MIF2, whereas substitutions on the phenyl group are well‐tolerated.

**Table 1:** Affinity data for binding of 4‐IPP and its derivatives for inhibition of MIF and MIF2 tautomerase activity. Data represent mean**±**SD (n=3).

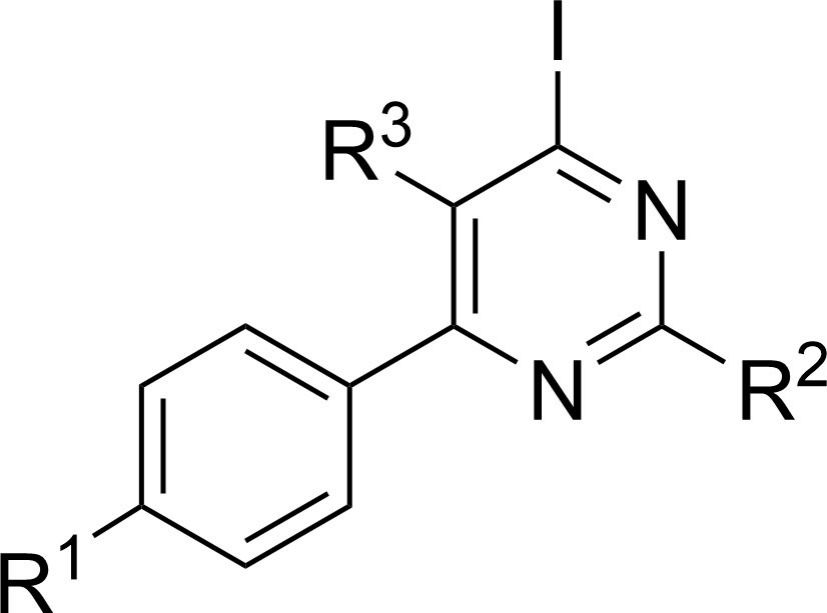					
**Cpd**.	R^1^	R^2^	R^3^	IC_50_ [μM]	
				MIF	MIF2
4‐IPP **(1)**	H	H	H	4.0±0.98	>250
**2**	COOH	H	H	5.0±0.46	125±15
**3**	COOH	CH_3_	H	>250	>250
**4**	COOH	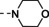	H	15±7.5	>250
**5**	COOH	Ph	H	59±6.3	>250
**6**	COOH	H	CH_3_	56±12	>250
**7**	NO_2_	H	H	0.63±0.06	>250

Our new insights on structure‐activity relationships (SARs) of MIF and MIF2 binding of 4‐iodopyrimidines enabled design of chemical probes **8** and **9**. Probe **8** contains a nitrobenzofurazan (NBD) fluorophore to enable visualization by fluorescence spectroscopy and probe **9** contains a biotin tag for (strept)avidin binding to enable detection and enrichment (Figure 1). Interestingly, both **8** and **9** showed improved potency for inhibition of MIF and MIF2 tautomerase activity compared to parental inhibitor **2** (Table 2). The IC_50_ values of **8** and **9** on MIF improved by a factor 20 and went down to values around 200 nanomolar. Additionally, for MIF2 inhibition the potencies of **8** and **9** were improved by a factor 2–6 to provide IC_50_ values of 20±2.2 and 78±7.7 μM, respectively. We further characterized inhibition kinetics for probes **8** and **9** using Kitz‐Wilson analysis to distinguish the equilibrium binding constant (*K*_I_) from the rate of covalent inactivation (*k*_inact_). Probes **8** and **9** show high maximum potential rate on MIF labeling with *k*_inact_ values of 0.20 and 0.24 min^−1^, respectively. The *K*_I_ values of these two probes on MIF are both at a low micromolar level, which is about 10 times higher than the IC_50_ values. For MIF2, **8** and **9** exhibit *k*_inact_ values of 0.06–0.09 min^−1^ and *K*_I_ values around their IC_50_ values. Taken together, probes **8** and **9** are able to bind covalently with both MIF and MIF2, however the binding kinetics are clearly distinct.

**Figure 1:**
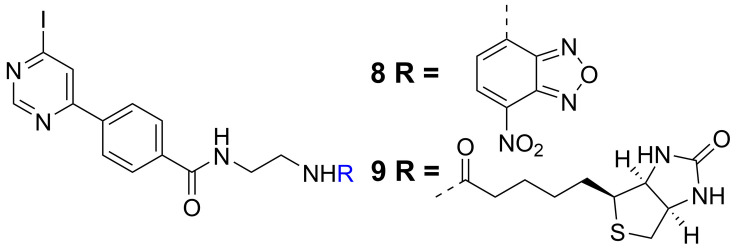
Chemical structures of probe **8** and probe **9**.

**Table 2:** Characterization of probes on MIF and MIF2 tautomerase inhibition. IC_50_ values with 10 min pre‐incubation, inhibition constant (*K*_I_ *)* and rate of enzyme inactivation (*k*_inact_). All values are reported with the standard deviation. *n*=3.

	**7**		**8**		**9**	
	MIF	MIF2	MIF	MIF2	MIF	MIF2
IC_50_ (μM)	0.63±0.06	>250	0.27±0.085	20±2.2	0.24±0.050	78±7.7
K_I_ (μM)	3.9±0.70	–	1.5±0.20	26±3.2	2.8±0.67	85±8.6
k_inact_ (min^−1^)	0.28±0.04	–	0.20±0.03	0.088±0.012	0.24±0.06	0.061±0.010

As a next step, inhibitor **8** was employed to label recombinant human MIF and MIF2 for subsequent fluorescence detection. Firstly, we investigated the concentration‐dependence of MIF labeling upon 10 min pre‐incubation with different concentrations of **8** using SDS‐PAGE separation and fluorescence detection. We observed a clear fluorescent band upon incubation with 1 or 10 μM of **8** (Figure 2A). Subsequently, the time‐dependence of MIF labeling by **8** was investigated by use of 5 μM **8**. Fluorescent bands appeared after one‐minute pre‐incubation and continued to increase in intensity until 15 min of pre‐incubation (Figure 2B), which indicates that MIF can be labeled in time window in the order of min, which is in line with the *t*_1/2_ of 4 min calculated from the *k*_inact_ of 0.38 min^−1^ and *K*_I_ of 1.5 μM for MIF binding of **8**. As a control experiment the labeling of MIF by **8** could be abolished upon prior treatment of MIF with the covalent MIF tautomerase inhibitor **2** or the non‐covalent MIF tautomerase inhibitor **10** (Figure 2C, D, for chemical structure **10** see supporting information).^[4]^ These observations indicate that probe **8** is capable of labeling MIF effectively in a concentration‐ and time‐dependent manner and that MIF labeling is competitive with inhibitors that are known to bind to the MIF tautomerase active site.^[14]^

**Figure 2:**
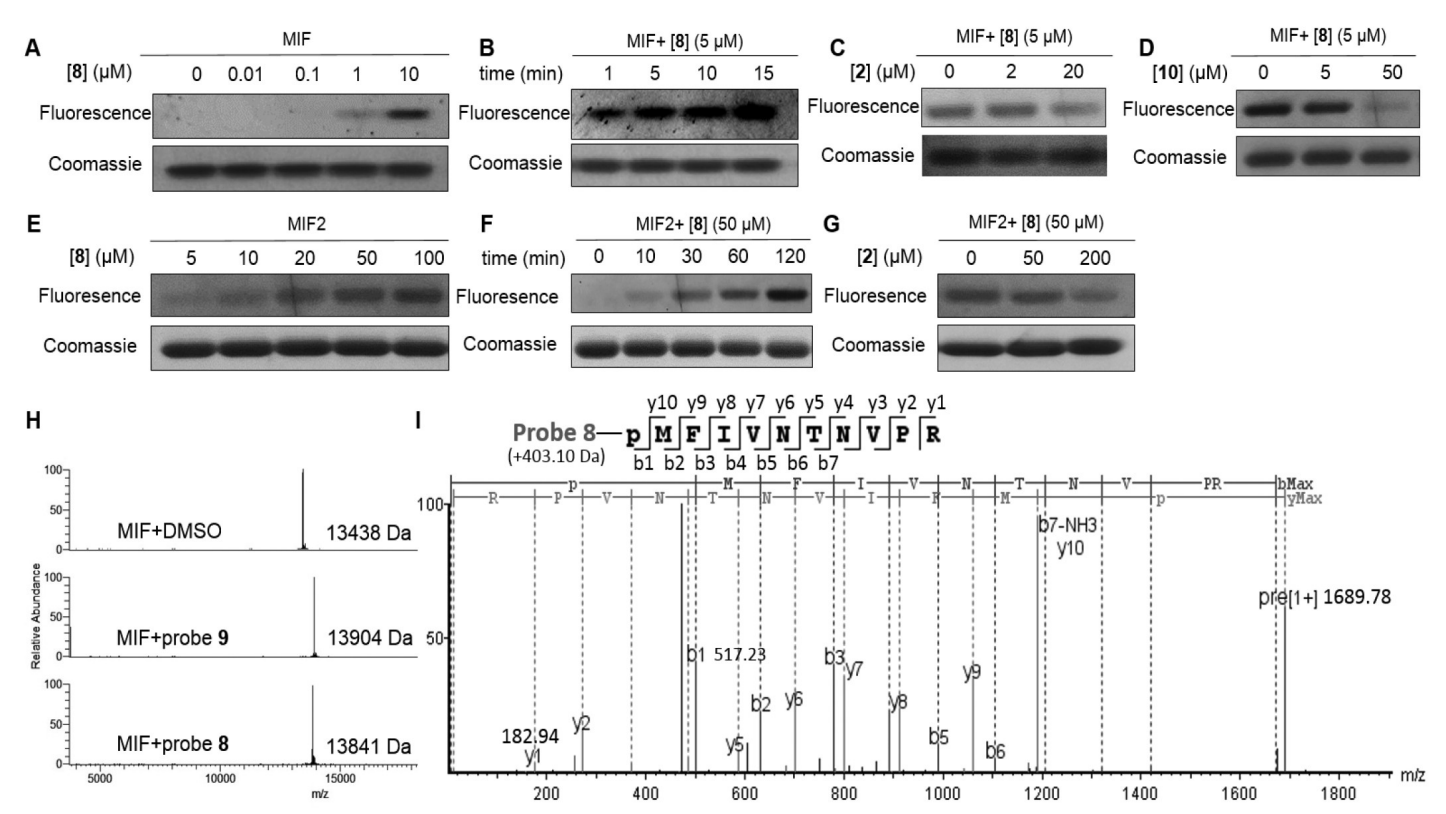
Labeling MIF and MIF2 by 4‐iodopyrimidine based probe ***8*** in vitro. Probe **8** labels MIF in A) concentration‐ and B) and time‐dependent manner. The labeling of MIF by probe **8** is competitive with C) inhibitor **2** and D) reversible MIF tautomerase inhibitor **10**. Probe **8** also reacts with MIF2 in E) concentration‐ and F) time‐dependent manner. G) The labeling of MIF2 by probe **8** is competitive with **2**. H) MIF monomer forms complex with probe **8** or **9** in a ratio of 1 : 1 detected by mass spectra. I) Pro‐1 is the only residue that reacts with probe **8**. *n*=2.

A similar set of experiments was done for MIF2. MIF2 labeling appeared after 10 min pre‐incubation with 5–10 μM **8** and gained intensity up to 100 μM. Subsequently, time dependence was investigated for a concentration of 50 μM and we observed a clear gain in intensity up to 120 min pre‐incubation (Figure 2E, F). MIF2 labeling with **8** could be outcompeted upon pre‐incubation with inhibitor **2** (Figure 2G). Altogether, this demonstrates that **8** covalently binds to MIF and MIF2 in a time‐ and concentration‐dependent way and that it binds in competition with known inhibitors that bind to the tautomerase active site. Importantly, probe **8** exhibits differential kinetics for binding to MIF and MIF2. We aim to utilize these differences in kinetics to distinctly label MIF and MIF2.

Mass spectrometry was applied to verify if 4‐iodopyrimidine labeling of MIF and MIF2 occurs at the proline‐1 (Figure 2H, I, S2, S3). Mass‐spectrometric analysis of intact MIF and MIF2 before and after the reaction with **8** showed mass shifts that correspond to the desired products, indicating covalent modification of the MIF or MIF2 monomer in a 1 : 1 ratio. The labeling site was determined by LC–MS/MS analysis after digestion with trypsin, demonstrating that proline‐1 is the only site modified by **8** for both MIF and MIF2. These results thus indicate that the catalytically active proline‐1 of MIF and MIF2 is the reactive site for labeling with 4‐iodopyrimidines.^[14]^

After concluding that 4‐iodopyrimidine **8** efficiently labeled both MIF and MIF2 in vitro, we aimed to interrogate the specificity of the 4‐iodopyrimidine warhead among cellular proteins. Towards this aim, a lysate of A549 cells was incubated with biotin‐tagged probe **9** and the resulting proteome labeling was analyzed using western‐blot. A major band was detected around 15 kDa by chemiluminescent detection using HRP‐conjugated streptavidin. The intensity of this band depended on both the concentration of the probe and the incubation time (Figure 3A, B). Notably, the position of these bands correspond to the size of MIF and MIF2, which both have a molecular weight of around 13 kDa. The biotin tag of **9** was utilized for enrichment experiments to confirm MIF and MIF2 labeling. Cellular proteomes were labeled with **9**, enriched using streptavidin‐coated beads and analyzed by western‐blot (Figure 3C). MIF and MIF2 are detected in both HeLa and A549 cell lysate by specific antibodies (Figure 3D, E). These results demonstrate that **9** is able to label both MIF and MIF2 in cell lysate samples.

**Figure 3:**
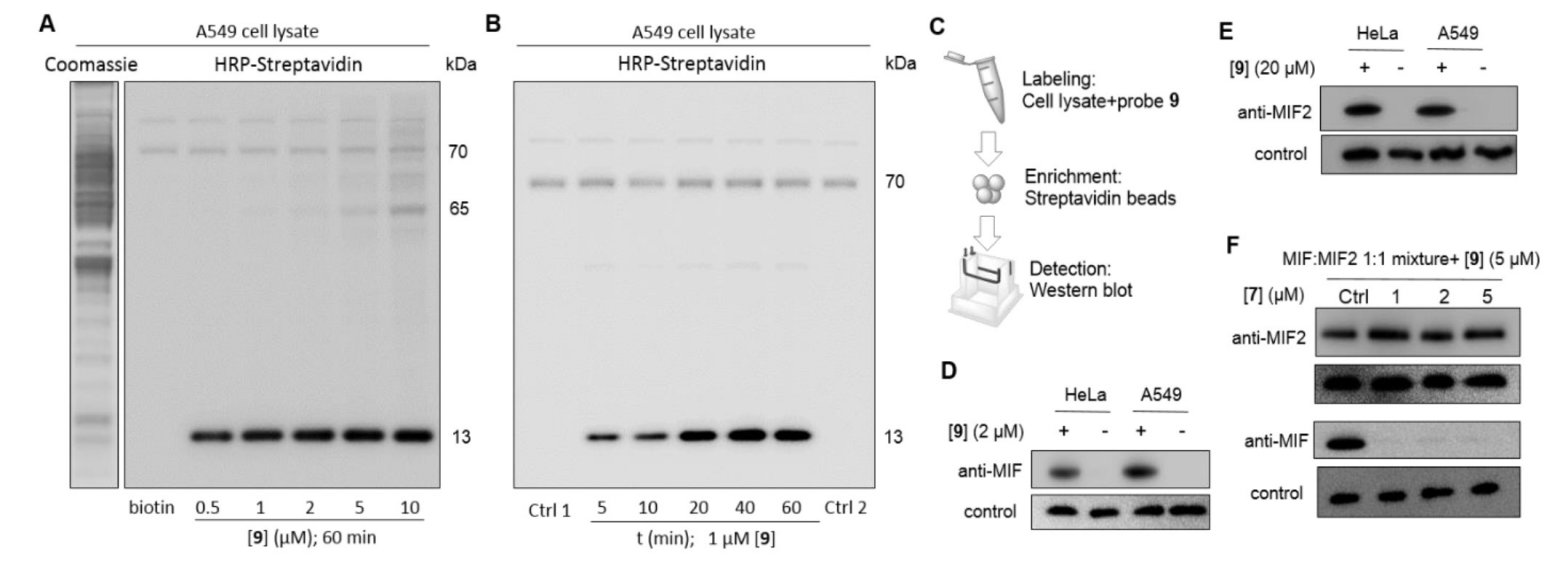
Labeling of the cellular proteome using probe **9**. A) Concentration‐ and B) time‐dependent labeling of A549 cell lysate with **9**. Biotin was employed as control. C) Schematic representation of the procedure for enrichment and analysis of proteins labeled by **9**. D, E) Analysis of probe **9**‐labeled proteins in HeLa or A549 cell lysates using anti‐MIF or anti‐MIF2 antibodies. F) Analysis of a MIF:MIF2 mixture (1 : 1) in which MIF was blocked with various concentrations of **7** followed by labeling with **9**. *n*=2.

Next, we explored the possibility to preferentially label MIF2 using probe **9** after blocking MIF using the MIF selective inhibitor **7**. Towards this aim, a 1 : 1 mixture of MIF and MIF2 was employed. We observed that probe **9** was able to preferentially label MIF2 in the mixture after pre‐incubation with **7** (Figure 3F). This result demonstrates that MIF2‐directed labeling in the presence of MIF is feasible using inhibitor **7** as a selective MIF blocker in combination with probe **9**.

Fluorescent probe **8** provided efficient and specific labeling of MIF and MIF2, which provides opportunities to employ **8** to study the localization of MIF and MIF2 in living cells. HeLa cells were treated with **8** (10 μM, 2 h), which is a concentration that enables effective labeling of MIF but a limited labeling of MIF2 in our studies on purified proteins. Fluorescence imaging shows bright fluorescence (*λ*_ex_=488 nm) that distributes evenly in the cytoplasm, while a weak signal was found in the nucleus. More importantly, **8** is clearly co‐localized with the MIF signal visualized by incubation with MIF antibody and a fluorescent second antibody (*λ*_ex_=555 nm). These results indicate that **8** is capable of labeling MIF effectively in living cells. To gain selectivity for MIF2, we utilized the MIF selective inhibitor **7** (10 μM, 0.5 h) as a blocker for MIF followed by treatment with **8** (50 μM, 3 h). Under these conditions, **8** appears mainly in the cytoplasm and less in the nucleus, which is similar to MIF‐directed labeling. However, MIF2‐directed labeling shows a clear accumulation of **8** around the nuclear membrane, which is different from the distribution of signal for the MIF‐directed labeling, indicating that MIF2 could function differently from MIF. Importantly, the fluorescence signal for MIF2‐directed labeling overlapped well with the signal observed by staining with an anti‐MIF2 antibody. Application of competitive inhibitor for the MIF or the MIF2 tautomerase active site in combination with probe **8** provide a clear reduction of the fluorescence observed as well as a disappearance of the characteristic subcellular distribution of either MIF or MIF2 (Figure S6). Taken together, we conclude that probe **8** can be utilized for MIF‐ or MIF2‐directed labeling to provide labeling patterns that overlap very well with antibody‐based MIF or MIF2 staining.

Intracellular localization of MIF and MIF family proteins gained importance in the context of the recently discovered role of MIF as a nuclease. In 2016, Wang et al. identified MIF as a nuclease, which is recruited by apoptosis‐inducing factor (AIF) to the nucleus to cleave genomic DNA (hgDNA) and to cause cell death in response to oxidative stress or DNA damage.^[18]^ In this perspective, we aimed to employ probe **8** to monitor nuclear translocation of MIF and MIF2. HeLa cells were pre‐treated with 50 μM methylnitronitrosoguanidine (MNNG) for 15 min followed by the addition of probe **8** for MIF‐directed staining or inhibitor **7** and probe **8** for MIF2‐directed staining. Also, for MNNG treatment, the probe **8** labeled MIF and MIF2 distribution pattern colocalizes with the respective antibodies. MIF‐directed labeling using probe **8** demonstrated a substantial nuclear translocation of MIF upon treatment with MNNG. This phenomenon is in line with the study of Wang et al.^[18]^ Notably, signals of MIF2‐directed labeling using probe **8** also accumulated in the nucleus upon treatment with MNNG (Figure 4). This observation raises the idea that MIF2 might also play a role as nuclease similar to that described for MIF.^[18]^

**Figure 4:**
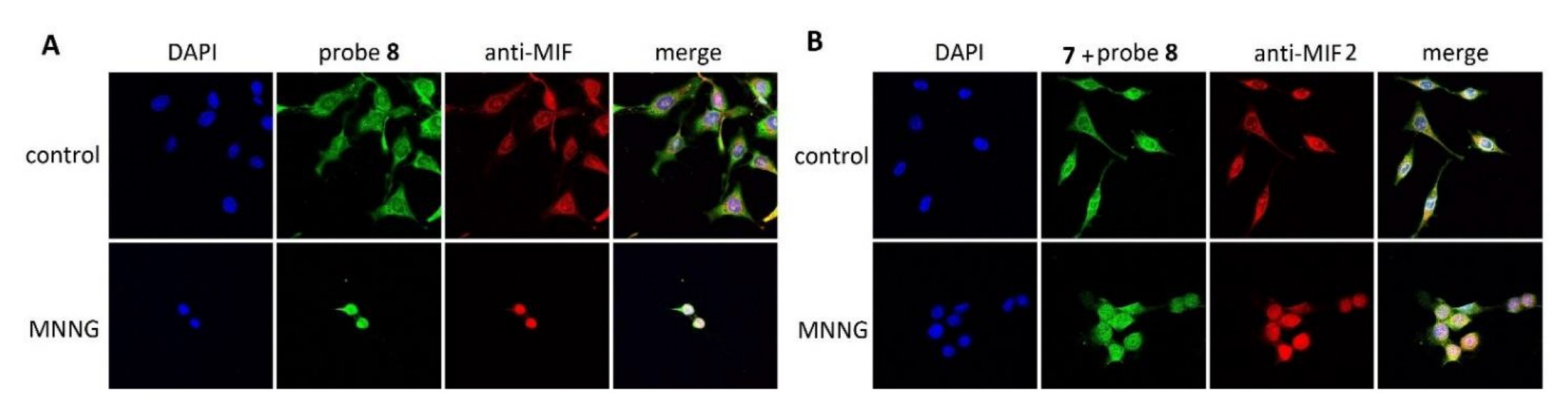
Histochemical (probe **8**) and immunohistochemical staining of HeLa cells upon stimulation with MNNG. A) MIF‐directed labeling using probe **8** or anti‐MIF antibodies in HeLa cells that were stimulated with MNNG compared to vehicle control. B) MIF2‐directed labeling of HeLa cells preincubated with **7**, stimulated with MNNG or vehicle control, and subsequent labeling with **8**.

Accordingly, we investigated whether MIF2 can act as a nuclease. Sequence analysis showed that MIF2 contains a PA‐EK motif at a similar position as the PD‐EK motif found in MIF. The PD‐EK motif is a feature that is found in many nucleases, including MIF. For MIF it was found that a D to A mutation in this motif retains MIF nuclease activity.^[18]^ Interestingly, the PA‐EK motif of MIF2 is highly conserved across mammalian species (Figure 5A). Taken together, sequence analysis indicates that MIF2 contains a sequence similar to the PD‐D/E(X)K motif, which is characteristic for a group of proteins containing nuclease activity.

**Figure 5:**
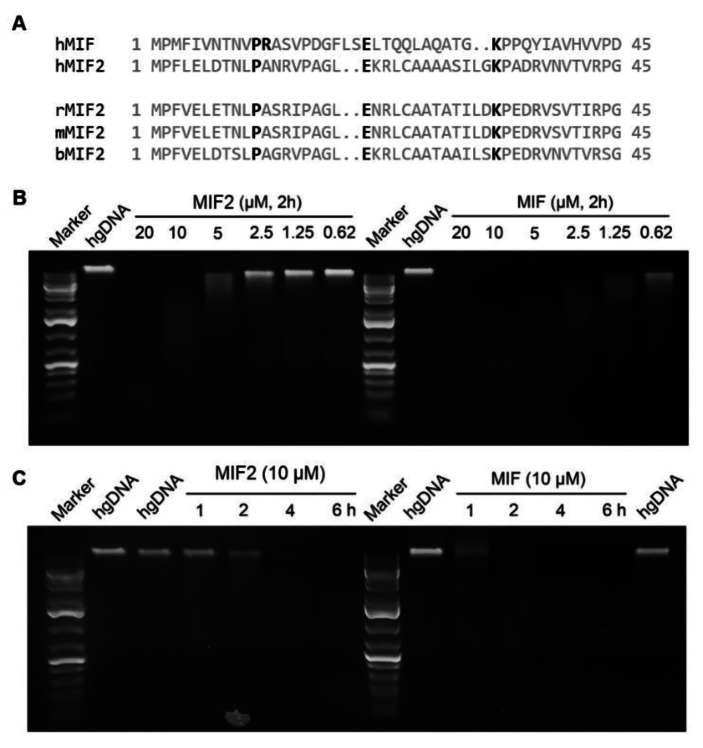
MIF and MIF2 possess nuclease activity and are able to cleave human genomic DNA. A) Sequence alignment of the anticipated nuclease domain of human MIF and MIF2 from different species. B) Concentration‐dependent activity of MIF and MIF2 on hgDNA. C) Time‐dependence for fragmentation of hgDNA upon treatment with MIF or MIF2.

As a next step, the MIF2 nuclease activity was experimentally confirmed. Towards this aim, hgDNA was purified from HeLa cells and incubated with recombinant human MIF2. The DNA samples were analyzed by agarose gel electrophoresis.^[18]^ The analysis indicates that MIF2 can effectively cleave hgDNA and that this cleavage is Mg^2+^‐dependent (Figures 5B, C, and S3). Mg^2+^ dependence is in line with MIF nuclease activity.^[18]^ Moreover, MIF2 cleaved hgDNA in a concentration‐ and time‐dependent manner. At concentrations above 10 μM, MIF2 provided complete DNA cleavage within 2 h. For comparison, the same experiment was done for MIF, which indicates that MIF is already able to fully fragment hgDNA at a concentration of 1.25 μM. This indicates that MIF2 is able to fragment hgDNA albeit with 10‐fold lower activity than MIF. Interestingly, MIF tautomerase activity towards *p*‐hydoxyphenylpyruvate is also ten‐fold higher than that of MIF2,^[6]^ however, tautomerase inhibitor **8** could not rescue DNA cleavage by MIF or MIF2 (Figure S3). This indicates that the nuclease active sites are different from the tautomerase active sites, in line with the previously reported observations by Wang et al.^[18]^

In conclusion, we demonstrate that 4‐iodopyrimidine is an effective warhead for labeling of MIF and MIF2. Using 4‐iodopyrimidine as a warhead, both MIF and MIF2 can be tagged with a fluorescent NBD label or with biotin. Differences in inhibitory kinetics can be exploited with the aim to gain insight in selective labeling of either MIF or MIF2. MIF‐directed labeling can be achieved by the use of a relatively low concentrations of the 4‐iodopyrimidine and relatively short incubation times, whereas MIF2‐directed labeling can be achieved by blocking MIF with a MIF‐selective covalent inhibitor followed by MIF2 labeling using relatively high concentrations of the 4‐iodopyrimidine and longer incubation times. This enables in situ visualization of MIF or MIF2 using confocal microscopy. Our observations that MNNG stimulation triggered nuclear translocation of both probe‐labeled MIF and MIF2 suggested that MIF2 can also act as a nuclease for which we gained indications by the observation of MIF2‐mediated fragmentation of hgDNA. Taken together, 4‐iodopyrimidine derived probes provide powerful tools to study the biology of MIF family proteins as exemplified here by the observed nuclear translocation of MIF and MIF2 in line with nuclease activity. This opens new opportunities to understanding the diverse functions of MIF family proteins and their exploitation as drug targets.

## Conflict of interest

The authors declare no conflict of interest.

## Supporting information

As a service to our authors and readers, this journal provides supporting information supplied by the authors. Such materials are peer reviewed and may be re‐organized for online delivery, but are not copy‐edited or typeset. Technical support issues arising from supporting information (other than missing files) should be addressed to the authors.

Supporting Information
