# Supplementary material for: 4‐Iodopyrimidine Labeling Reveals Nuclear Translocation and Nuclease Activity for Both MIF and MIF2
Source: Chemistry. 2021 Nov 22;28(1):e202103030. doi: 10.1002/chem.202103030 (PMC9299485; doi:10.1002/chem.202103030)

Chemistry–A European Journal

Supporting Information

4-Iodopyrimidine Labeling Reveals Nuclear Translocation and Nuclease Activity for Both MIF and MIF2

Zhangping Xiao, Deng Chen, Fabian Mulder, Shanshan Song, Petra E. van der Wouden, Robbert H. Cool, Barbro N. Melgert, Gerrit J. Poelarends, and Frank J. Dekker*

1. Synthesis
2. Protein production
3. Enzymatic inhibition
4. Label of MIF and MIF2 and mass spectra
5. Cell lysate labeling
6. Living cell labeling and imaging
7. Nuclease activity
8. References
9. NMR and mass spectra

1. Synthesis

General. All reagents and solvents were purchased from Sigma-Aldrich, TCI, Fluorochem or Acros and were used without further purification. The reactions were monitored by Thin Layer Chromatography (TLC), using Merck silica gel 60 F_{254} plates and the spots were detected with UV light. For purification using column chromatography, MP Ecochrom silica 32-63, 60 Å was used. Nuclear magnetic resonance spectra, ^{1}H NMR (500 MHz) and ^{13}C NMR (126MHz), were recorded on a Brucker Avance 500 spectrometer. Chemical shifts were reported in ppm. Chemical shifts were referred to the residual proton and carbon signals of deuterated solvent, $CDCl_3$: δ = 7.26 (^{1}H) and 77.05 ppm (^{13}C) or DMSO-d_6: δ = 2.50 (^{1}H) and 39.52 ppm (^{13}C). The following abbreviations were used for spin multiplicity: s (singlet), d (doublet), t (triplet), q (quartet), dd (double of doublets) and m (multiplet). Coupling constants were reported in hertz (Hz). High resolution mass spectra (HRMS) were recorded using Fourier transform mass spectrometry (FTMS) and electrospray ionization (ESI) on an Applied Biosystems/SCIEX API3000-triple quadruple mass spectrometer.[39]

Scheme S1. Synthesis of 4-IPP analogs[a]

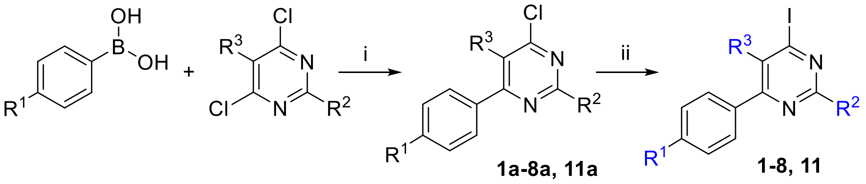

Compound	R^1	R^2	R^3
**1, 1a**	H	H	H
**2, 2a**	COOH	H	H
**3, 3a**	COOH	CH_3	H
**4, 4a**	COOH	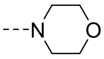	H
**5, 5a**	COOH	Ph	H
**6, 6a**	COOH	H	CH_3
**7, 7a**	NO_2	H	H
**11, 11a**	COOH	$CH(CH_3)_2$	H
**8a**	NH_2	H	H

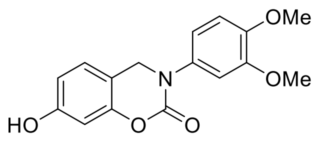

10 *

[a]Reagents and conditions: (i) tetrakis(triphenylphosphine)-palladium(0), sodium carbonate, water, ethylene glycol dimethyl ether, 92 °C reflux, 21-80%; (ii) hydroiodic acid, rt, 15-88%. *Structure of **10**. The synthesis and characterization of **10** was described in our previous work.[1]

General procedure for the Suzuki coupling reaction.[2] Phenylboronic acid derivative (1 mmol) was dissolved in ethylene glycol dimethyl ether (10 mL) together with 2 equivalents 4,6-dichloropyrimidine derivative (2 mmol) and tetrakis(triphenylphosphine)-palladium(0) (106 mg, 0.1 mmol). Sodium carbonate (305 mg, 2.9 mmol) was dissolved in 3 mL water and this solution was added to the reaction mixture. The mixture was refluxed overnight at 92°C. The resulting solution was diluted with ethyl acetate (50 mL) and was washed with HCl (0.5 N, 50 mL). The organic layer was collected and dried with $MgSO_4$. The suspension was filtered and the desired product was purified using column chromatography and was dried overnight in the desiccator.

General procedure for the iodination reaction.[3] The product from the Suzuki coupling reaction was collected and hydroiodic acid (2 mL, 57% w/w) was added. The mixture was stirred at room temperature overnight. The resulting mixture was centrifuged and the supernatant was discarded. The precipitate was washed three times with 1 mL water and was dried overnight in the lyophilizer.

4-Chloro-6-phenylpyrimidine (1a, FM002). Phenylboronic acid (121 mg, 1.0 mmol) was reacted with 4,6-dichloropyrimidine (312 mg, 2.1 mmol) following the general procedure for the Suzuki coupling reaction. 151 mg of the desired product was obtained as a white solid. Yield 79%. ^{1}H NMR (500 MHz, DMSO-d_6) δ 9.11 (s, 1H), 8.35 (s, 1H), 8.27 (d, J = 6.7 Hz, 2H), 7.59 (d, J = 7.3 Hz, 3H). ^{13}C NMR (126 MHz, DMSO-d_6) δ 165.4, 161.8, 159.5, 135.3, 132.5, 129.5, 127.9, 117.8. HRMS, calculated for $C_{10}H_8N_2Cl$ $[M + H]^+$: 191.0371, found 191.0371.

4-Iodo-6-phenylpyrimidine (1, FM004). FM002 (145 mg, 0.76 mmol) was reacted with hydroiodic acid (2 mL, 57% w/w) following the general procedure for the iodination reaction. 60 mg of the desired product was obtained as a yellow solid. Yield 28%. ^{1}H NMR (500 MHz, DMSO-d_6) δ 8.94 (s, 1H), 8.63 (s, 1H), 8.23 (d, J = 6.8 Hz, 2H), 7.57 (m, 3H). ^{13}C NMR (126 MHz, DMSO-d_6) δ 162.9, 159.0, 135.0, 132.6, 132.1, 129.7, 128.2, 127.8. HRMS, calculated for $C_{10}H_8N_2I$ $[M + H]^+$: 282.9727, found 282.9725.

4-(6-Chloropyrimidin-4-yl)benzoic acid (2a, FM001). 4-carboxyphenylboronic acid (169 mg, 1.0 mmol) was reacted with 4,6-dichloropyrimidine (313 mg, 2.1 mmol) following the general procedure for the Suzuki coupling reaction. 185 mg of the desired product was obtained as a white solid. Yield 79%. ^{1}H NMR (500 MHz, DMSO-d_6) δ 13.29 (s, 1H), 9.16 (s, 1H), 8.43 (s, 1H), 8.38 (d, J = 6.8 Hz, 2H), 8.10 (d, J = 8.6 Hz, 2H). ^{13}C NMR (126 MHz, DMSO-d_6) δ

167.2, 164.4, 162.0, 159.5, 139.1, 133.9, 130.4, 128.2, 118.5. HRMS, calculated for $C_{11}H_8O_2N_2Cl$ [M + H]$^+$: 235.0269, found 235.0267.

4-(6-Iodopyrimidin-4-yl)benzoic acid (2, FM003). FM001 (120 mg, 0.5 mmol) was reacted with hydroiodic acid (2 mL, 57% w/w) following the general procedure for the iodination reaction. 150 mg of the desired product was obtained as an orange solid. Yield 88%. ^{1}H NMR (500 MHz, DMSO-d_6) δ 8.98 (s, 1H), 8.69 (s, 1H), 8.34 (d, J = 6.5 Hz, 2H), 8.07 (d, J = 6.5 Hz, 2H). ^{13}C NMR (126 MHz, DMSO-d_6) δ 167.2, 161.8, 159.1, 139.0, 133.7, 132.8, 130.3, 129.0, 128.1. HRMS, calculated for $C_{11}H_8O_2N_2I$ [M + H]$^+$: 326.9625, found 326.9623.

4-(6-Chloro-2-methylpyrimidin-4-yl)benzoic acid (3a, FM005). 4-carboxyphenylboronic acid (167 mg, 1.0 mmol) was reacted with 4,6-dichloro-2-methylpyrimidine (326 mg, 2.0 mmol) following the general procedure for the Suzuki coupling reaction. 200 mg of the desired product was obtained as a white solid. Yield 80%. ^{1}H NMR (500 MHz, DMSO-d_6) δ 13.29 (s, 1H), 8.30 (d, J = 8.5 Hz, 2H), 8.17 (s, 1H), 8.11 (d, J = 8.5 Hz, 2H), 2.57 (s, 3H). ^{13}C NMR (126 MHz, DMSO) δ 172.7, 167.2, 165.1, 160.6, 139.0, 130.4, 129.3, 127.9, 116.6, 24.1. HRMS, calculated for $C_{12}H_{10}O_2N_2Cl$ [M + H]$^+$: 249.0425, found 249.0424.

4-(6-Iodo-2-methylpyrimidin-4-yl)benzoic acid (3, FM006). FM005 (160 mg, 0.64 mmol) was reacted with hydroiodic acid (2 mL, 57% w/w) following the general procedure for the iodination reaction. 33 mg of the desired product was obtained as a yellow solid. Yield 15%. ^{1}H NMR (500 MHz, DMSO-d_6) δ 13.28 (s, 1H), 8.24 (d, J = 8.6 Hz, 2H), 8.14 (s, 1H), 8.10 (d, J = 8.6 Hz, 2H), 2.50 (s, 3H). ^{13}C NMR (126 MHz, DMSO) δ 171.2, 167.2, 163.7, 139.1, 133.7, 131.2, 130.4, 127.8, 117.1, 22.9. HRMS, calculated for $C_{12}H_{10}O_2N_2I$ [M + H]$^+$: 340.9781, found 340.9778.

4-(6-Chloro-2-morpholinopyrimidin-4-yl)benzoic acid (4a, FM009). 4-carboxyphenylboronic acid (168 mg, 1.0 mmol) was reacted with 4-(4,6-dichloromethylpyrimidine-2-yl)morpholine (472 mg, 2.0 mmol) following the general procedure for the Suzuki coupling reaction. 182 mg of the desired product was obtained as a light yellow solid. Yield 57%. ^{1}H NMR (500 MHz, DMSO-d_6) δ 13.25 (s, 1H), 8.29 (d, J = 8.4 Hz, 2H), 8.05 (d, J = 8.4 Hz, 2H), 7.47 (s, 1H), 3.73 – 3.69 (m, 4H), 3.36 (m, 4H). ^{13}C NMR (126 MHz, DMSO-d_6) δ 167.3, 164.9, 162.1, 161.4, 140.0, 133.5, 130.1, 127.9, 106.0, 66.3, 44.5. HRMS, calculated for $C_{15}H_{15}O_3N_3Cl$ [M + H]$^+$: 320.0796, found 320.0795.

4-(6-Iodo-2-morpholinopyrimidin-4-yl)benzoic acid (4, FM010). FM009 (95 mg, 0.30mmol) was reacted with hydroiodic acid (2 mL, 57% w/w) following the general procedure

for the iodination reaction. 90 mg of the desired product was obtained as a yellow/orange solid. Yield 73%. ^{1}H NMR (500 MHz, DMSO-d_6) δ 8.26 (d, *J* = 8.5 Hz, 2H), 8.04 (d, *J* = 8.6 Hz, 2H), 7.77 (s, 1H), 3.80 – 3.76 (m, 4H), 3.70 (m, 4H). ^{13}C NMR (126 MHz, DMSO-d_6) δ 167.3, 164.9, 162.4, 160.6, 139.7, 133.0, 130.1, 127.8, 116.9, 66.4, 44.4. HRMS, calculated for $C_{15}H_{15}O_3N_3I$ [M + H]$^+$: 412.0153, found 412.0146.

4-(6-Chloro-2-phenylpyrimidin-4-yl)benzoic acid (5a, FM011). 4-carboxyphenylboronic acid (169 mg, 1.0 mmol) was reacted with 4,6-dichloro-2-phenylpyrimidine (552 mg, 2.5 mmol) following the general procedure for the Suzuki coupling reaction. 65 mg of the desired product was obtained as a white solid. Yield 21%. ^{1}H NMR (500 MHz, DMSO-d_6) δ 13.32 (s, 1H), 8.52 – 8.47 (m, 4H), 8.32 (s, 1H), 8.13 (d, *J* = 8.5 Hz, 2H), 7.61 (m, 3H). ^{13}C NMR (126 MHz, DMSO-d_6) δ 167.3, 164.7, 164.6, 162.5, 139.4, 136.2, 133.9, 132.2, 130.4, 129.3, 128.6, 128.3, 116.3. HRMS, calculated for $C_{17}H_{12}O_2N_2Cl$ [M + H]$^+$: 311.0582, found 311.058.

4-(6-Iodo-2-phenylpyrimidin-4-yl)benzoic acid (5, FM012). FM011 (50 mg, 0.16 mmol) was reacted with hydroiodic acid (2 mL, 57% w/w) following the general procedure for the iodination reaction. 32 mg of the desired product was obtained as a white solid. Yield 50%. ^{1}H NMR (500 MHz, DMSO-d_6) δ 8.62 (s, 1H), 8.52 – 8.44 (m, 4H), 8.12 (d, *J* = 8.4 Hz, 2H), 7.59 (m, 3H). ^{13}C NMR (126 MHz, DMSO-d_6) δ 167.4, 164.1, 162.1, 139.2, 136.2, 133.9, 133.5, 130.4, 129.5, 129.3, 128.6, 128.2, 126.7. HRMS, calculated for $C_{17}H_{12}O_2N_2I$ [M + H]$^+$: 402.9938, found 402.9931.

4-(6-Chloro-5-methylpyrimidin-4-yl)benzoic acid (6a, FM015). 4-carboxyphenylboronic acid (167 mg, 1.0 mmol) was reacted with 4,6-dichloro-2-methylpyrimidine (326 mg, 2.0 mmol) following the general procedure for the Suzuki coupling reaction. 150 mg of the desired product was obtained as a white solid. Yield 60 %. ^{1}H NMR (500 MHz, DMSO-d_6) δ 13.22 (s, 1H), 8.96 (s, 1H), 8.08 (d, *J* = 8.3 Hz, 2H), 7.75 (d, *J* = 8.3 Hz, 2H), 2.36 (s, 3H). ^{13}C NMR (126 MHz, DMSO) δ 166.9, 165.7, 161.7, 155.5, 141.2, 131.7, 129.5, 129.3, 128.0, 16.5. HRMS, calculated for $C_{12}H_{10}O_2N_2Cl$ [M + H]$^+$: 249.0425, found 249.0424.

4-(6-Iodo-5-methylpyrimidin-4-yl)benzoic acid (6, FM016). FM005 (120 mg, 0.5 mmol) was reacted with hydroiodic acid (2 mL, 57% w/w) following the general procedure for the iodination reaction. 90 mg of the desired product was obtained as a yellow solid. Yield 56%. . ^{1}H NMR (500 MHz, DMSO-d_6) δ 13.20 (s, 1H), 8.74 (s, 1H), 8.06 (d, *J* = 8.1 Hz, 2H), 7.72 (d, *J* = 8.1 Hz, 2H), 2.36 (s, 3H). ^{13}C NMR (126 MHz, DMSO) δ 167.3, 163.2, 156.0, 142.2, 140.5,

134.9, 132.0, 129.9, 129.7, 24.0. HRMS, calculated for $C_{12}H_{10}O_2N_2I$ $[M + H]^+$: 340.9781, found 340.9775.

4-Chloro-6-(4-nitrophenyl)pyrimidine (7a, FM007). 4-nitrophenylboronic acid (169 mg, 1.0 mmol) was reacted with 4,6-dichloro-2-methylpyrimidine (298 mg, 2.0 mmol) following the general procedure for the Suzuki coupling reaction. 182 mg of the desired product was obtained as a white solid. Yield 77%. ^{1}H NMR (500 MHz, DMSO-d_6) δ 9.22 (s, 2H), 8.53 (d, J = 8.6 Hz, 4H), 8.41 (d, J = 1.6 Hz, 2H), 8.40 (s, 1H). ^{13}C NMR (126 MHz, DMSO-d_6) δ 163.2, 162.3, 159.7, 149.8, 141.2, 129.4, 124.6, 119.3. HRMS, calculated for $C_{10}H_7O_2N_3Cl$ $[M + H]^+$: 236.0221, found 236.022.

4-Iodo-6-(4-nitrophenyl)pyrimidine (7, FM008). FM007 (100 mg, 0.42 mmol) was reacted with hydroiodic acid (2 mL, 57% w/w) following the general procedure for the iodination reaction. 68 mg of the desired product was obtained as an orange solid. Yield 50%. ^{1}H NMR (500 MHz, DMSO-d_6) δ 9.04 (s, 1H), 8.79 (s, 1H), 8.50 (d, J = 8.9 Hz, 3H), 8.38 (d, J = 8.8 Hz, 2H). ^{13}C NMR (126 MHz, DMSO) δ 160.6, 159.2, 149.7, 141.1, 133.2, 129.5, 126.1, 124.5. HRMS, calculated for $C_{10}H_7O_2N_3I$ $[M + H]^+$: 327.9577, found 327.9575.

4-(6-Chloropyrimidin-4-yl)aniline (8a, ZP308). 4-aminophenylboronic acid (350 mg, 2.0 mmol) was reacted with 4,6-dichloro-2-phenylpyrimidine (450 mg, 3.0 mmol) following the general procedure for the Suzuki coupling reaction. 390 mg of the desired product was obtained as a pale-yellow solid. Yield 95%.^{1}H NMR (500 MHz, DMSO) δ 8.86, 8.00, 7.99, 7.98, 6.66, 6.64, 5.98. ^{13}C NMR (126 MHz, DMSO) δ 165.6, 160.8, 159.1, 153.3, 129.6, 129.3, 121.6, 114.8, 114.5, 114.0. HRMS, calculated for $C_{10}H_9N_3Cl$ $[M + H]^+$: 206.048, found 206.0479.

4-(6-Chloro-2-isopropylpyrimidin-4-yl)benzoic acid (11a, FM023). 4-carboxyphenylboronic acid (167 mg, 1.0 mmol) was reacted with 4,6-dichloro-2-isopropylpyrimidine (390 mg, 2.0 mmol) following the general procedure for the Suzuki coupling reaction. 140 mg of the desired product was obtained as a white solid. Yield 51%. ^{1}H NMR (500 MHz, DMSO-d_6) δ 13.29 (s, 1H), 8.38 (d, J = 8.6 Hz, 2H), 8.22 (s, 1H), 8.10 (d, J = 8.6 Hz, 2H), 3.21 (m, 1H), 1.34 (d, J = 6.9 Hz, 6H). ^{13}C NMR (126 MHz, DMSO) δ 176.1, 167.3, 164.4, 162.0, 139.5, 133.8, 130.3, 128.2, 115.6, 37.3, 21.8. HRMS, calculated for $C_{14}H_{14}O_2N_2Cl$ $[M + H]^+$: 277.0738, found 277.0738.

4-(6-Iodo-2-isopropylpyrimidin-4-yl)benzoic acid (11, FM024). FM023 (100 mg, 0.36 mmol) was reacted with hydroiodic acid (2 mL, 57% w/w) following the general procedure for the iodination reaction. 46 mg of the desired product was obtained as a light yellow solid. Yield

35%. ^{1}H NMR (500 MHz, DMSO-d_6) δ 13.27 (s, 1H), 8.49 (s, 1H), 8.35 (d, *J* = 8.5 Hz, 2H), 8.08 (d, *J* = 8.5 Hz, 2H), 3.16 (m, 1H), 1.32 (d, *J* = 6.9 Hz, 6H). ^{13}C NMR (126 MHz, DMSO) δ 175.6, 167.3, 161.8, 139.3, 133.6, 133.1, 130.3, 128.1, 126.1, 37.2, 21.9. HRMS, calculated for $C_{14}H_{14}O_2N_2I$ $[M + H]^+$: 369.0094, found 369.0091.

Scheme S2. Synthesis of probes[a]

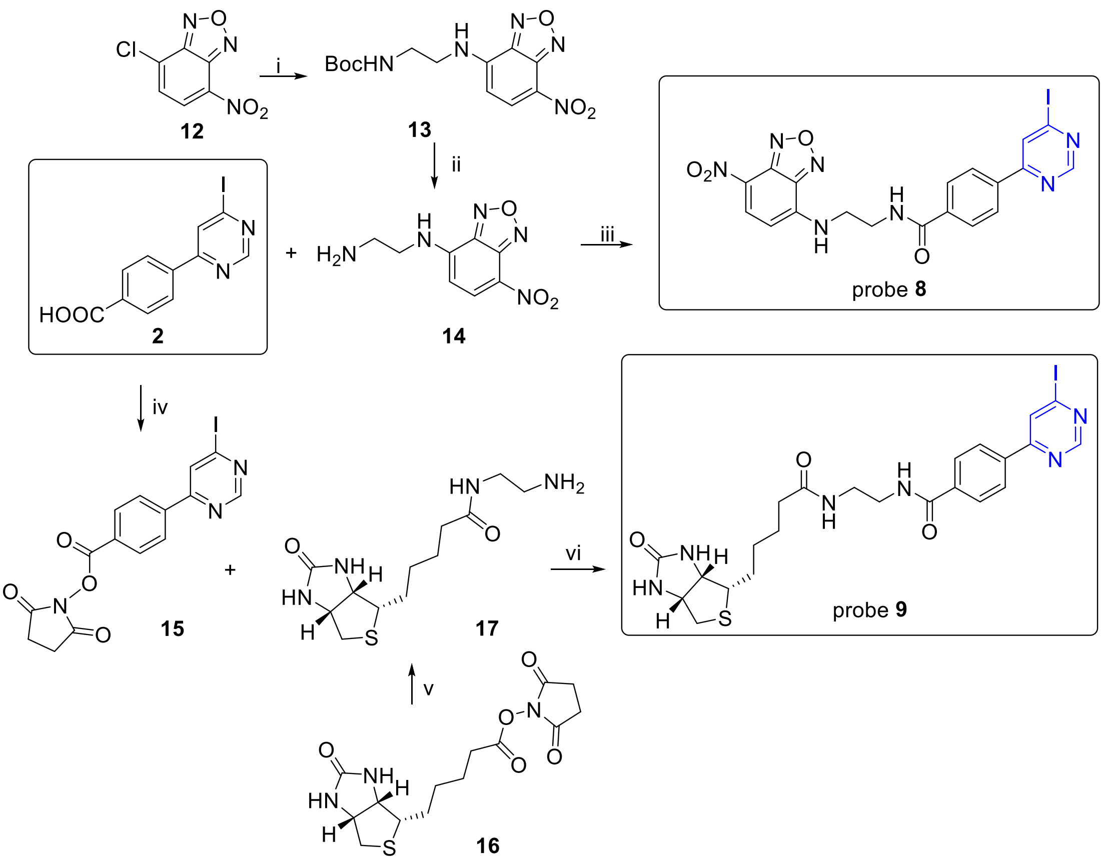

[a]Reagents and conditions: (**i**) N-Boc-ethylenediamine, acetonitrile, rt, 4h; (**ii**) TFA, CH_2Cl_2, reflux, 2h; (**iii**) HOBt, EDCI, DMF, rt, overnight; (iv) N-hydroxysuccinimide, EDCI, ethyl acetate, rt overnight; (v) a. N-Boc-ethylenediamine, DMF, rt, overnight; b. TFA, CH_2Cl_2, reflux, 2h; (vi) TEA, CH_2Cl_2, 55°C, overnight.

***tert*-Butyl (2-((7-nitrobenzo[c][1,2,5]oxadiazol-4-yl)amino)ethyl)carbamate (13, ZP254).** 4-Chloro-7-nitrobenzofurazan (430 mg, 2.1 mmol) was dissolved into acetonitrile (10 mL). To this solution, *N*-Boc-ethylenediamine (0.5 mL, 3.1 mmol) was added and the resulting mixture was stirred 4 hours at room temperature. The product was purified with chromatography using CH_2Cl_2: MeOH 100:1 (*v/v*) as eluence to obtain 420 mg orange solid as product, yield 66%. ^{1}H NMR (500 MHz, Chloroform-*d*) δ 8.49 (d, *J* = 8.6 Hz, 1H), 7.70 (s, 1H), 6.19 (d, *J* = 8.6 Hz,

1H), 5.13 (s, 1H), 3.64 – 3.61 (m, 7H), 1.48 (s, 8H). ^{13}C NMR (126 MHz, $CDCl_3$) δ 157.8, 144.4, 144.3, 143.9, 136.6, 123.8, 98.4, 81.0, 46.0, 39.1, 28.3.

***N^1*-(7-nitrobenzo[c][1,2,5]oxadiazol-4-yl)ethane-1,2-diamine (14, ZP306).** The Boc-protected ZP254 (160 mg, 0.5 mmol) was dissolved in CH_2Cl_2 (10mL), into which TFA (2 mL) was added. The mixture was refluxed for 2 hours. The deprotected product was obtained after chromatography purification using CH_2Cl_2: MeOH 20:1 (v/v) as eluence as 98 mg yellow solid, yield 88%. ^{1}H NMR (500 MHz, DMSO-d_6) δ 8.58 (d, *J* = 8.8 Hz, 1H), 6.50 (d, *J* = 9.0 Hz, 1H), 3.76 (s, 2H), 3.17 (t, *J* = 6.3 Hz, 2H), 2.51 (t, *J* = 1.6 Hz, 2H). ^{13}C NMR (126 MHz, DMSO) δ 145.6, 145.0, 144.5, 138.3, 118.9, 99.7, 41.2, 37.7.

2,5-Dioxopyrrolidin-1-yl 4-(6-chloro-2-methylpyrimidin-4-yl)benzoate (15, ZP330). FM005 (50 mg, 0.20 mmol), N-hydroxysuccinimide (24 mg, 0.21 mmol) and N-(3-Dimethylaminopropyl)-N-ethylcarbodiimide hydrochloride (77 mg, 0.40 mmol) were dissolved in ethyl acetate (10 mL). The mixture was stirred at room temperature overnight. The resulting mixture was diluted in ethyl acetate (100 mL) and washed with water (50 mL) and brine (50 mL). The organic layer was collected and dried with $MgSO_4$. The suspension was filtered. The organic solvent was removed under reduced pressure by a rotary evaporator and the desired product was purified using column chromatography and was dried overnight in the desiccator. The desired product was obtained as a solid. ^{1}H NMR (500 MHz, Chloroform-*d*) δ 8.28 (d, *J* = 8.5 Hz, 1H), 8.23 (d, *J* = 8.5 Hz, 1H), 7.65 (s, 0H), 2.99 – 2.92 (m, 2H), 2.82 (s, 1H). ^{13}C NMR (126 MHz, $CDCl_3$) δ 169.6, 169.1, 163.9, 162.1, 161.3, 141.6, 132.6, 131.2, 127.8, 114.8, 26.1, 25.7.

***N*-(2-aminoethyl)-5-(2-oxohexahydro-1H-thieno[3,4-d]imidazol-4-yl)pentanamide (17, FM028).** Biotinyl-OSu (535 mg, 1.5 mmol) was dissolved in dry dimethylformamide (8 mL). N-Boc-ethylenediamine (255 µL, 1.5 mmol) was added and the mixture was stirred at room temperature overnight. The resulting mixture was diluted with CH_2Cl_2 (80 mL) and washed with water (50 mL), saturated ammonium chloride solution (50 mL), saturated sodium bicarbonate (50 mL), and brine(50 mL). The organic layer was collected and dried with $MgSO_4$. The suspension was filtered. The organic solvent was removed under reduced pressure by a rotary evaporator and the desired product was purified using column chromatography and was dried overnight in the desiccator. The obtained product was deprotected with TFA to remove the Boc group. 30 mg of the desired product was obtained as a white solid. Yield: 10%. ^{1}H NMR (500 MHz, DMSO-d_6) δ 7.80 (t, *J* = 5.7 Hz, 1H), 6.80 (t, *J* = 5.7 Hz, 1H), 6.44 (s, 1H), 6.38 (s, 1H), 4.33 – 4.29 (m, 1H), 4.15 – 4.12 (m, 1H), 3.14 – 3.08 (m, 1H), 3.05 (q, *J* =

6.4 Hz, 2H), 2.96 (q, *J* = 6.5 Hz, 2H), 2.83 (d, *J* = 7.4 Hz, 1H), 2.58 (d, *J* = 12.5 Hz, 1H), 2.05 (t, *J* = 7.5 Hz, 2H), 1.65 – 1.41 (m, 3H), 1.35 – 1.23 (m, 2H). ^{13}C NMR (126 MHz, DMSO) δ 172.6, 163.2, 156.1, 78.1, 59.6, 55.9, 40.2, 39.1, 35.7, 29.1, 28.7, 25.7.

4-(6-Iodopyrimidin-4-yl)-*N*-(2-((7-nitrobenzo[c][1,2,5]oxadiazol-4-yl)amino)ethyl)benzamide (8, ZP307). ZP306 (36 mg, 0.15 mmol) was reacted with FM003 (50mg, 0.15 mmol) in presence with EDCI (58 mg, 0.3 mmol) and HOBt (13 mg, 0.1 mmol) in DMF (1 mL) at rt for overnight. The reaction mixture was diluted in CH_2Cl_2 (40 mL), which was washed with brine (3 × 20 mL). The organic layer was collected and dried over $MgSO_4$ before evaporation under reduced pressure. The desired product was purified with chromatography using CH_2Cl_2: MeOH 60:1 (*v/v*) as eluence. 50 mg yellow solid was obtained as product, yield 65%. ^1H NMR (500 MHz, DMSO-d_6) δ 9.55 (s, 1H), 8.97 (s, 1H), 8.87 (s, 1H), 8.69 (s, 1H), 8.54 (d, *J* = 8.5 Hz, 1H), 8.33 (d, *J* = 8.2 Hz, 2H), 7.97 (d, *J* = 8.3 Hz, 2H), 6.52 (d, *J* = 8.9 Hz, 1H), 3.72 – 3.61 (m, 4H). ^{13}C NMR (126 MHz, DMSO) δ 166.0, 161.4, 158.6, 145.4, 144.50, 144.1, 137.86, 137.1, 136.7, 132.2, 128.2, 127.9, 127.8, 127.4, 120.9, 42.8, 37.9. HRMS, calculated for $C_{19}H_{15}O_4N_7I$ $[M + H]^+$: 532.0225, found 532.0225.

4-(6-Iodopyrimidin-4-yl)-*N*-(2-(5-(2-oxohexahydro-1*H*-thieno[3,4-d]imidazol-4-yl)pentanamido)ethyl)benzamide (9, FM031). FM028 (30 mg, 0.10 mmol) was dissolved in CH_2Cl_2 (10 mL). Trifluoroacetic acid (2 mL) was added and the mixture was stirred at room temperature for 1 hour. The acidified organic solvent was removed under reduced pressure by a rotary evaporator. The solid was washed with CH_2Cl_2 (10 mL) which was subsequently removed under reduced pressure by a rotary evaporator, this was done three times. FM033 (40 mg, 0.1 mmol) was added to the deprotected FM028 and subsequently CH_2Cl_2 (10 mL) and triethylamine (50 µL) were added. The mixture was refluxed at 55°C overnight. The resulting mixture was diluted with CH_2Cl_2 (to 50 mL) and washed with water (50 mL). The desired product precipitated in water and the suspension was filtrated. The precipitate was washed with CH_2Cl_2 (2 mL) and dried. 20 mg of the desired product was obtained as a white solid. Yield: 36%. ^1H NMR (500 MHz, DMSO-d_6) δ 8.98 (s, 1H), 8.71 (s, 1H), 8.67 (t, *J* = 5.5 Hz, 1H), 8.33 (d, *J* = 8.3 Hz, 2H), 8.00 (d, *J* = 8.3 Hz, 2H), 7.95 (t, *J* = 5.9 Hz, 2H), 6.43 (s, 1H), 6.36 (s, 1H), 4.31 – 4.25 (m, 1H), 4.12 – 4.06 (m, 1H), 3.32 (s, 3H), 3.25 (q, *J* = 5.7 Hz, 3H), 3.08 – 3.02 (m, 1H), 2.79 (d, *J* = 7.2 Hz, 1H), 2.57 (d, *J* = 12.5 Hz, 2H), 2.11 – 2.05 (m, 3H), 1.64 – 1.54 (m, 2H), 1.45 (m, 2H), 1.35 – 1.25 (m, 3H). ^{13}C NMR (126 MHz, DMSO) δ 172.9, 166.1, 163.2, 162.0, 159.1, 137.5, 137.4, 132.7, 128.7, 128.3, 127.8, 61.4, 59.7, 55.9, 40.4, 39.7, 38.6, 35.7, 28.6, 28.5, 25.7. HRMS, calculated for $C_{23}H_{28}O_3N_6IS$ $[M + H]^+$: 595.0983, found 595.0982.

2. Preparation of recombinant MIF and MIF2.

The detailed procedure to produce C-terminal His-tagged recombinant human MIF was reported in our previous publications.[1,4,5] rhMIF was expressed through transforming a pET-20b(+) plasmid containing the target gene into *Escherichia coli* BL21 (DE3) according to literature procedures.[6] Overexpression was performed and cells were collected and sonicated. The obtained supernatant was applied to a medium pressure chromatography system (Biologic Duoflow) equipped with a His trap HP (5 mL) column with detection at 280 nm for the eluent. The column was washed with a binding buffer containing 50 mM Tris and 10% glycerol at pH 7.4 and eluted with elution buffer containing 500 mM imidazole, 50 mM Tris, 10% glycerol at pH 7.4. The pure fractions (as judged by SDS-PAGE) were pooled and subjected to a PD-10 column (GE healthcare) that was equilibrated with PBS buffer at pH 7.4 to remove the high concentration of imidazole. The collected MIF solution was divided into 50 µL aliquots and stored at –80 °C. Purity of obtained protein was tested by SDS-PAGE and Coomassie staining. The concentration of protein was determined using the Bradford assay.

The detailed procedure to produce recombinant human MIF2 was reported in our previous publications.[7] Gene sequences of the human MIF2 gene (Invitrogen) was adapted to bacterial expression. After subcloning into a pET20b(+) expression vector, IPTG-induced expression was performed in *E.coli* strain BL21(DE3). MIF2 proteins were overproduced overnight and harvested cells were resuspended and sonicated. The soluble fraction was purified using a Q sepharose column (GE Healthcare) with a gradient of NaCl. The fractions containing MIF2 were brought to 1.7 M ammonium sulfate and loaded on a phenyl sepharose column (GE Healthcare) and eluted with a gradient to 0 M ammonium sulfate in a 20 mM sodium phosphate buffer, pH 8.0. Finally, the proteins were purified by size exclusion chromatography on a Superdex75 column (GE Healthcare) in 20 mM sodium phosphate buffer, pH 8.0, with an elution volume characteristic for trimeric MIF2. The collected protein was concentrated using a VivaSpin centrifugation column with a molecular weight cut off at 5000 Da (Sartorius Stedim Biotech GmbH). Purified proteins were aliquoted, snap frozen in liquid nitrogen, and stored at –80 °C. Purity of obtained protein was tested by SDS-PAGE and coomassie staining. The concentration of protein was determined using the Bradford assay.

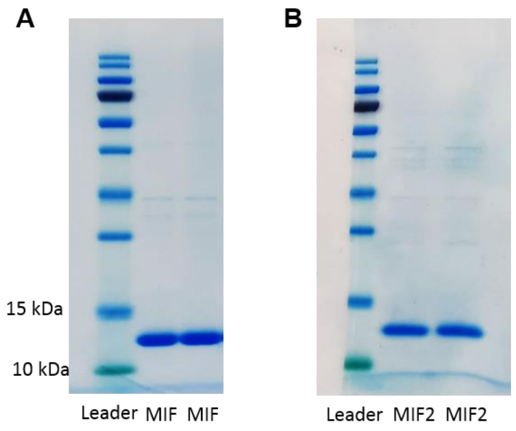

Figure S1. Characterization of the purity of produced MIF and MIF2 using SDS-PAGE separation and coomassie protein staining. (A) Bands of MIF; (B) Bands of MIF2.

3. Enzymatic assays

3.1 MIF and MIF2 Inhibition

The protocol for measuring inhibition of MIF or MIF2 tautomerase enzyme activity was described previously.[5] 500 nM MIF solution (180 µL) or 100 nM MIF2 solution (180 µL) in boric acid buffer (435 nM, pH 6.2) was mixed with 20 mM EDTA solution (10 µL) in demineralized water. A solution of the desired compound dissolved in DMSO (10 µL) or blank DMSO (10 µL) was added. The resulting mixture was preincubated at room temperature for 10 minutes. Subsequently, 50 µL of this mixture was mixed with 50 µL of a 1 mM 4-HPP solution for MIF or 1 mM PP solution for MIF2 in ammonium acetate buffer (50 mM, pH 6.0). Thereafter, MIF or MIF2 tautomerase activity was monitored by measuring the increase of UV absorbance at 306 nm for MIF or 300 nm for MIF2 over time. The MIF or MIF2 tautomerase activity of the mixture with blank DMSO dilution was set to 100% enzyme activity. Noncatalyzed conversion of the substrate in the mixture without MIF or MIF2 was set to 0%. The data acquired from the first 3 minutes of the measurement were used to calculate the initial conversion speed. All experiments were performed in triplicate and data processing was done using the program Graphpad Prism 8.

Table S1. IC_{50} values (10 min) and inactivation parameters of synthesized compounds.

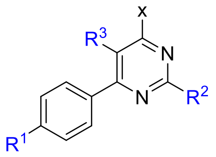

Compound	X	R1	R2	R3	IC_{50} (µM)	
					MIF	MIF2
**1 (4-IPP)**	-I	-H	-H	-H	4,0 (±0,98)	>250
**1a**	-Cl	-H	-H	-H	16 (±3.2)	>250
**2a**	-Cl	-COOH	-H	-H	17 (±3.3)	>250
**2**	-I	-COOH	-H	-H	5,0 (±0.46)	125 (±15)
**3a**	-Cl	-COOH	$-CH_3$	-H	95 (±44)	103 (±12)
**3**	-I	-COOH	$-CH_3$	-H	>250	>250
**7a**	-Cl	$-NO_2$	-H	-H	1.4 (±0.27)	>250
**7**	-I	$-NO_2$	-H	-H	0.63 (±0.06)	>250

**4a**	-Cl	-COOH	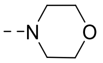	-H	174 (±27)	>250
**4**	-I	-COOH	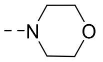	-H	15 (±7.5)	>250
**5a**	-Cl	-COOH	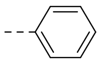	-H	36 (±4.8)	85 (±4.5)
**5**	-I	-COOH	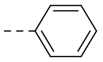	-H	59 (±6.3)	>250
**6a**	-Cl	-COOH	-H	$-CH_3$	>250	>250
**6**	-I	-COOH	-H	$-CH_3$	56 (± 12)	>250
**11a**	-Cl	-COOH	$-CH(CH_3)_2$	-H	>250	>250
**11**	-I	-COOH	$-CH(CH_3)_2$	-H	112 (± 12)	>250
**8a**	-Cl	$-NH_2$	-H	-H	1.7 (± 0.22)	83 (±7.9)

Table S2. Dose-inhibition curves and IC_{50} values of MIF tautomerase inhibitors.

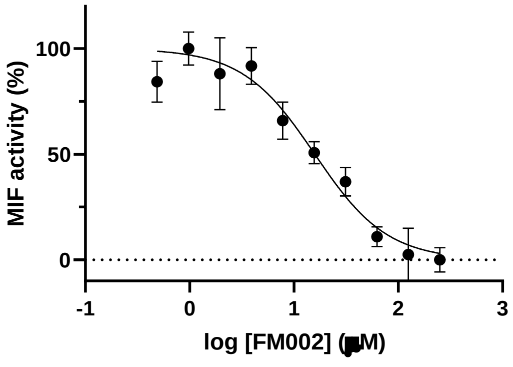

1a, FM002: IC_{50}=16±3.2 μM

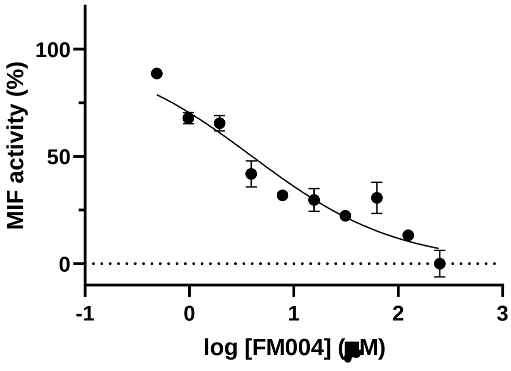

1, FM004: IC_{50}=4.0±0.98 μM

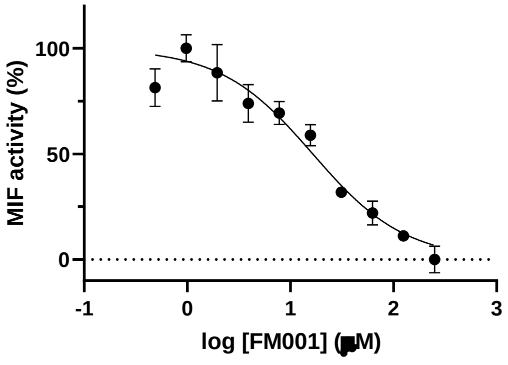

2a, FM001: IC_{50}=17±3.3 μM

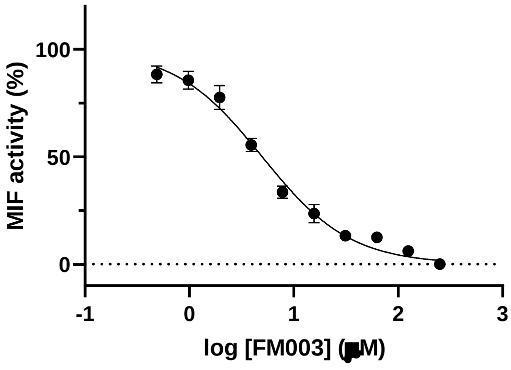

2, FM003: IC_{50}=5.0±0.46 μM

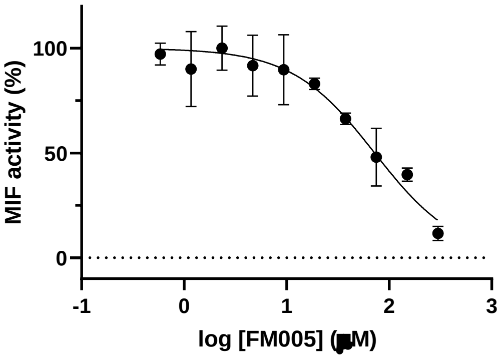

3a, FM005: IC$_{50}$=72±17 µM

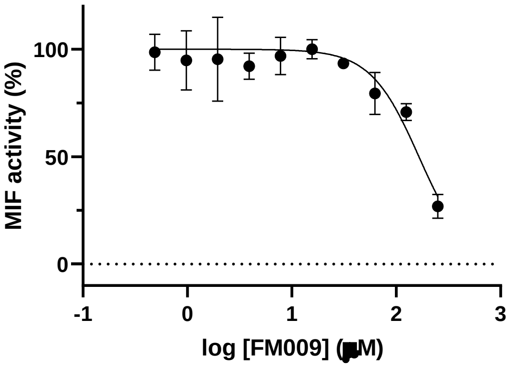

4a, FM009: IC$_{50}$=165±28 µM

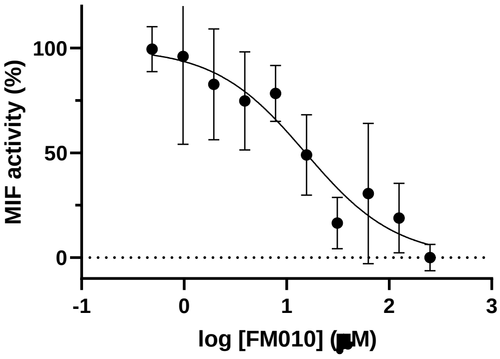

4, FM010: IC$_{50}$=15±7.5 µM

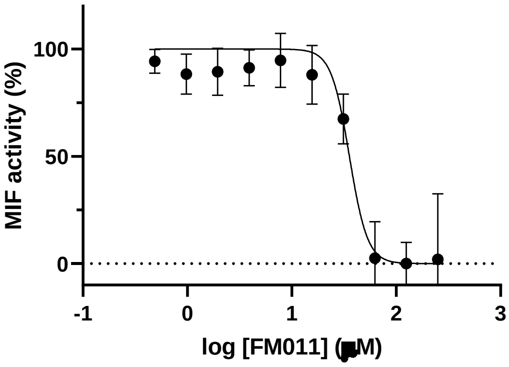

5a, FM011: IC$_{50}$=36±4.8 µM

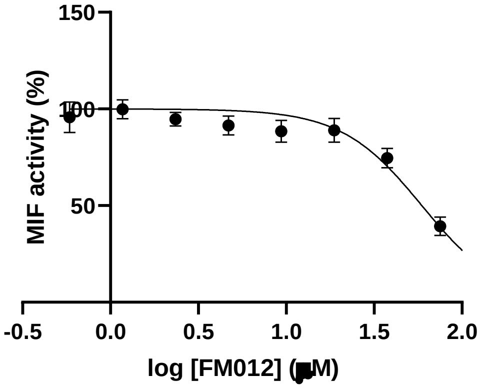

5, FM012: IC$_{50}$=59±6.3 µM

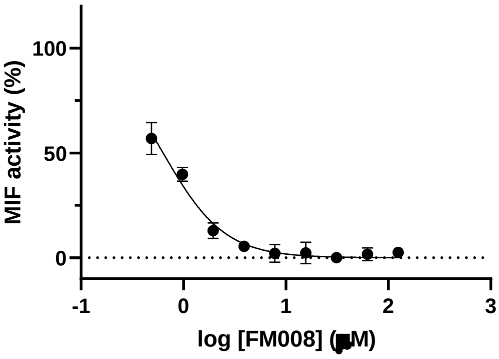

7, FM008: IC$_{50}$=0.63±0.061 µM

<table>
<tr><td>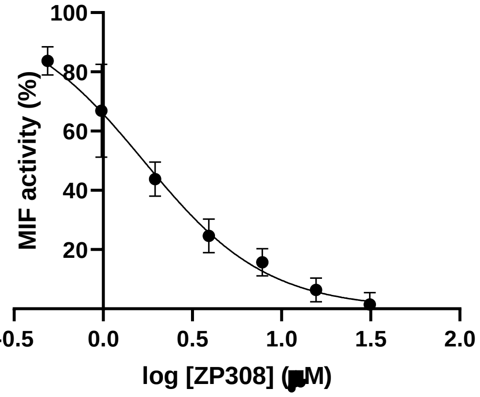
</td><td>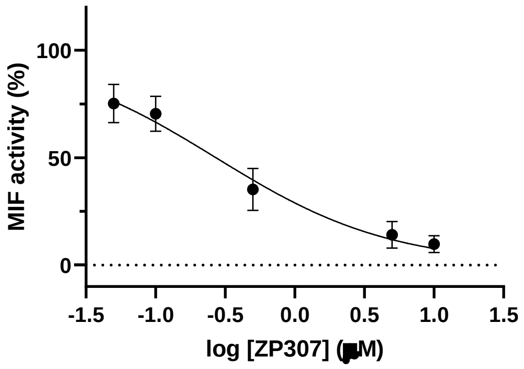
</td></tr>
<tr><td>8a, ZP308: IC_{50}=1.7± 0.22 μM</td><td>8, ZP307: IC_{50}=0.27±0.085 μM</td></tr>
<tr><td>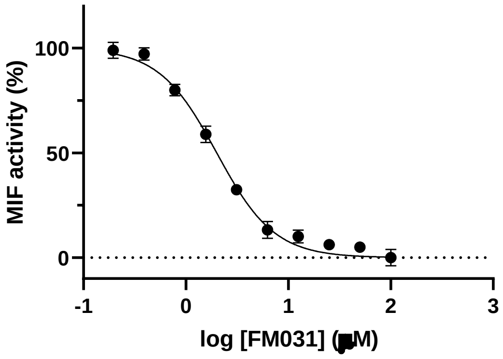
</td><td>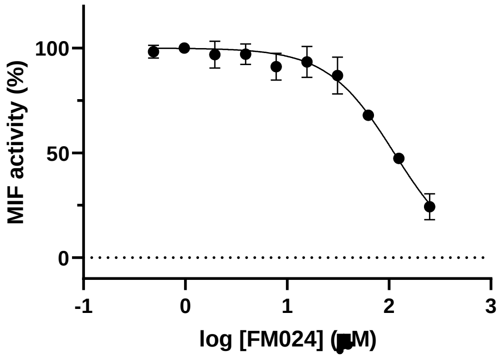
</td></tr>
<tr><td>9, FM031: IC_{50}=2.0±0.14 μM</td><td>11, FM024: IC_{50}=112±12 μM</td></tr>
</table>

Table S3. Dose-inhibition curves and IC_{50} values of MIF2 tautomerase inhibitors.

<table>
<tr><td>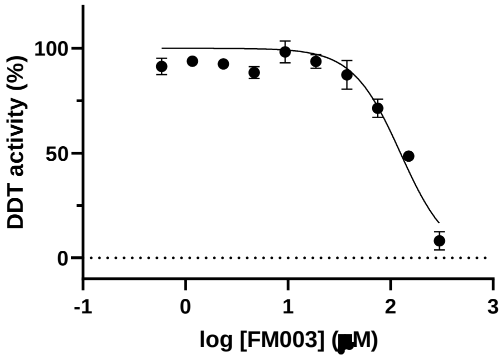
</td><td>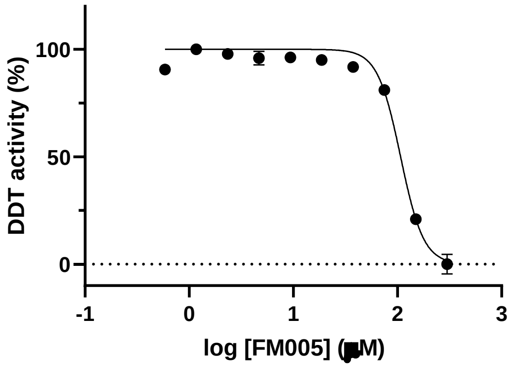
</td></tr>
<tr><td>2, FM003: IC_{50}=125±15</td><td>3a, FM005: IC_{50}=107±6.0</td></tr>
</table>

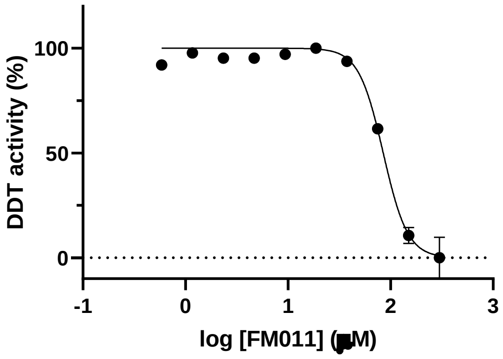

5a, FM011: IC_{50}=85±4.5

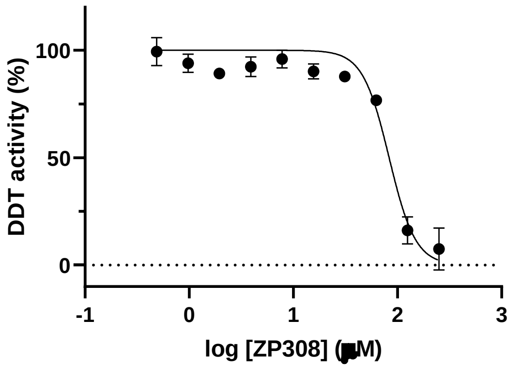

8a, ZP308: IC_{50}=83±7.9

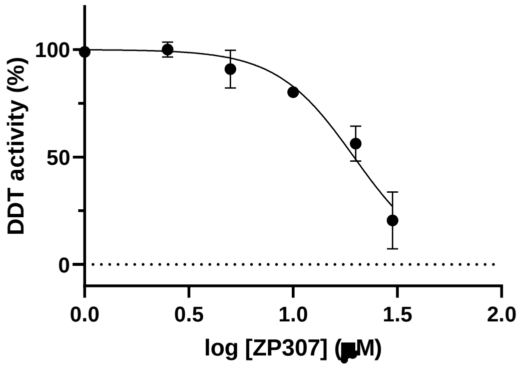

8, ZP307: IC_{50}=20±2.2

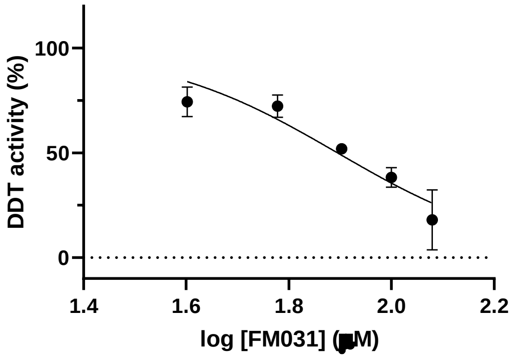

9, FM031: IC_{50}=78±7.7

3.3 Kinetics studies

In order to derive the kinetic parameters for inactivation of MIFand MIF2, we employed the approach described by Kitz and Wilson. Briefly, four different concentrations of the inhibitor with four different pre-incubation times were selected. The assay conditions are described above with the difference in incubation time.

For the data analysis, the pre-incubation times for the different concentration of the inhibitor versus the remaining enzyme activity were plotted, so from the linear regression the slope of each concentration of the probe was obtained (Table S3). Then the 1/slope versus 1/[inhibitor] were plotted (Figure 4) and from the linear regression line ki was derived from the 1/y-intercept and derived from the slope times the ki as described by Kitz and Wilson.

For example, for the probe **8** the equation is: Y = 7,476*X + 5,069

So, the k_{inact} and the K_I values are: k_{inact} =1/5,069 =0.20 min^{-1}

$K_I = k_{inact} * 7.467=1.5$

Table S4. Kinetics studies results.

**Time-dependent inhibition**	**Kitz and Wilson curve**
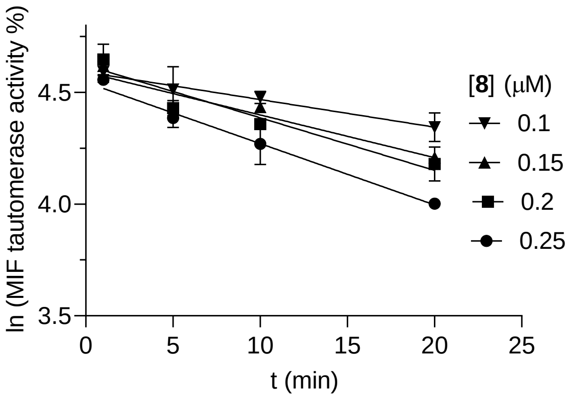	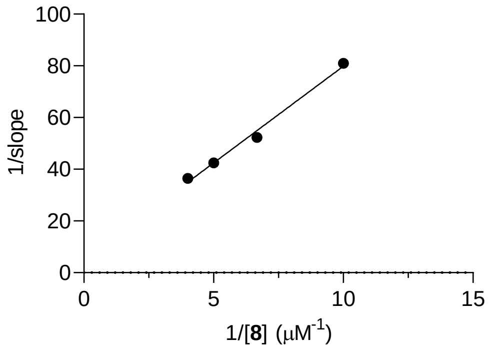
Time-dependent inhibition on MIF	Y = 7,476*X + 5,069
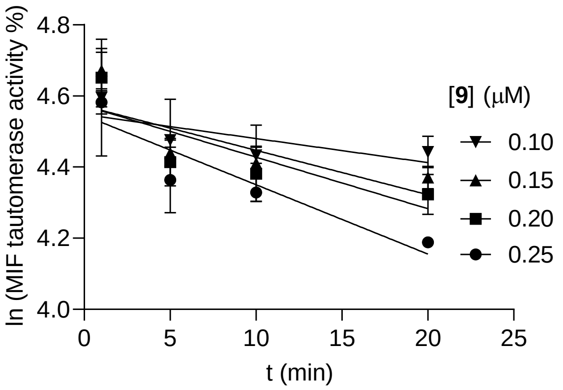	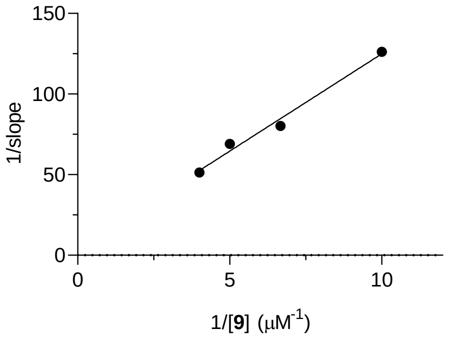
Time-dependent inhibition on MIF	Y = 12,07*X + 4,234
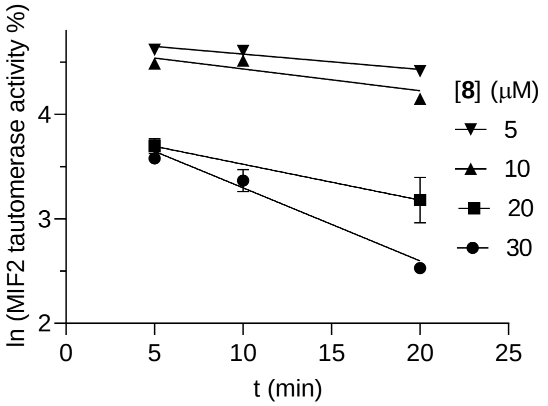	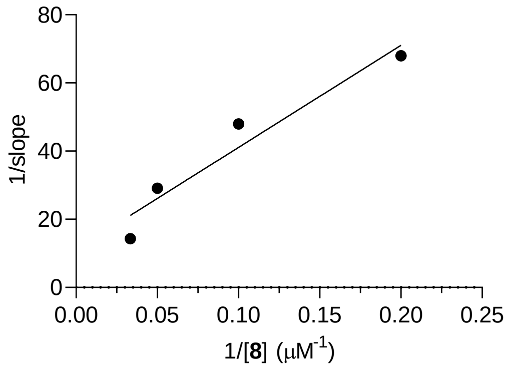
Time-dependent inhibition on MIF2	Y = 299,5*X + 11,14

<table>
<tr><td>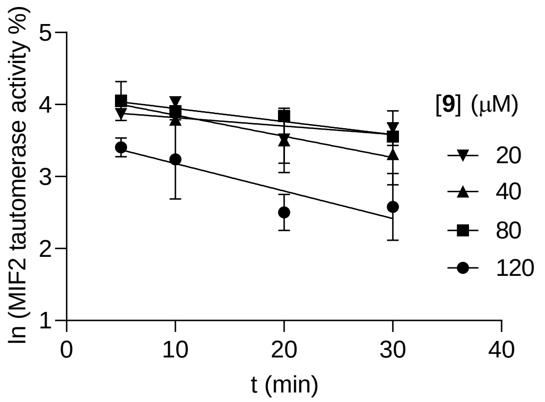
</td><td>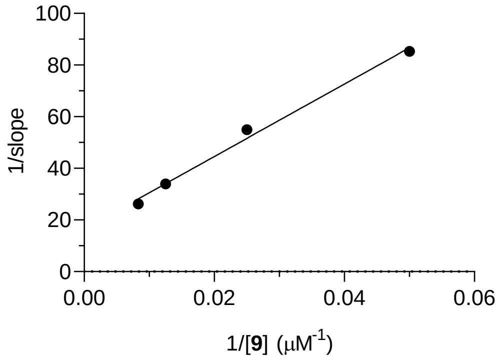
</td></tr>
<tr><td>Time-dependent inhibition on MIF2</td><td>Y = 1404*X + 16,49</td></tr>
<tr><td>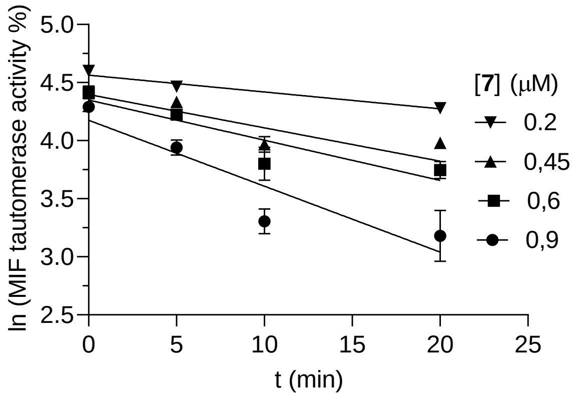
</td><td>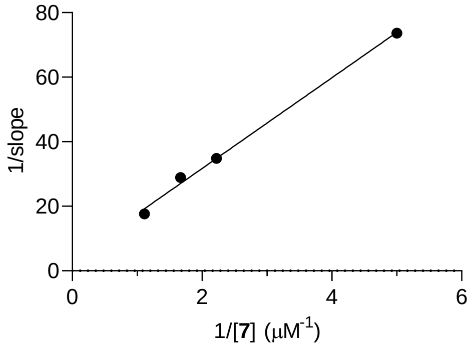
</td></tr>
<tr><td>Time-dependent inhibition on MIF</td><td>Y = 14,05*X + 3,602</td></tr>
</table>

4. Purified protein labeling and Mass spectra

4.1 MIF or MIF2 labeling with probe 8

MIF or MIF2 were diluted to desired concentration with PBS buffer and incubated with probe **8** at corresponding concentration and for corresponding time. Subsequently, the mixture was denatured (99 °C, 10 min). Labeled protein and free probe were separated by 12% Bis-Tris gel (Bio-Rad Laboratories, #4561084). Probe **8** labeled protein was visualized using in-gel fluorescence.

4.2 MIF or MIF2 labeling and mass spectra

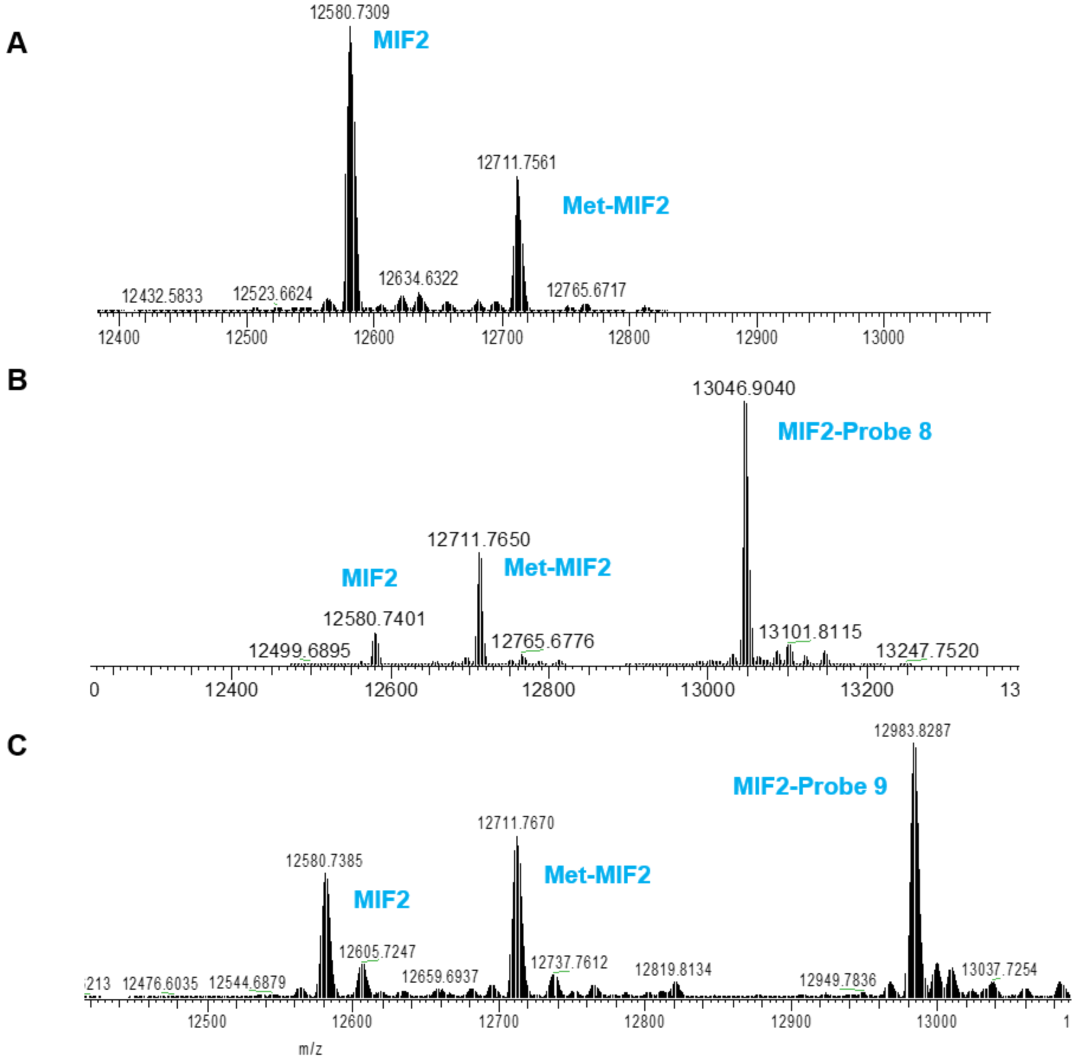

Figure S2. Mass spectra of labeled MIF2. (A) Native MIF2 and probe **8** (B) or probe **9** (C) labeled MIF2.

A

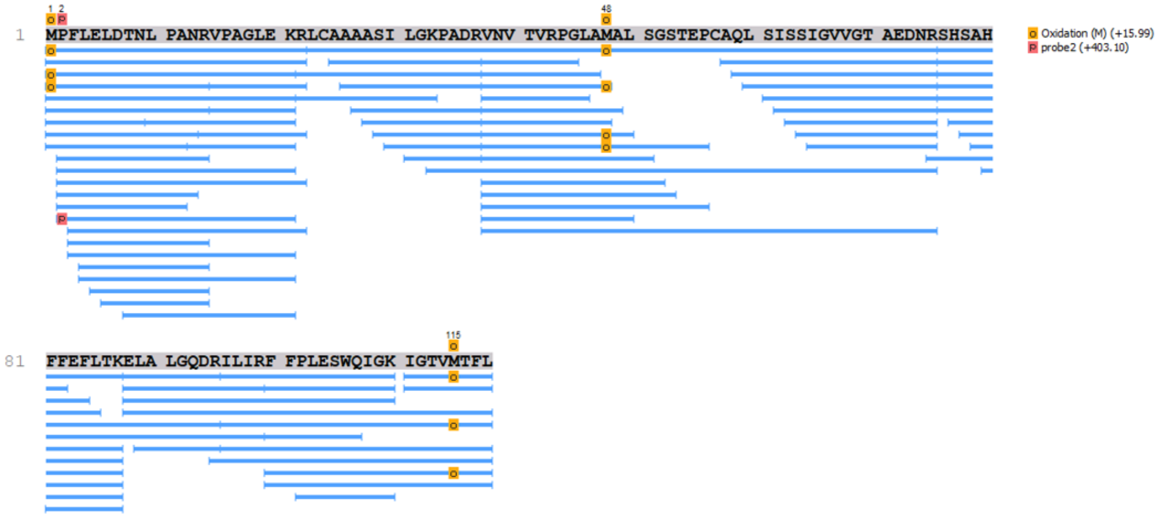

B

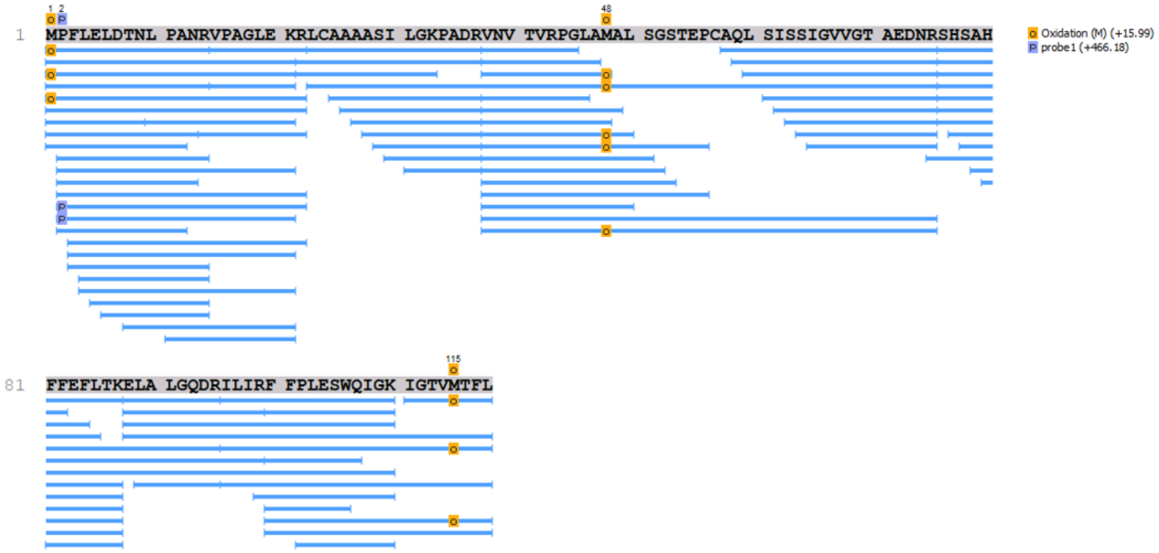

Figure S3. Labeled residue identification of MIF2. Digestion of (A) probe **8** (B) or probe **9** (C) labeled MIF2.

5. Cell lysate labeling

5.1 Cell culture and preparation of cell lysate.

Human A549 cells (ATCCCCL-185) or HeLa cells (ATCCCRL-1573) were cultured in RPMI 1640 Medium (GibcoTM #61870-010) containing 10% (v/v) fetal bovine serum (FBS), 100 U/mL penicillin/streptomycin (GibcoTM#10378016) at 37°C with 5% CO_2 in humidified air. Desired amount of cells were collected and lysed by RIPA buffer containing 1× protease inhibitor (PI) cocktail (Roche, Mannheim, Germany). The BCA Protein Assay Kit (Pierce, Rockford IL, USA) was used to determine the protein concentration.

5.2 Cell lysate labeling

The cell lysate (20-30 µg) was incubated with different concentration of probe 9 for different time. To detect the labeled proteome, the prepared samples were loaded onto a 12% Bis-Tris gel (Bio-Rad Laboratories, #4561084) and separated (120V, 120 minutes). Then, proteins were transferred to a polyvinylidene fluoride (PVDF) /PVDG membrane using a Bio-Rad Trans-Blot semi-dry blotter (300 mA, 90 min). Afterwards, nonspecific antibody binding was blocked with 5% non-fat dried milk in 0.1% PBST. The membrane was incubated overnight at 4 °C with 5% BSA in 0.1% PBST (5% PBSTB) with streptavidin-HRP. Afterwards, the membrane was treated with an ECL^{TM} Prime Western Blotting System (GE Healthcare #RPN2232). ChemiDoc MP Imaging Systems (Bio-RAD) was used to generate the immunoblot, which was subsequently visualized and analyzed using ImageJ software.

To detect the labeling of MIF or MIF2 in the cell lysate, an enrichment was conducted before the detection. The probe **9** treated samples were enriched using streptavidin-coated beads (ThermoFisher #88816) by following the protocol. The enriched proteins were tested with western-blot using anti-MIF (1:1000, ThermoFisher. #PA5-27343) or anti-MIF2 (1:1000, tebu-bio, #039600-401-R05) antibodies to detect MIF and MIF2.

To explore the possibility to preferentially label MIF2 using probe **9** after blocking MIF using an MIF the MIF selective inhibitor **7**. A 1:1 mixture (200 nM for each) of MIF and MIF2 was firstly incubated with desired concentration of **7** for 10 minutes at room temperature. Then 5 µM probe **9** was added into the mixture and incubated for one hour. The probe **9** labeled proteins were enriched and analyzed using methods mentioned above.

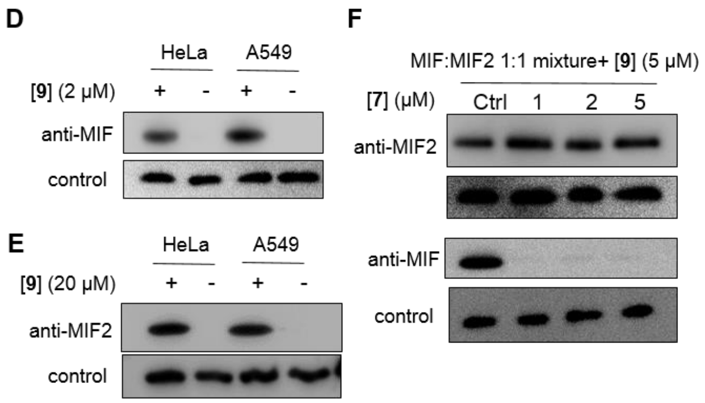

Figure S4. Internal references (controls) for Figures 3D, 3E, and 3F.

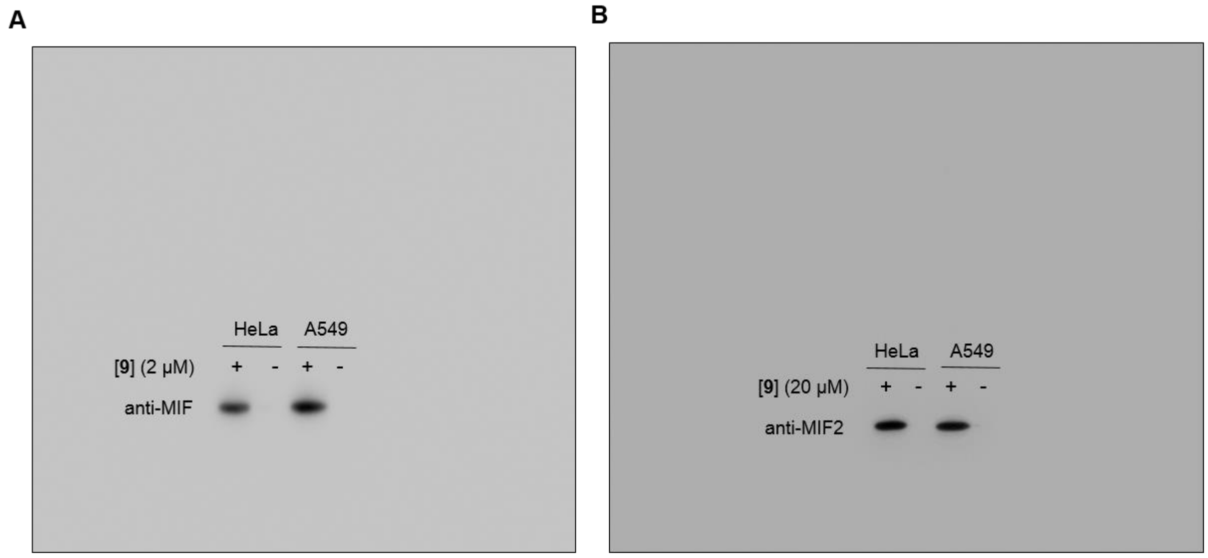

Figure S5. Uncropped western-blot image. (A) Uncropped image of Figure 3D; (B) Uncropped image of Figure 3E.

6. Subcellular localization of MIF and MIF2.

To visualize the MIF-oriented labeling, immunofluorescence staining was applied. HeLa cells were seeded on glass coverslips in a 6-well plate at a density of 1x10^5 cells per well. After overnight incubation at 37°C, cells were treated with 10 µM probe **8** for 2 hour. The cells were fixed with methanol and then unspecific binding was blocked with blocking buffer (1% BSA, 22.52 mg/mL glycine in 0.1%PBST). Cells were incubated with a MIF antibody (1:500, ThermoFisher, #PA5-27343) for 1 hour at room temperature. Subsequently, cells were treated with Alexa Fluor Plus 488 conjugated goat anti-rabbit secondary antibody (1:1000, #A32732, Invitrogen, Waltham, MA. USA). Next, coverslips were mounted onto slides with anti-fading mounting medium with DAPI stain (Invitrogen, Waltham, MA. USA). The pictures were acquired using a Leica SP8 confocal laser scanning microscope and analyzed by ImageJ.

To visualize the MIF2-oriented labeling, immunofluorescence staining was applied. HeLa cells were seeded on glass coverslips in a 6-well plate at a density of 1x10^5 cells per well. After overnight incubation at 37°C, cells were treated with 10 µM compound **7** for half hour. After removal of 7, cells were treated with 50 µM probe **8** for 3 hour. The cells were fixed with methanol and then unspecific binding was blocked with blocking buffer (1% BSA, 22.52 mg/mL glycine in 0.1%PBST). Cells were incubated with a MIF2 antibody (1:500, ThermoFisher, #PA5-27343) for 1 hour at room temperature. Subsequently, cells were treated with Alexa Fluor Plus 488 conjugated goat anti-rabbit secondary antibody (1:1000, #A32732, Invitrogen, Waltham, MA. USA). Next, coverslips were mounted onto slides with anti-fading mounting medium with DAPI stain (Invitrogen, Waltham, MA. USA). The pictures were acquired using a Leica SP8 confocal laser scanning microscope and analyzed by ImageJ.

To stimulate the apoptosis of cells, the HeLa cells were treated with 50 µM MNNG for 15 min before labeling with probe **8**.[8]

To provide further proof that probe **8** has value for MIF- or MIF2-directe labelling an additional fluorescence microscopy experiment was performed. In this experiment the labelling with probe **8** was outcompeted with a competitive inhibitor that is structurally distinct from probe **8**. A potent MIF-selective inhibitor containing 7-hydroxy-3,4-dihydrobenzoxazin-2-ones backbone with an IC_{50} of 150 nM from our previous publication was utilized.[9] The same type of experiment was performed for MIF2 in which we applied a MIF2-selective inhibitor with a potency of IC_{50}=1 µM (unpublished research data). Co-incubation with either the MIF- or MIF2 inhibitor with the respective MIF- or MIF2 staining method abolished the staining pattern

observed in absence of the inhibito (Figure S6A and S6B). More importantly, whereas the MIF-directed labelling with probe 8 overlaps with the antibody based MIF labelling, coincubation of probe **8** with the MIF inhibitor (50 μM) provided a labelling pattern that differs clearly from the MIF antibody-based labelling (Figure R2A). The same is observed for the MIF2-directed labelling in presence of the MIF2 inhibitor. In the presence of the MIF2 inhibitor (100 μM). The unique nuclear membrane localization of MIF2 staining that was observed with both the probe **8** MIF2-directed staining and the antibody based staining was diminished. These observations indicate that both the MIF and MIF2 directed staining are indeed relevant for MIF and MIF2.

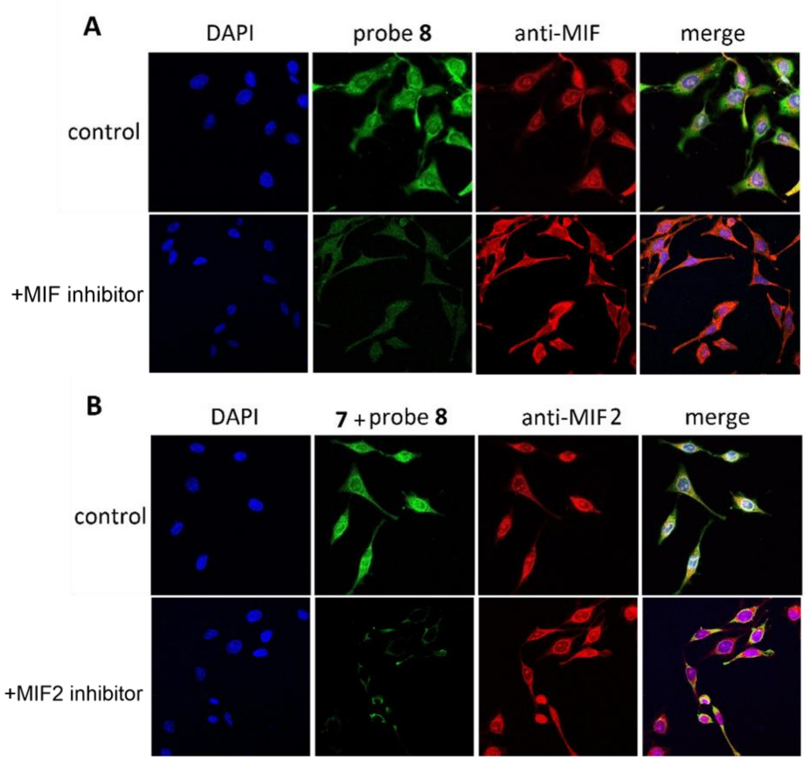

Figure S6. MIF or MIF2 tautomerase activity inhibitor changes the labelling pattern of probe **8**. In the inhibitors treated groups, cell was pretreated with (A) MIF inhibitor or (B) MIF2 inhibitor before labelling with probe 8, respectively.

7. Nuclease activity of MIF and MIF2.

Human genomic DNA was obtained from RPE-1 cells by DNA extraction following the protocol described in literature.[10] The nuclease assays were conducted by following the procedure described previously.[8] hgDNA (200 ng/reaction) was incubated with MIF or MIF2 in final concentration of 0.62-20 μM as indicated in 10Mm Tris-HCl buffer (pH 7.0) containing 10mM $MgCl_2$ (B and C) or specific buffer as indicated at 37°C for two hours. The reaction was terminated with loading buffer containing 10mM EDTA and incubation on ice. The human genomic DNA samples were immediately separated on a 1% agarose gel containing SYBR Safe (ThermoFisher) in 0.5 X TBE buffer with initial switch time of 1.5 s and a final switch time of 3.5 s for 12 hours at 6 V/cm. DNA was further detected by chemiluminescence.

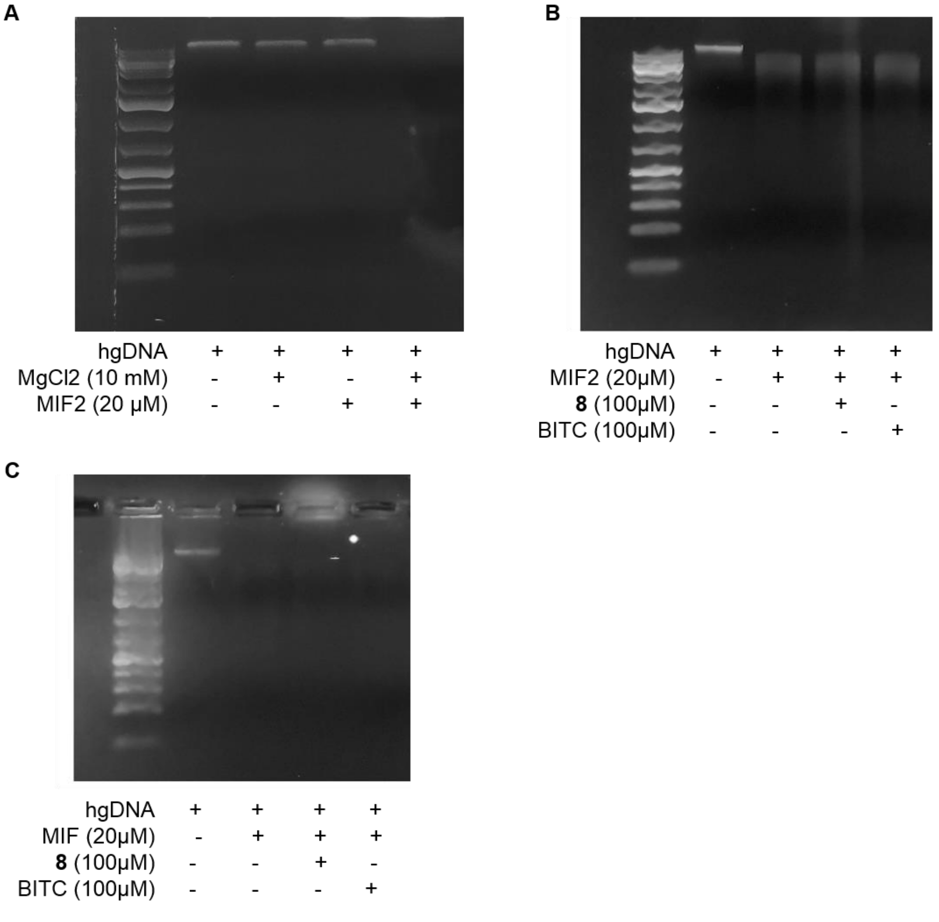

Figure S7. hgDNA cleavage activity of MIF and MIF2. (A) The MIF2 nuclease activity is Mg^{2+}-dependent; (B) Tautomerase inhibitors showed no influence on the nuclease activity of MIF2. BITC is a covalent inhibitor that can form covalent bond with the N-terminal proline.[11] (C) Tautomerase inhibitors showed no influence on the nuclease activity of MIF.

8. References

[1] Z. Xiao, D. Chen, S. Song, R. van der Vlag, P. van der Wouden, R. van Merkerk, R. Cool, A. Hirsch, B. Melgert, W. J. Quax, G. Poelarends, F. Dekker, *J. Med. Chem.* **2020**, *63*, 11920–11933.

[2] C. A. Lefebvre, E. Forcellini, S. Boutin, M. F. Côté, R. C.-Gaudreault, P. Mathieu, P. Lagüe, J. F. Paquin, *Bioorg. Med. Chem. Lett.* **2017**, *27*, 299–302.

[3] H. L. Wang, J. Katon, C. Balan, A. W. Bannon, C. Bernard, E. M. Doherty, C. Dominguez, N. R. Gavva, V. Gore, V. Ma, N. Nishimura, S. Surapaneni, P. Tang, R. Tamir, O. Thiel, J. J. S. Treanor, M. H. Norman, *J. Med. Chem.* **2007**, *50*, 3528–3539.

[4] T. Kok, A. A. Wasiel, F. J. Dekker, G. J. Poelarends, R. H. Cool, *Protein Expr. Purif.* **2018**, *148*, 46–53.

[5] Z. Xiao, M. Fokkens, D. Chen, T. Kok, G. Proietti, R. van Merkerk, G. J. Poelarends, F. J. Dekker, *Eur. J. Med. Chem.* **2020**, *186*, 111849–111862.

[6] J. Bernhagen, R. A. Mitchell, T. Calandra, W. Voelter, A. Cerami, R. Bucala, *Biochemistry* **1994**, *33*, 14144–14155.

[7] S. Song, B. Liu, H. Habibie, J. van den Bor, M. J. Smit, R. Gosens, X. Wu, C.-A. Brandsma, R. H. Cool, H. J. Haisma, G. J. Poelarends, B. N. Melgert, *EBioMedicine* **2021**, *68*, 103412.

[8] Y. Wang, R. An, G. K. Umanah, H. Park, K. Nambiar, S. M. Eacker, B. Kim, L. Bao, M. M. Harraz, C. Chang, R. Chen, J. E. Wang, T. I. Kam, J. S. Jeong, Z. Xie, S. Neifert, J. Qian, S. A. Andrabi, S. Blackshaw, H. Zhu, H. Song, G. L. Ming, V. L. Dawson, T. M. Dawson, *Science.* **2016**, *354*, 82–96.

[9] Z. Xiao, D. Chen, S. Song, R. Van Der Vlag, P. E. Van Der Wouden, R. Van Merkerk, R. H. Cool, A. K. H. Hirsch, B. N. Melgert, W. J. Quax, G. J. Poelarends, F. J. Dekker, *J. Med. Chem.* **2020**, *63*, 11920–11933.

[10] S. Kit Leng Lui, S. Keegan, P. Tonzi, M. Kahli, Y. H. Chen, N. Chalhoub, K. E. Coleman, D. Fenyo, D. J. Smith, T. T. Huang, *Nat. Protoc.* **2021**, *16*, 1193–1218.

[11] H. Ouertatani-Sakouhi, F. El-Turk, B. Fauvet, T. Roger, D. Le Roy, D. P. Karpinar, L. Leng, R. Bucala, M. Zweckstetter, T. Calandra, H. A. Lashuel, *Biochemistry* **2009**, *48*, 9858–9870.

9. NMR and Mass spectra.

1a, FM002

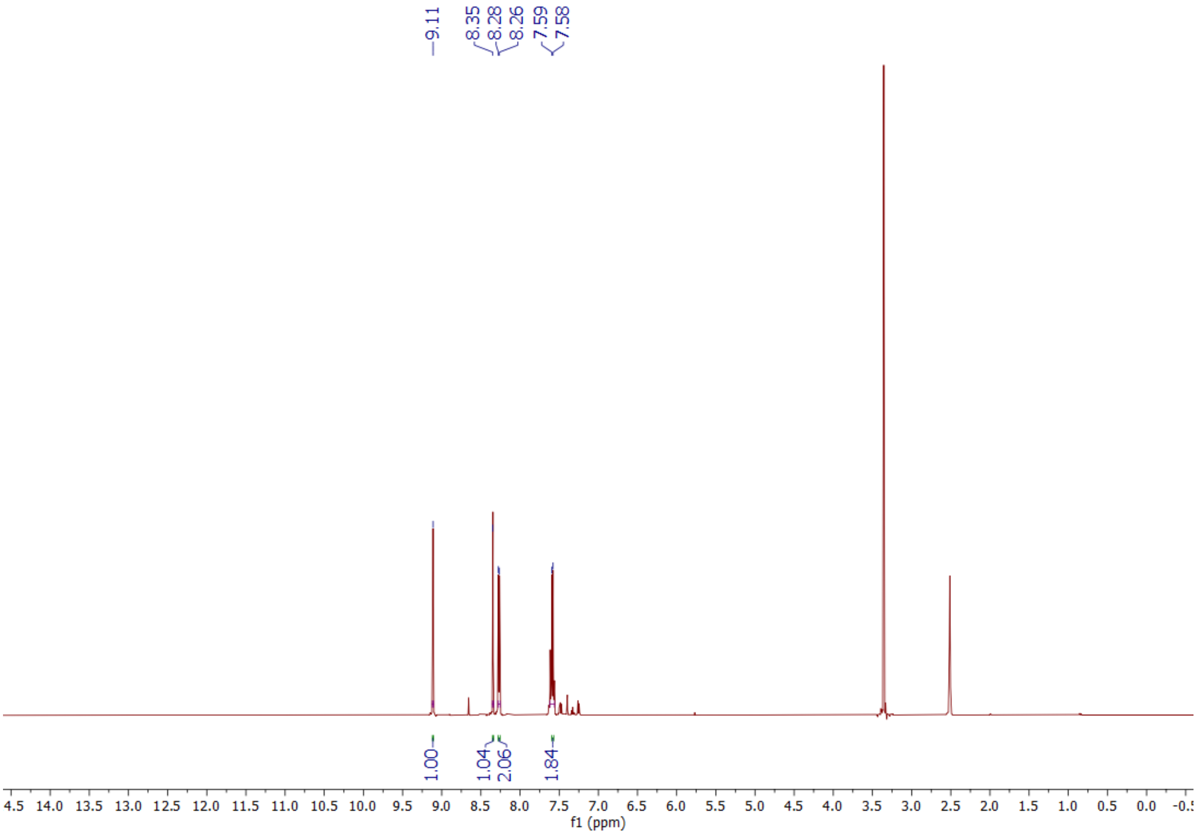

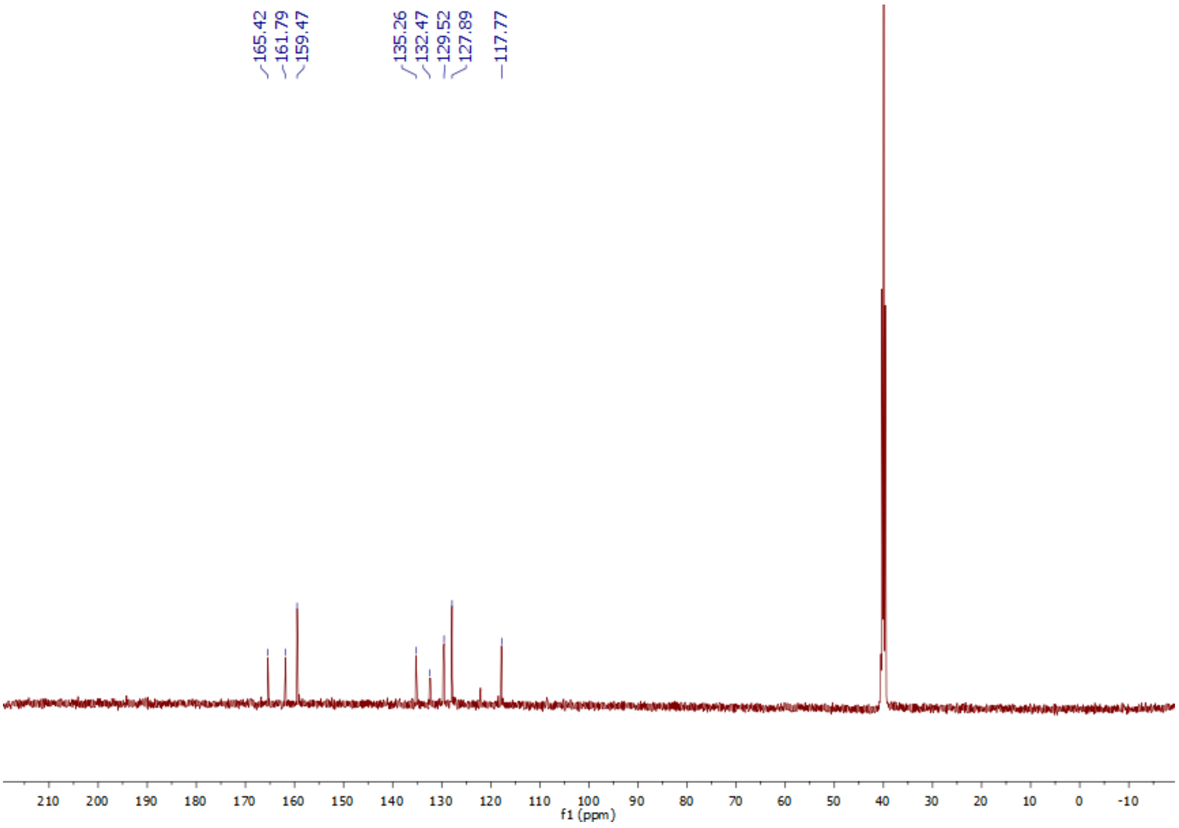

1, 4-IPP, FM004

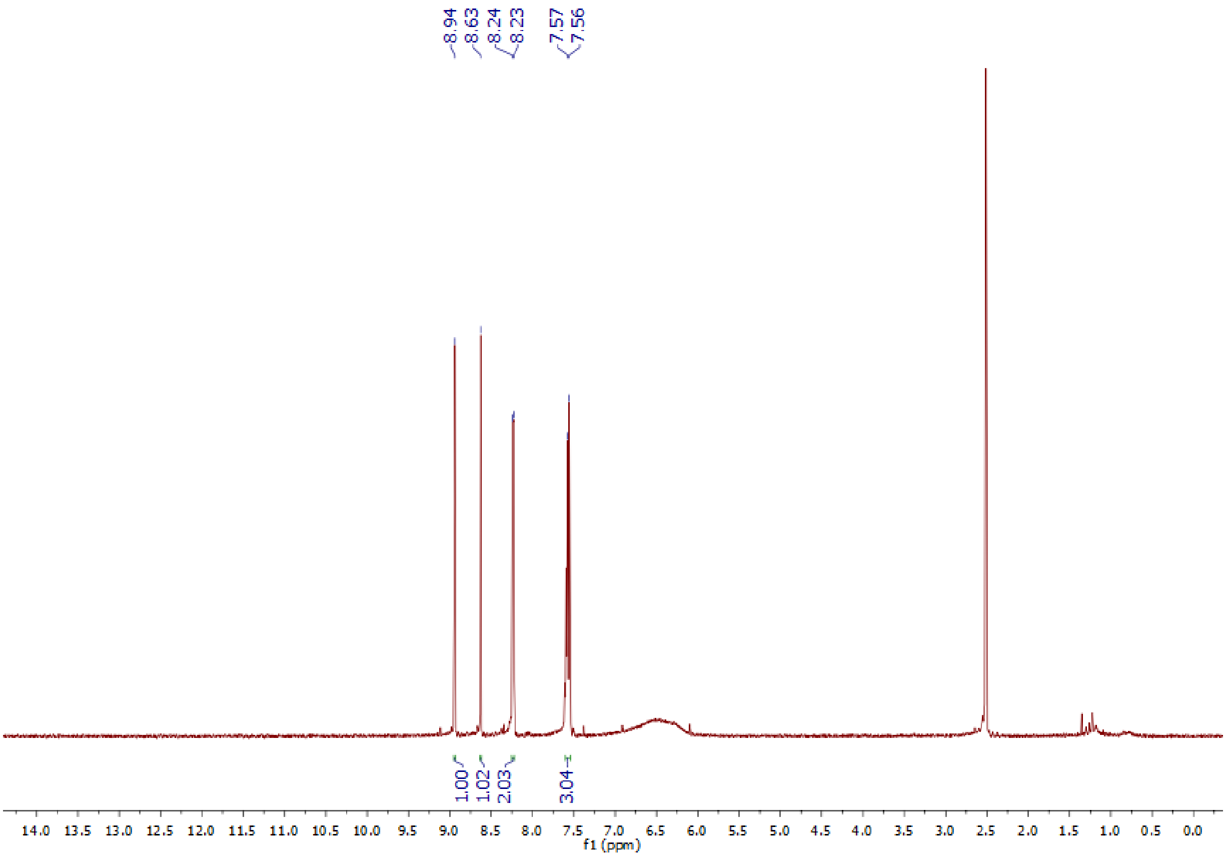

8.94
8.63
8.24
8.23
7.57
7.56
1.00
1.02
2.03
3.04
14.0 13.5 13.0 12.5 12.0 11.5 11.0 10.5 10.0 9.5 9.0 8.5 8.0 7.5 7.0 6.5 6.0 5.5 5.0 4.5 4.0 3.5 3.0 2.5 2.0 1.5 1.0 0.5 0.0
f1 (ppm)

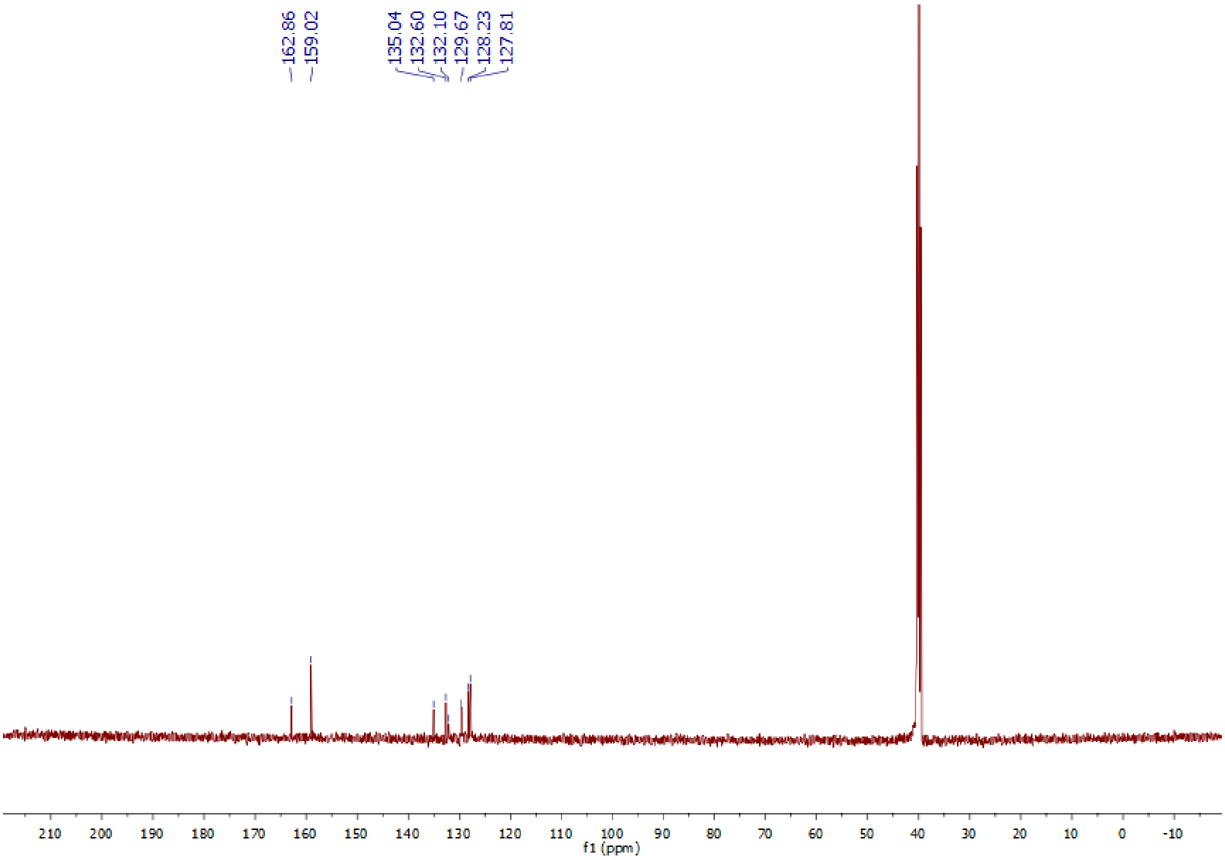

162.86
159.02
135.04
132.60
132.10
129.67
128.23
127.81
210 200 190 180 170 160 150 140 130 120 110 100 90 80 70 60 50 40 30 20 10 0 -10
f1 (ppm)

2a, FM001

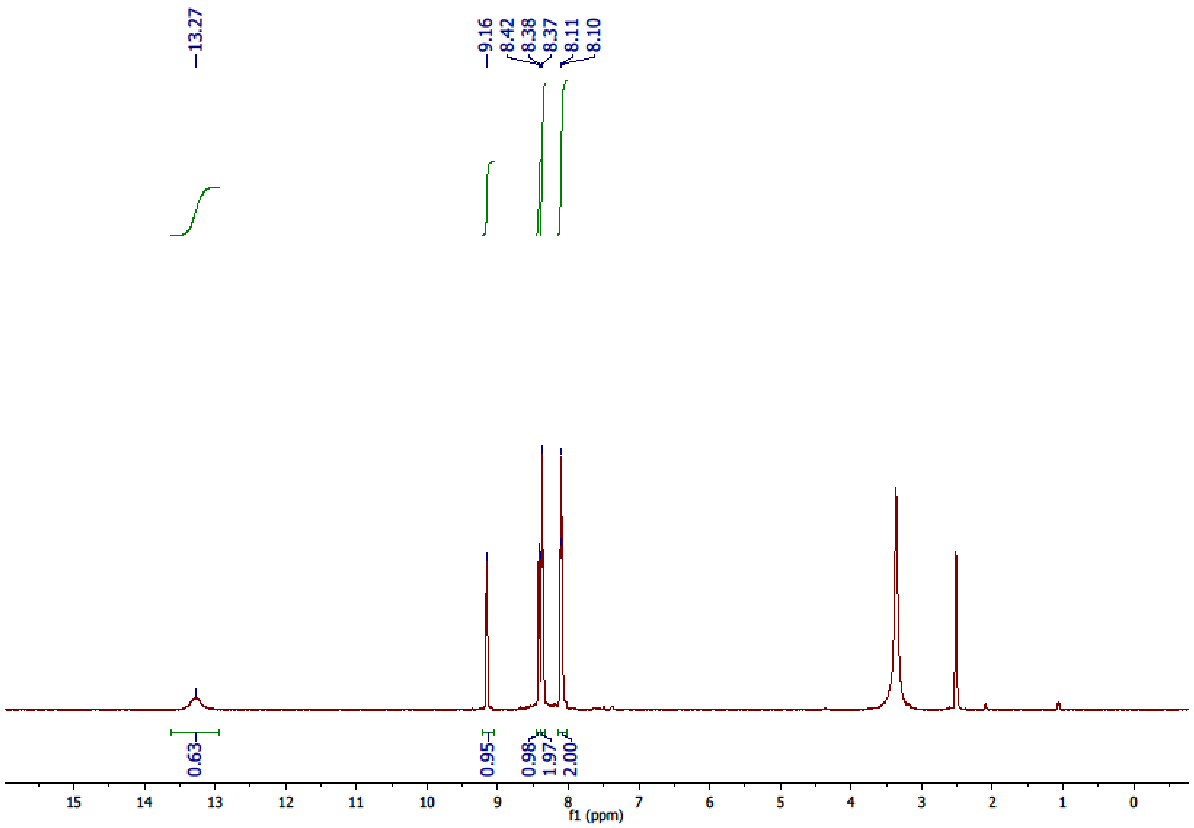

13.27
9.16
8.42
8.38
8.37
8.11
8.10
0.63
0.95
0.98
1.97
2.00
f1 (ppm)

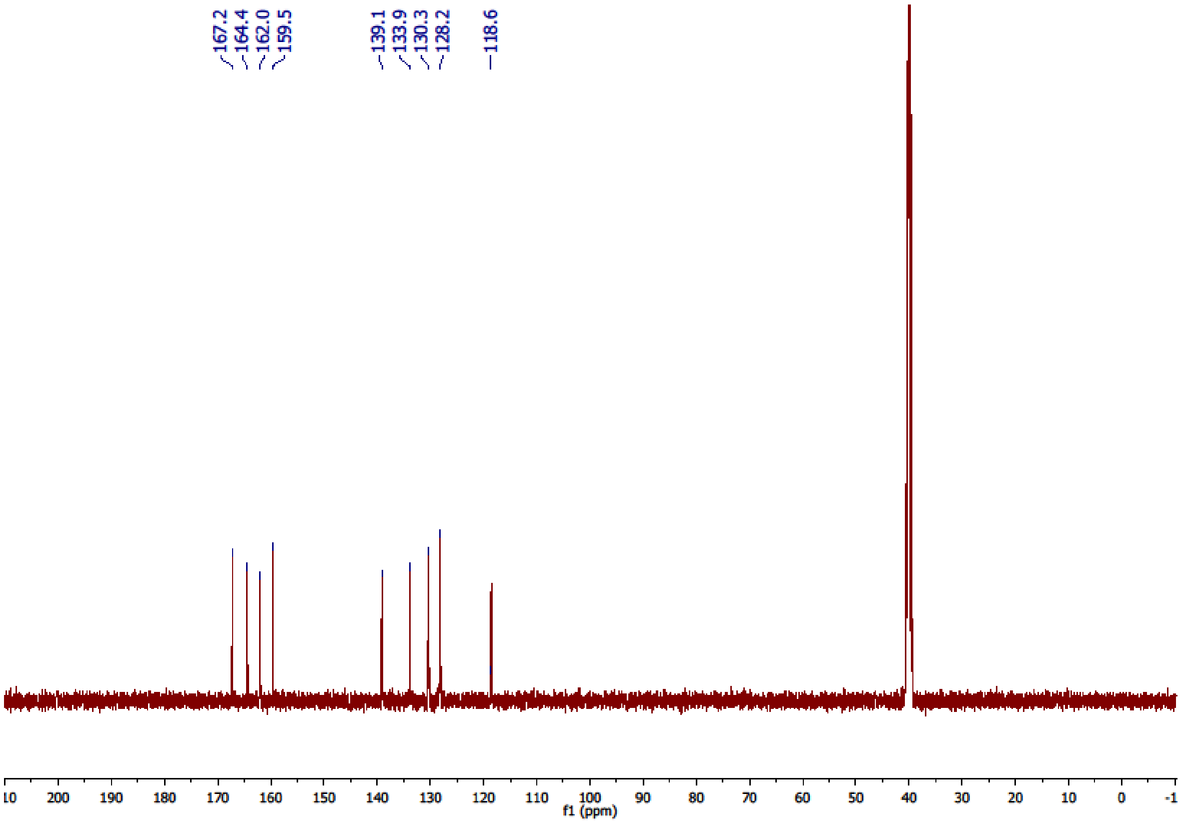

167.2
164.4
162.0
159.5
139.1
133.9
130.3
128.2
118.6
f1 (ppm)

2, FM003

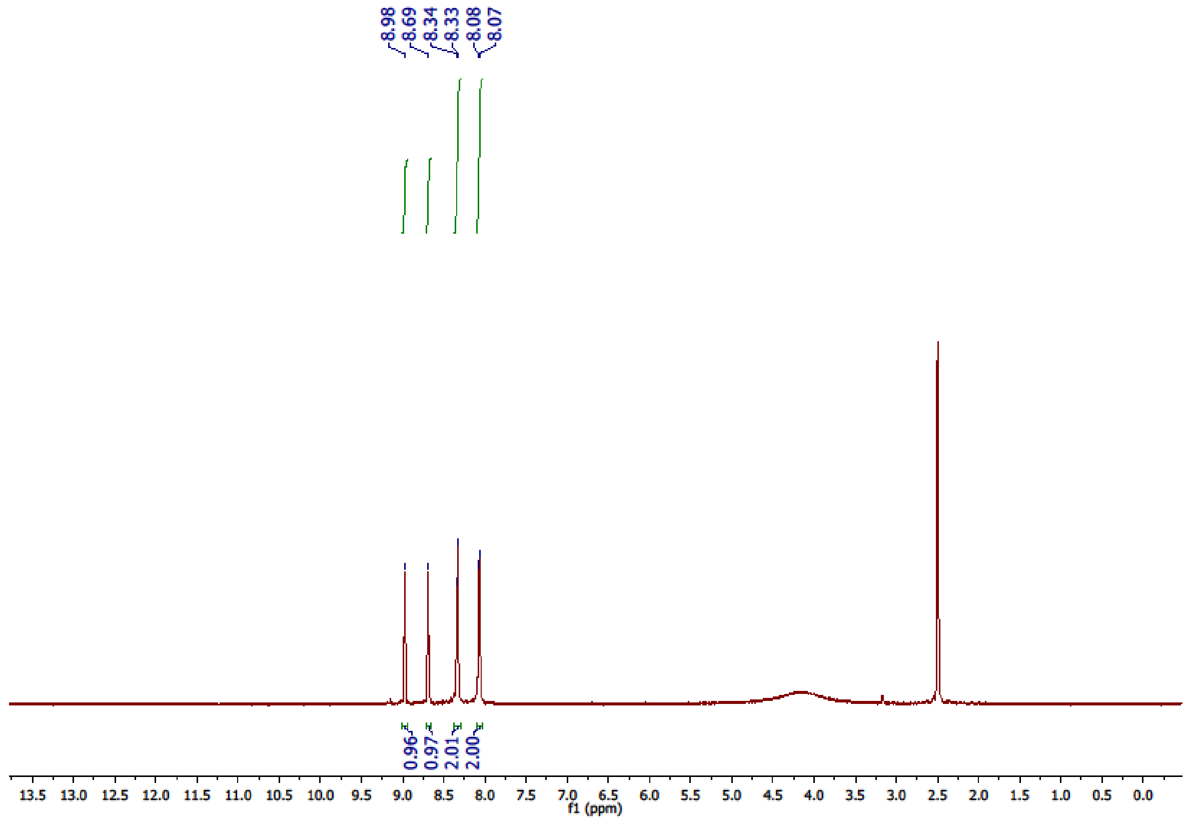

8.98
8.69
8.34
8.33
8.08
8.07
0.96
0.97
2.01
2.00
13.5 13.0 12.5 12.0 11.5 11.0 10.5 10.0 9.5 9.0 8.5 8.0 7.5 7.0 6.5 6.0 5.5 5.0 4.5 4.0 3.5 3.0 2.5 2.0 1.5 1.0 0.5 0.0
f1 (ppm)

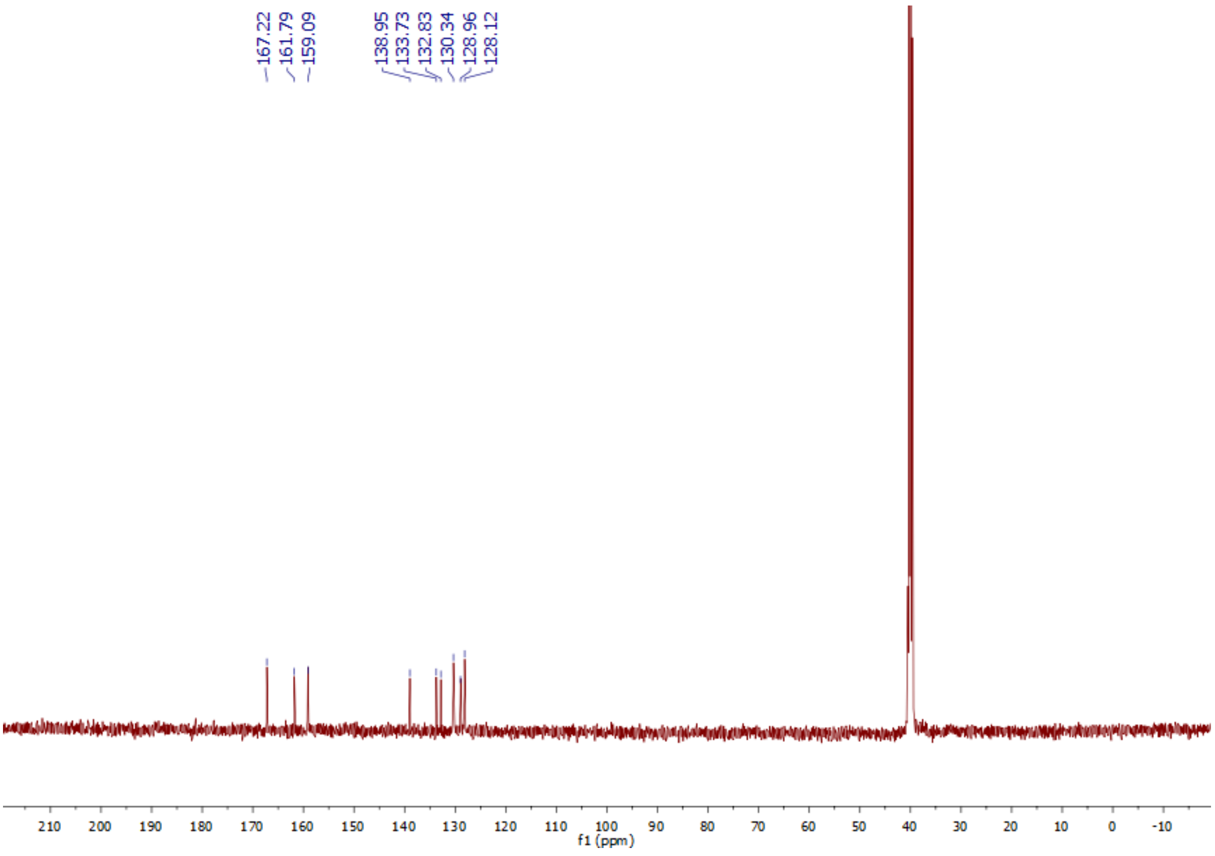

167.22
161.79
159.09
138.95
133.73
132.83
130.34
128.96
128.12
210 200 190 180 170 160 150 140 130 120 110 100 90 80 70 60 50 40 30 20 10 0 -10
f1 (ppm)

3a, FM005

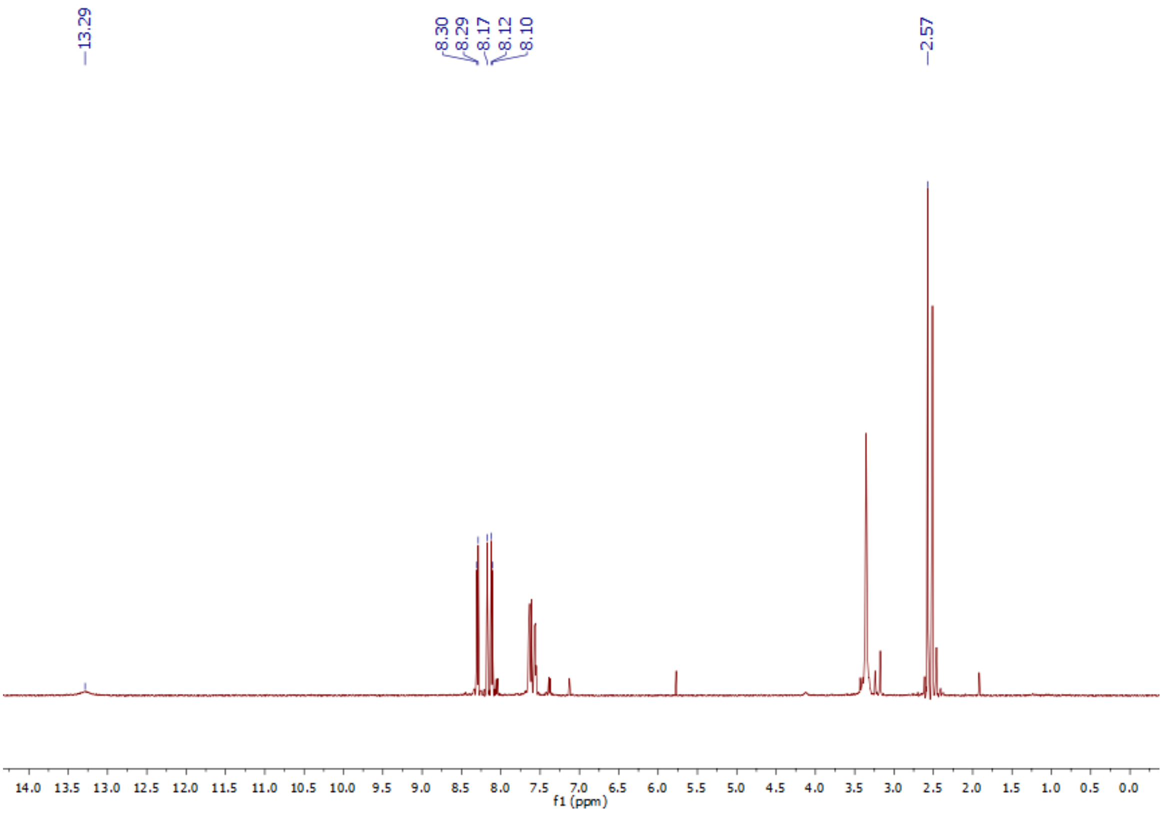

13.29
8.30
8.29
8.17
8.12
8.10
2.57
14.0 13.5 13.0 12.5 12.0 11.5 11.0 10.5 10.0 9.5 9.0 8.5 8.0 7.5 7.0 6.5 6.0 5.5 5.0 4.5 4.0 3.5 3.0 2.5 2.0 1.5 1.0 0.5 0.0
f1 (ppm)

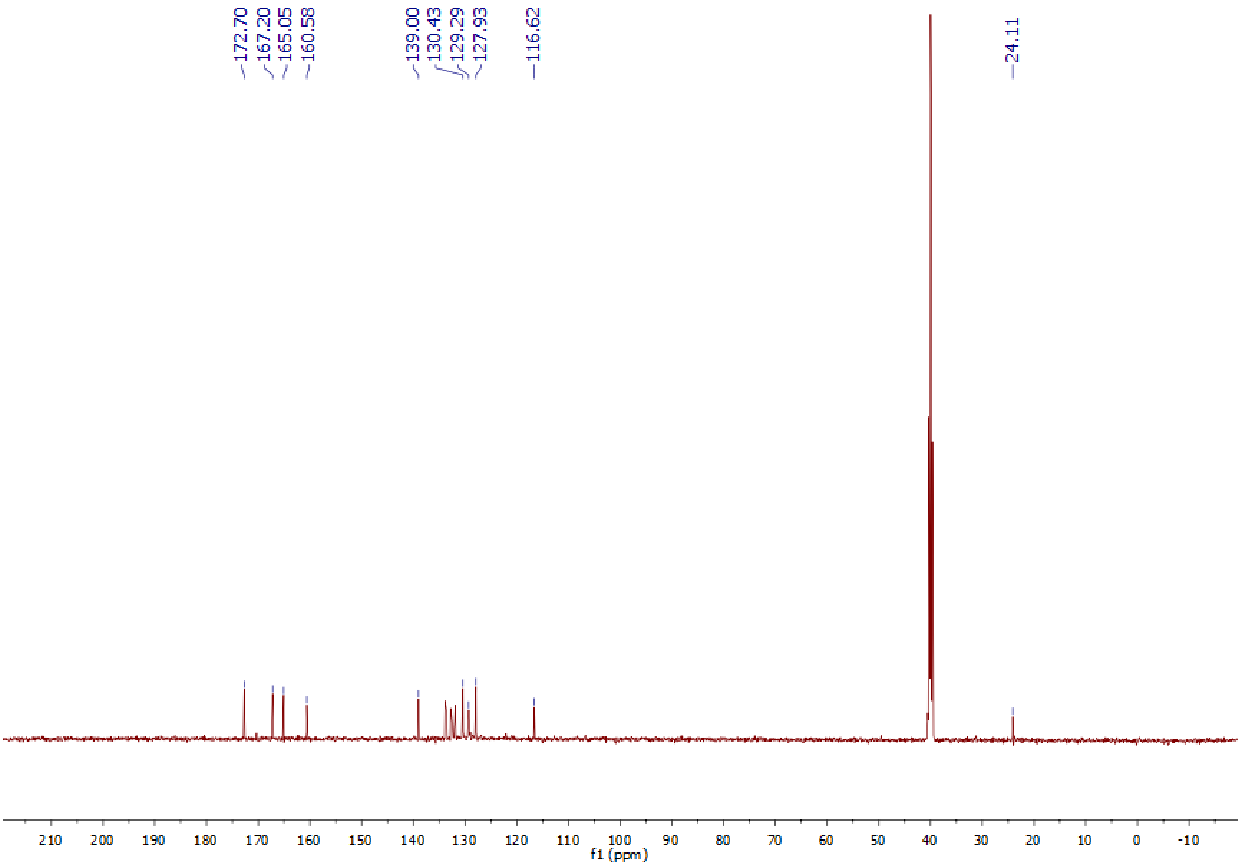

172.70
167.20
165.05
160.58
139.00
130.43
129.29
127.93
116.62
24.11
210 200 190 180 170 160 150 140 130 120 110 100 90 80 70 60 50 40 30 20 10 0 -10
f1 (ppm)

3, FM006

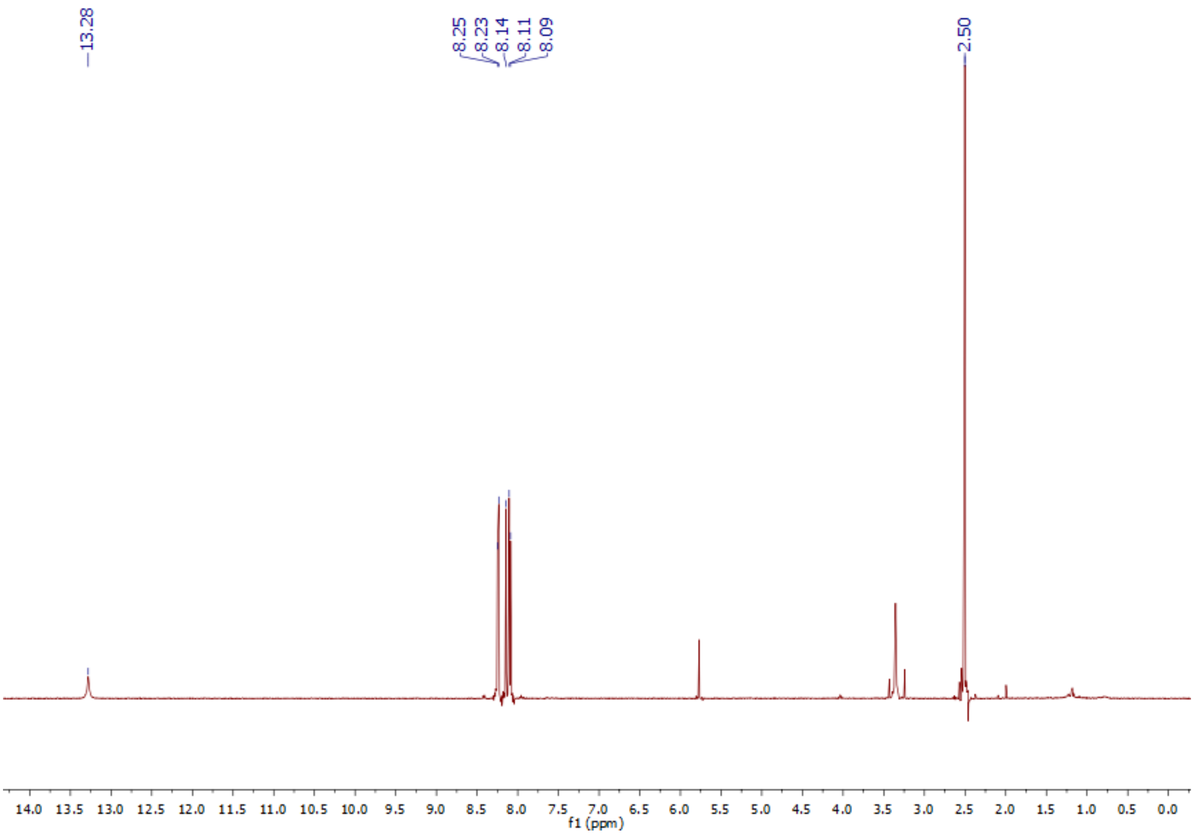

13.28
8.25
8.23
8.14
8.11
8.09
2.50
14.0 13.5 13.0 12.5 12.0 11.5 11.0 10.5 10.0 9.5 9.0 8.5 8.0 7.5 7.0 6.5 6.0 5.5 5.0 4.5 4.0 3.5 3.0 2.5 2.0 1.5 1.0 0.5 0.0
f1 (ppm)

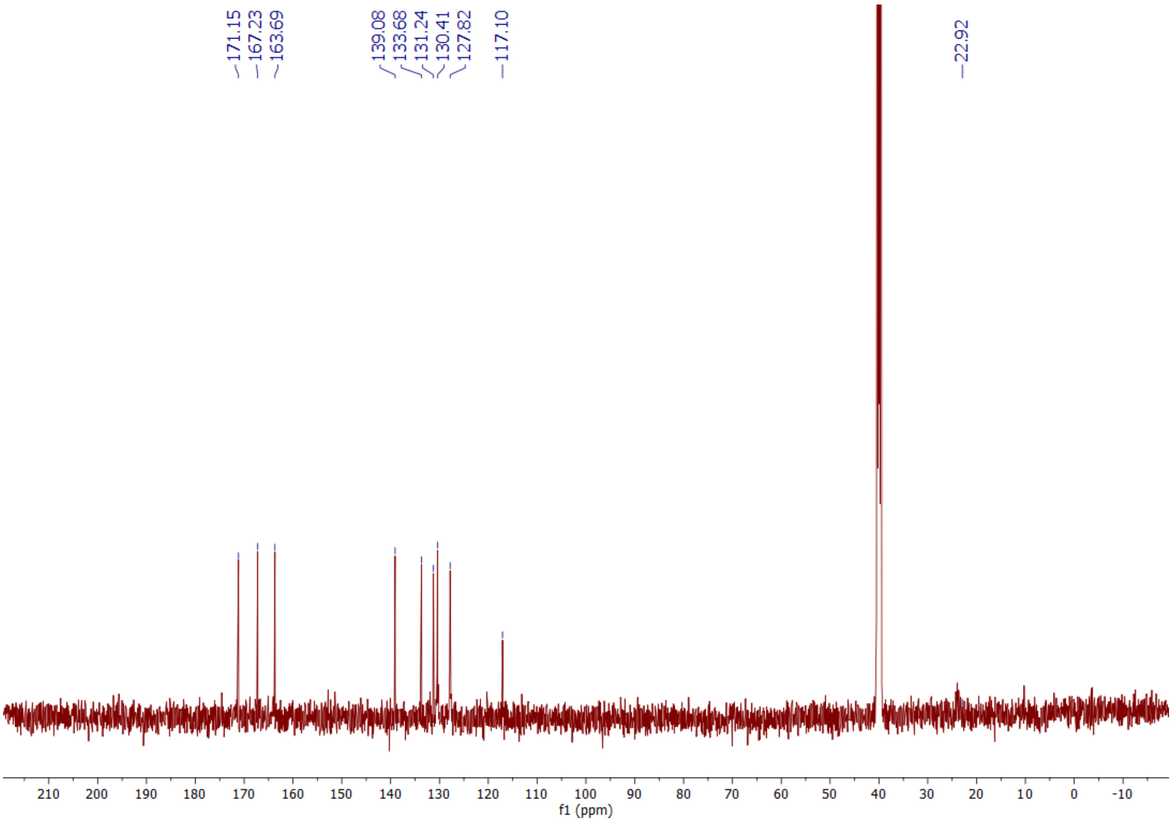

171.15
167.23
163.69
139.08
133.68
131.24
130.41
127.82
117.10
22.92
210 200 190 180 170 160 150 140 130 120 110 100 90 80 70 60 50 40 30 20 10 0 -10
f1 (ppm)

4a, FM009

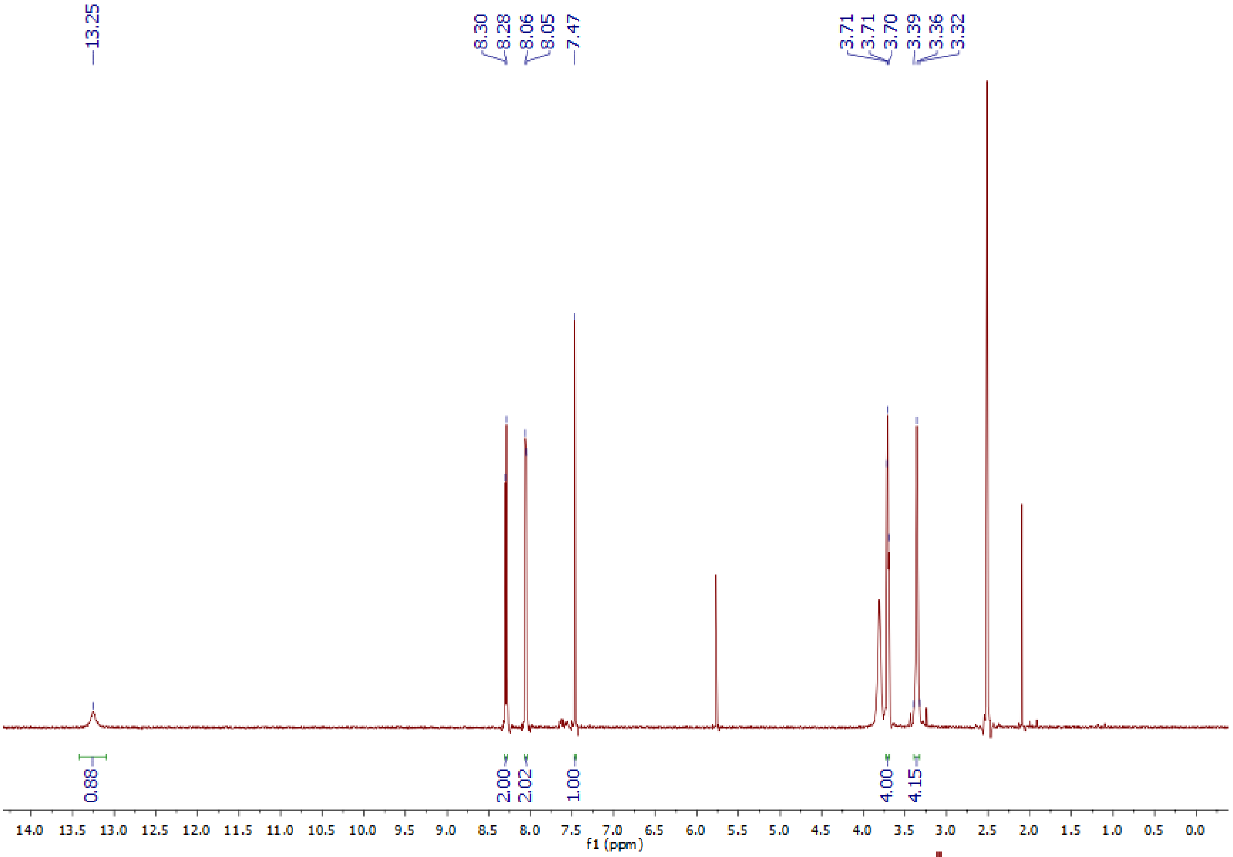
13.25
8.30
8.28
8.06
8.05
7.47
3.71
3.71
3.70
3.39
3.36
3.32
0.88
2.00
2.02
1.00
4.00
4.15
14.0 13.5 13.0 12.5 12.0 11.5 11.0 10.5 10.0 9.5 9.0 8.5 8.0 7.5 7.0 6.5 6.0 5.5 5.0 4.5 4.0 3.5 3.0 2.5 2.0 1.5 1.0 0.5 0.0
f1 (ppm)

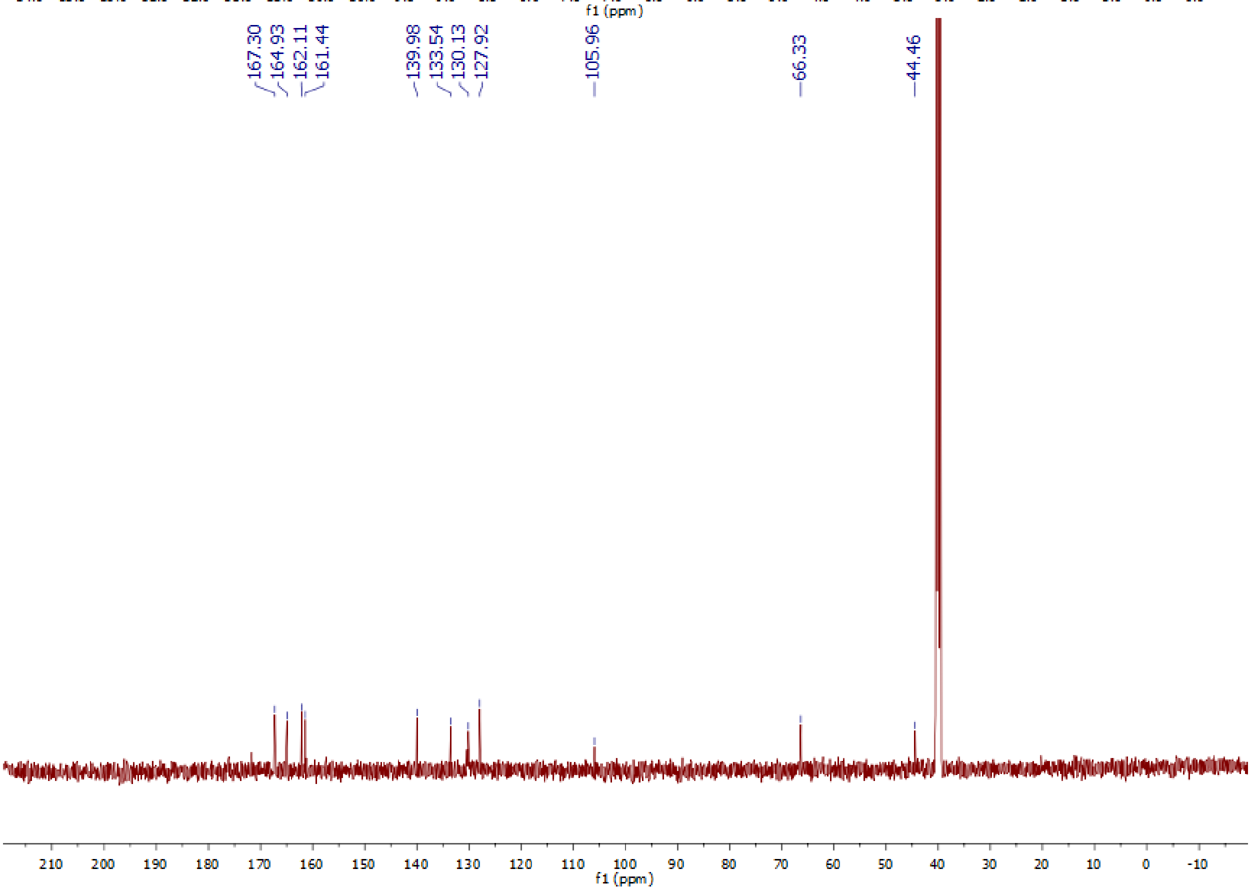
167.30
164.93
162.11
161.44
139.98
133.54
130.13
127.92
105.96
66.33
44.46
210 200 190 180 170 160 150 140 130 120 110 100 90 80 70 60 50 40 30 20 10 0 -10
f1 (ppm)

4, FM010

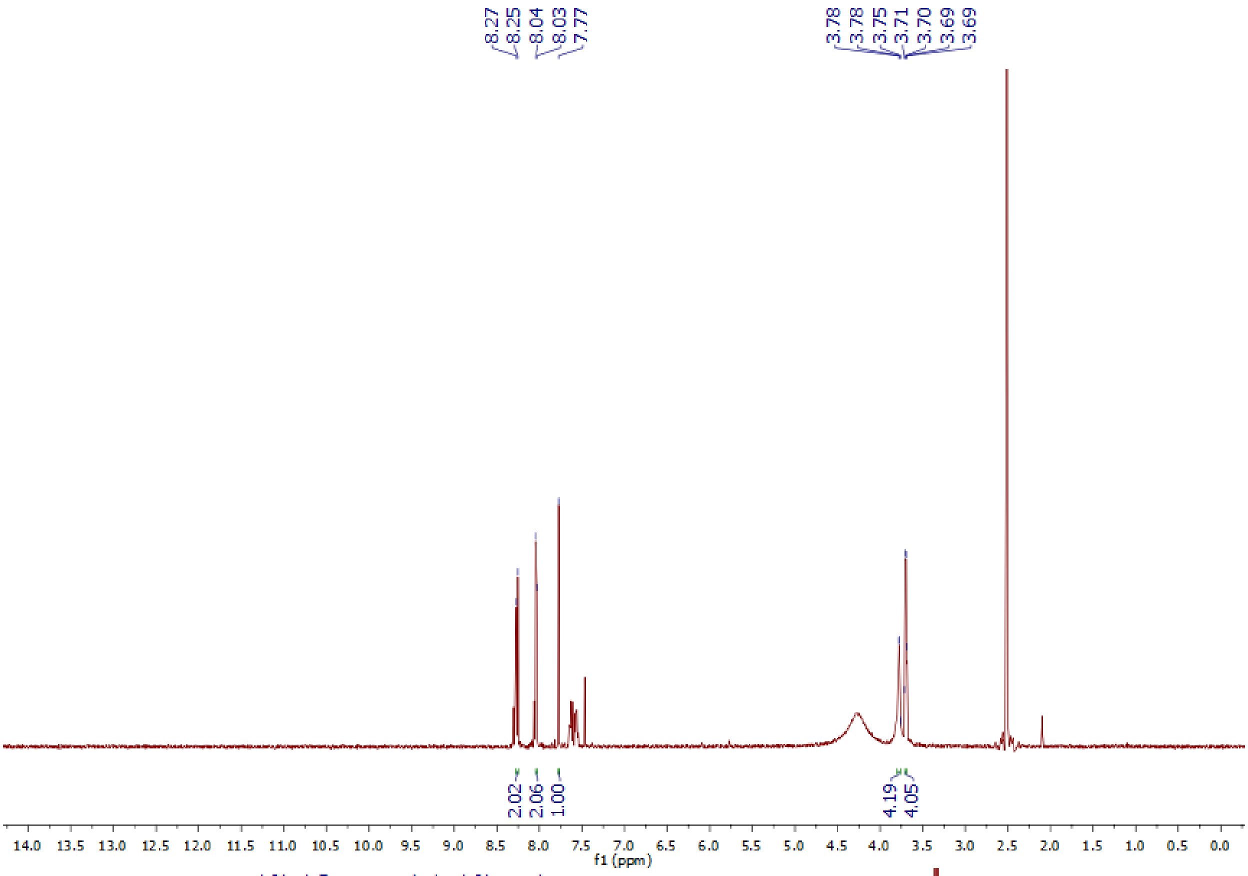
8.27
8.25
8.04
8.03
7.77
3.78
3.78
3.75
3.71
3.70
3.69
3.69
2.02
2.06
1.00
4.19
4.05
14.0 13.5 13.0 12.5 12.0 11.5 11.0 10.5 10.0 9.5 9.0 8.5 8.0 7.5 7.0 6.5 6.0 5.5 5.0 4.5 4.0 3.5 3.0 2.5 2.0 1.5 1.0 0.5 0.0
f1 (ppm)

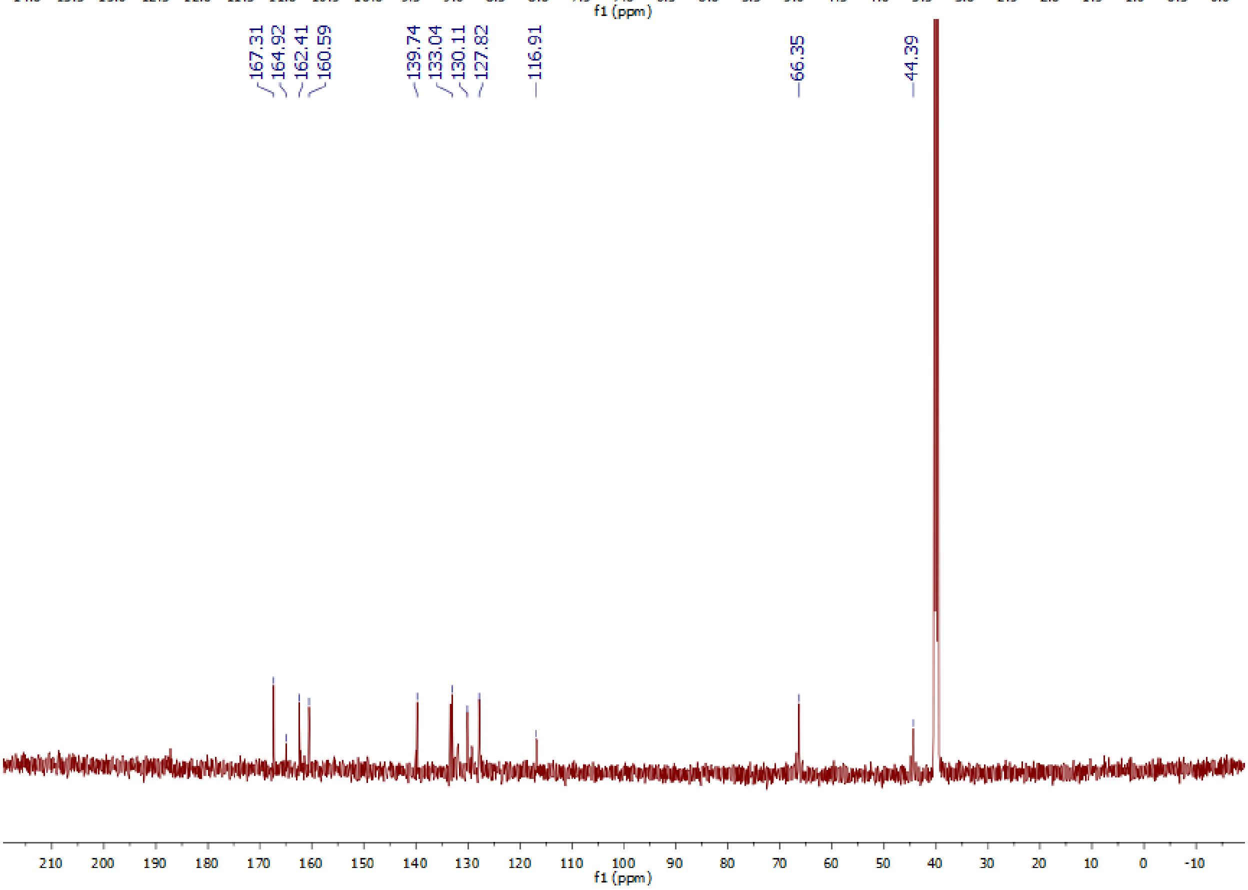
167.31
164.92
162.41
160.59
139.74
133.04
130.11
127.82
116.91
66.35
44.39
210 200 190 180 170 160 150 140 130 120 110 100 90 80 70 60 50 40 30 20 10 0 -10
f1 (ppm)

5a, FM011

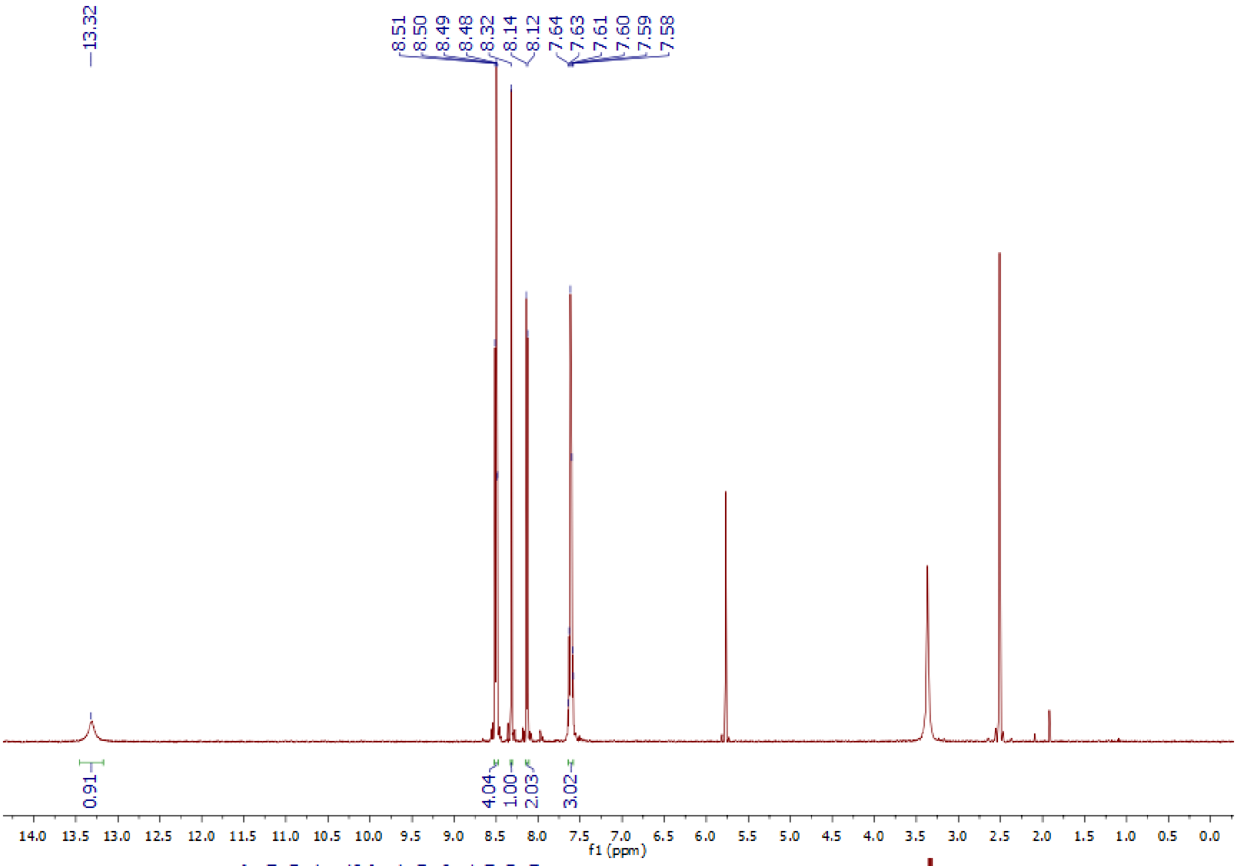

13.32
8.51
8.50
8.49
8.48
8.32
8.14
8.12
7.64
7.63
7.61
7.60
7.59
7.58
0.91
4.04
1.00
2.03
3.02
14.0 13.5 13.0 12.5 12.0 11.5 11.0 10.5 10.0 9.5 9.0 8.5 8.0 7.5 7.0 6.5 6.0 5.5 5.0 4.5 4.0 3.5 3.0 2.5 2.0 1.5 1.0 0.5 0.0
f1 (ppm)

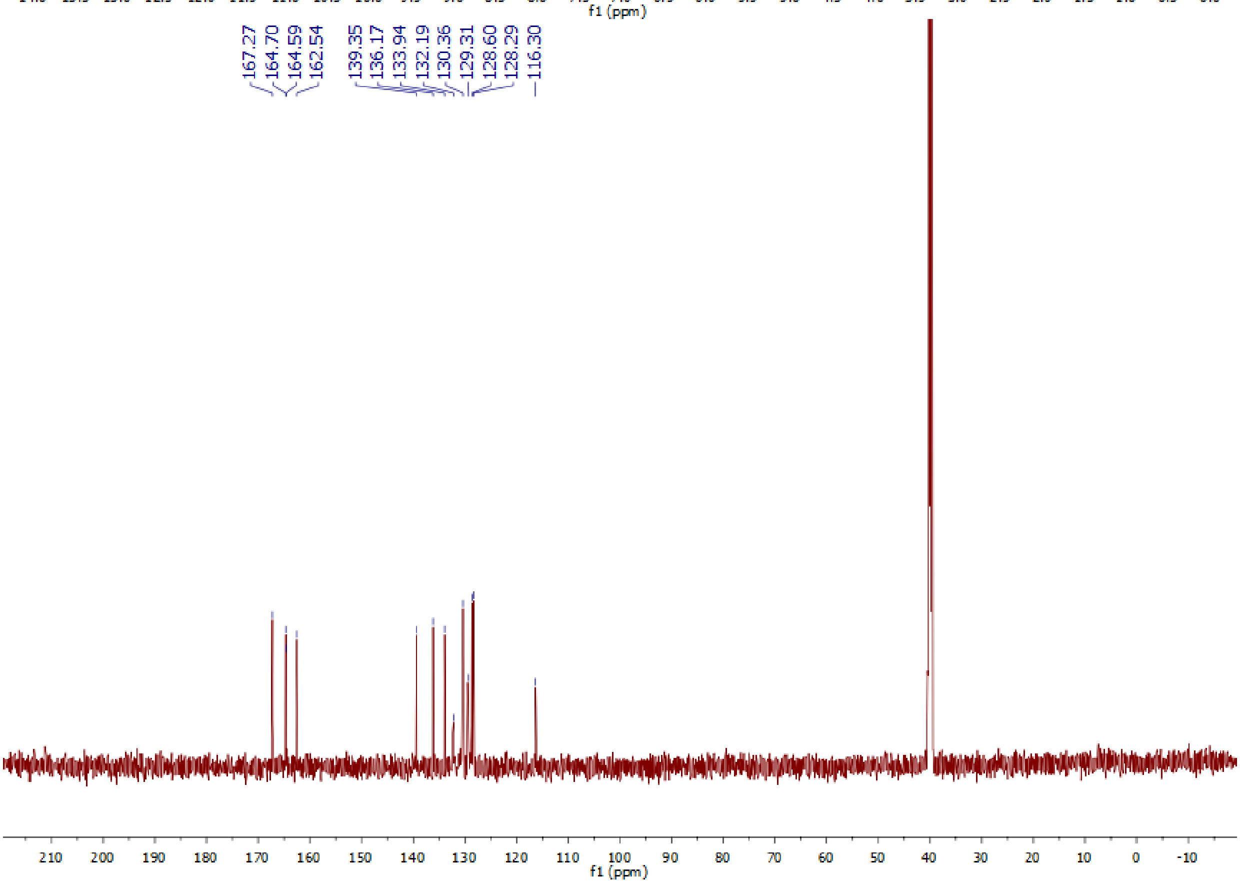

167.27
164.70
164.59
162.54
139.35
136.17
133.94
132.19
130.36
129.31
128.60
128.29
116.30
210 200 190 180 170 160 150 140 130 120 110 100 90 80 70 60 50 40 30 20 10 0 -10
f1 (ppm)

5, FM012

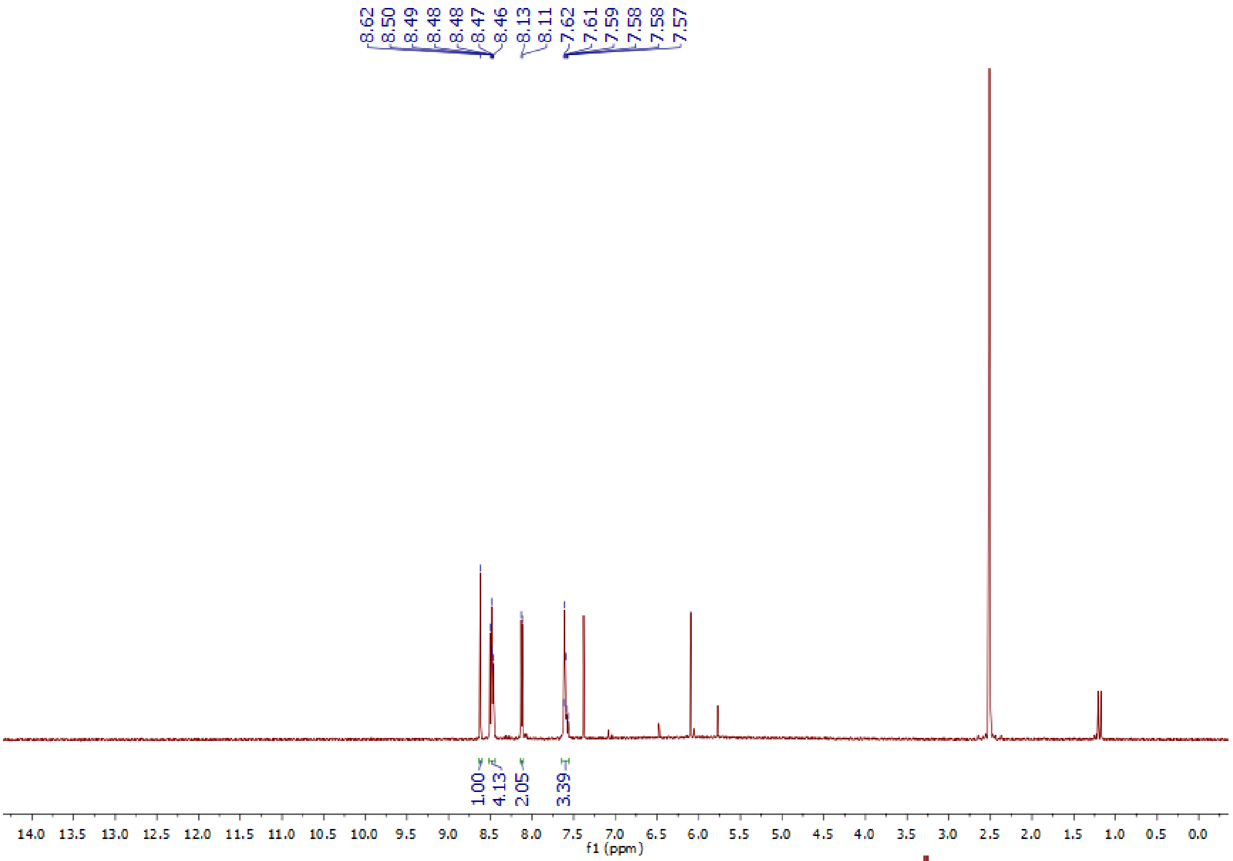
8.62
8.50
8.49
8.48
8.48
8.47
8.46
8.13
8.11
7.62
7.61
7.59
7.58
7.58
7.57
1.00
4.13
2.05
3.39
14.0 13.5 13.0 12.5 12.0 11.5 11.0 10.5 10.0 9.5 9.0 8.5 8.0 7.5 7.0 6.5 6.0 5.5 5.0 4.5 4.0 3.5 3.0 2.5 2.0 1.5 1.0 0.5 0.0
f1 (ppm)

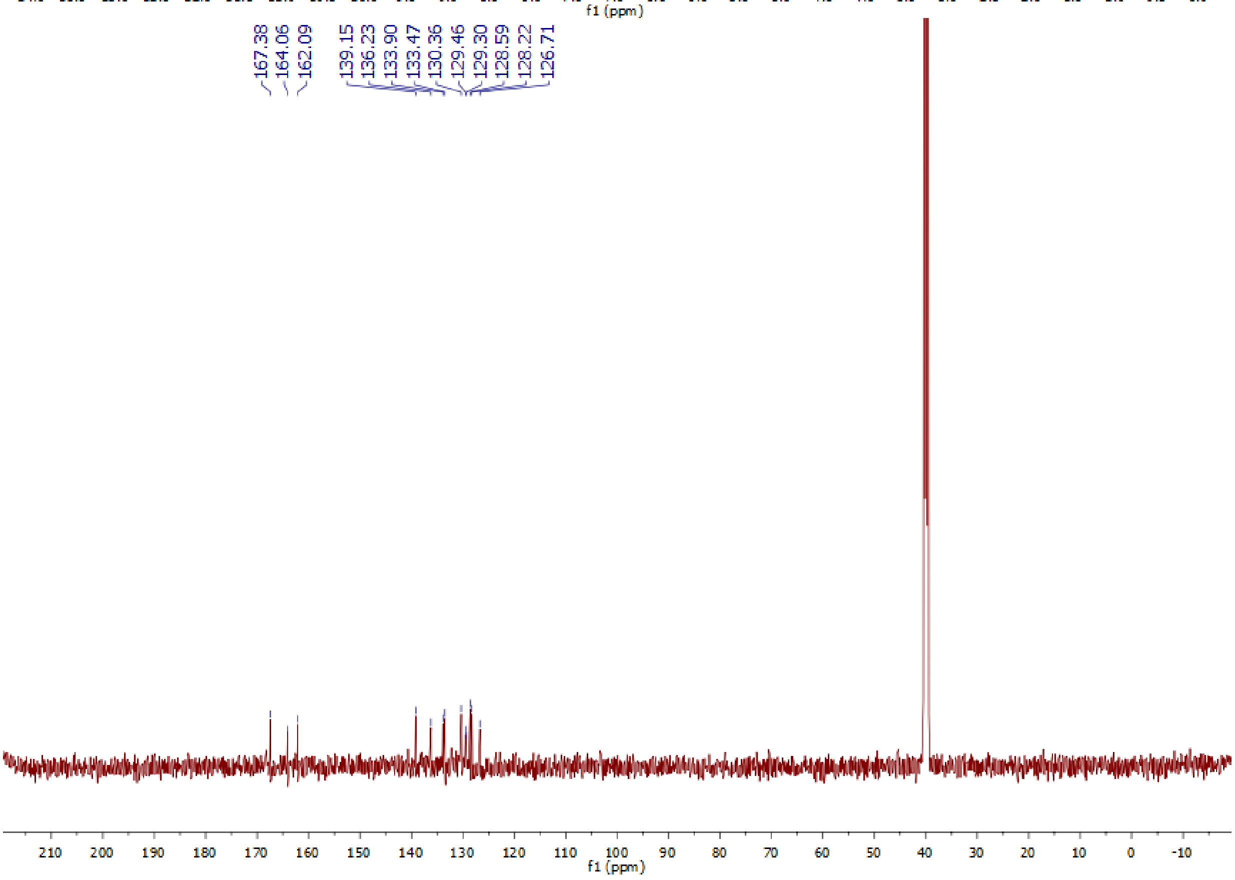
167.38
164.06
162.09
139.15
136.23
133.90
133.47
130.36
129.46
129.30
128.59
128.22
126.71
210 200 190 180 170 160 150 140 130 120 110 100 90 80 70 60 50 40 30 20 10 0 -10
f1 (ppm)

6a, FM015

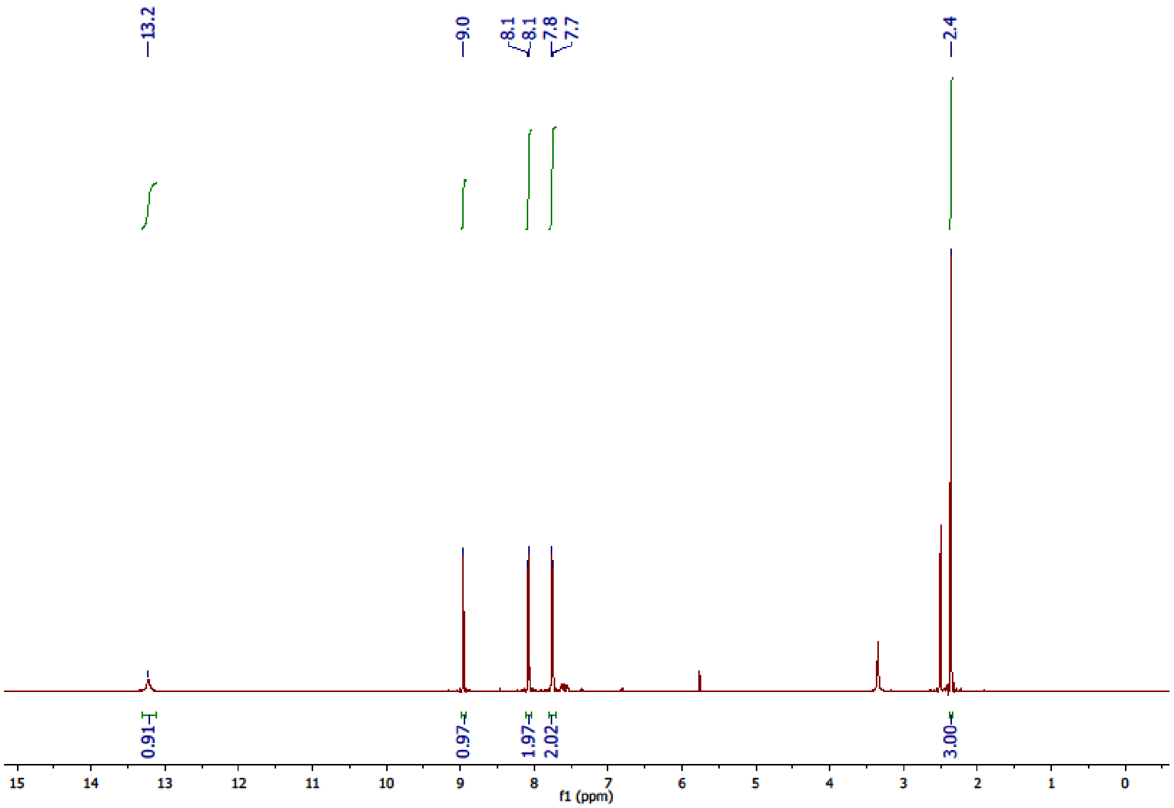

13.2
9.0
8.1
8.1
7.8
7.7
2.4
0.91
0.97
1.97
2.02
3.00
15
14
13
12
11
10
9
8
7
6
5
4
3
2
1
0
f1 (ppm)

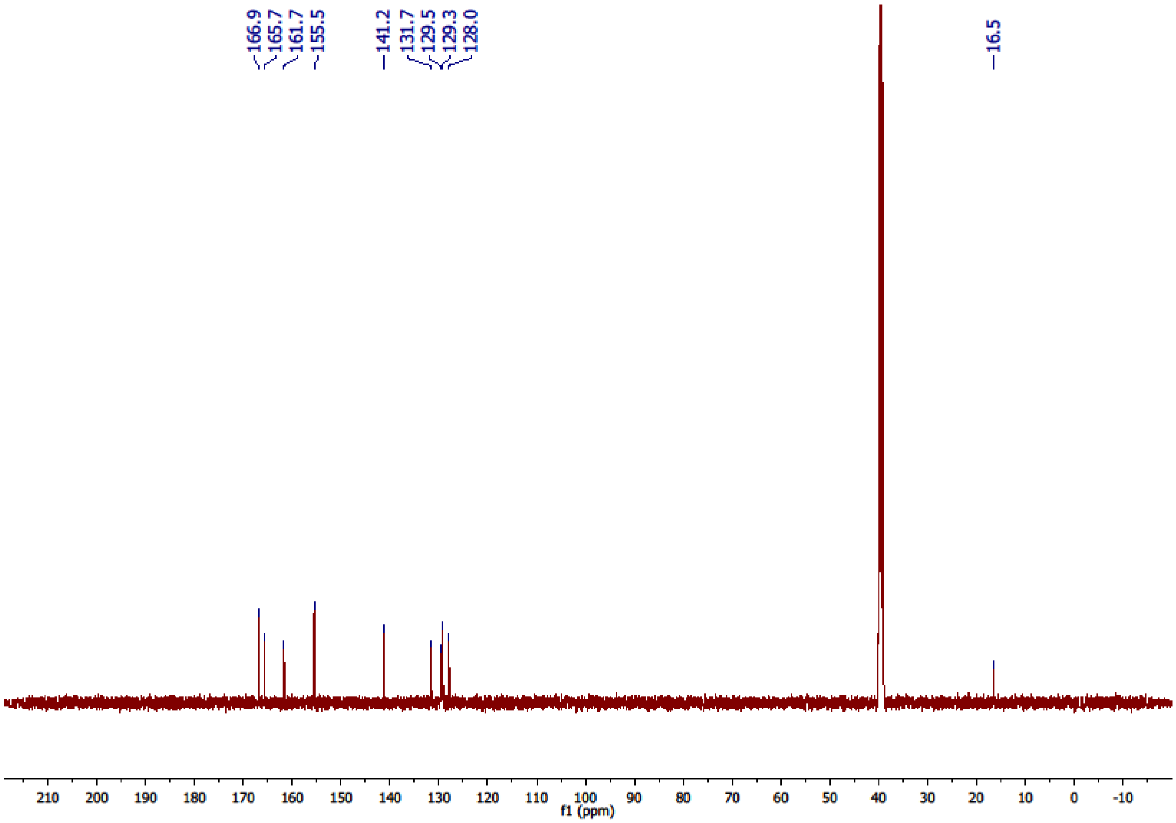

166.9
165.7
161.7
155.5
141.2
131.7
129.5
129.3
128.0
16.5
210
200
190
180
170
160
150
140
130
120
110
100
90
80
70
60
50
40
30
20
10
0
-10
f1 (ppm)

6, FM016

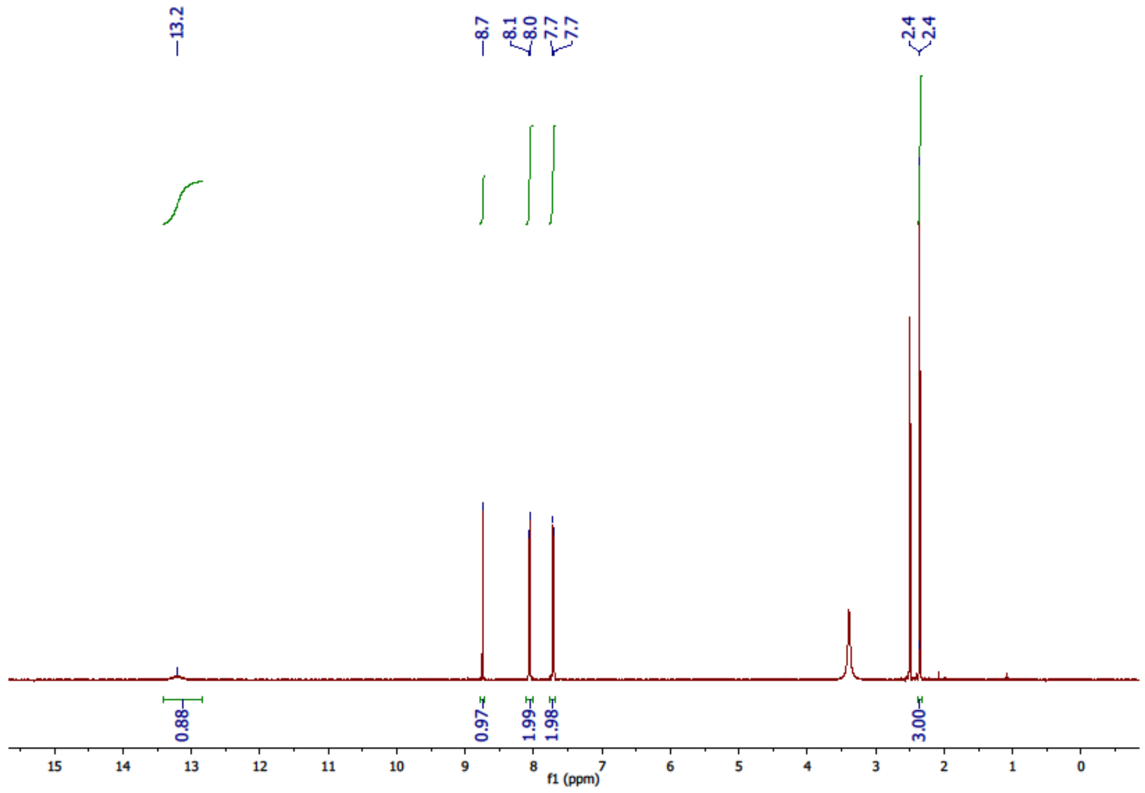

13.2
8.7
8.1
8.0
7.7
7.7
2.4
2.4
0.88
0.97
1.99
1.98
3.00
15
14
13
12
11
10
9
8
7
6
5
4
3
2
1
0
f1 (ppm)

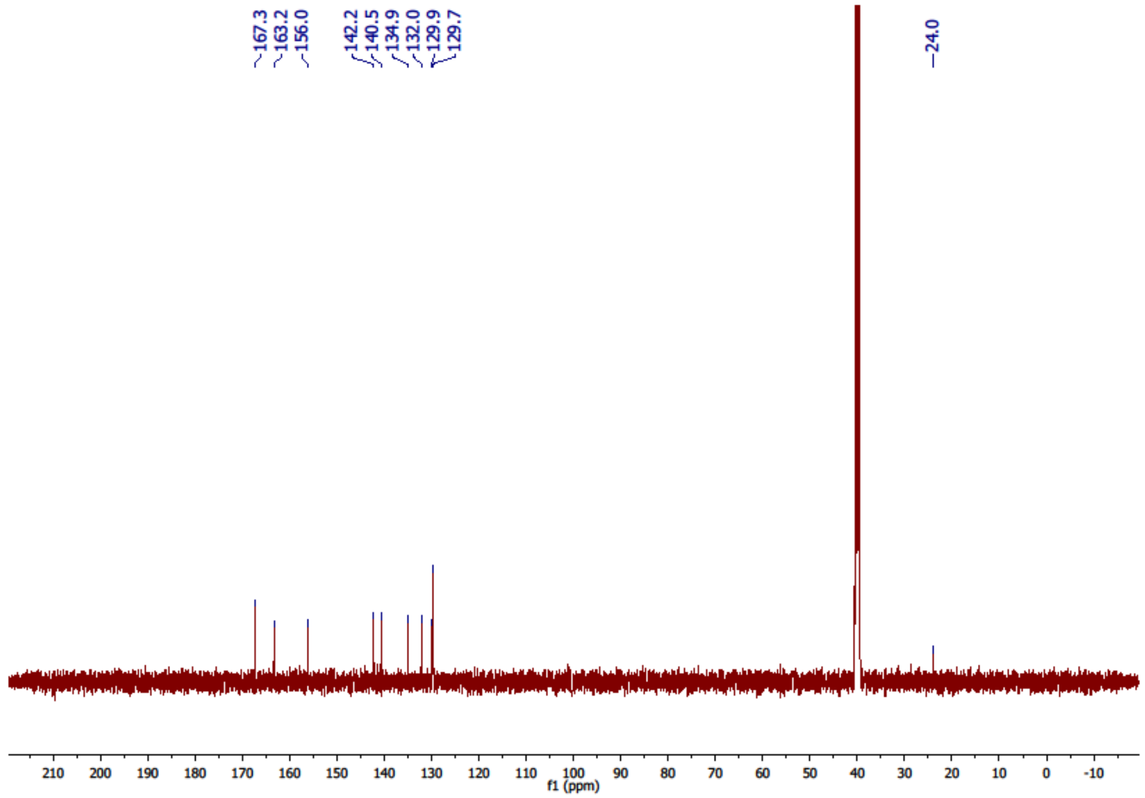

167.3
163.2
156.0
142.2
140.5
134.9
132.0
129.9
129.7
24.0
210
200
190
180
170
160
150
140
130
120
110
100
90
80
70
60
50
40
30
20
10
0
-10
f1 (ppm)

7a, FM007

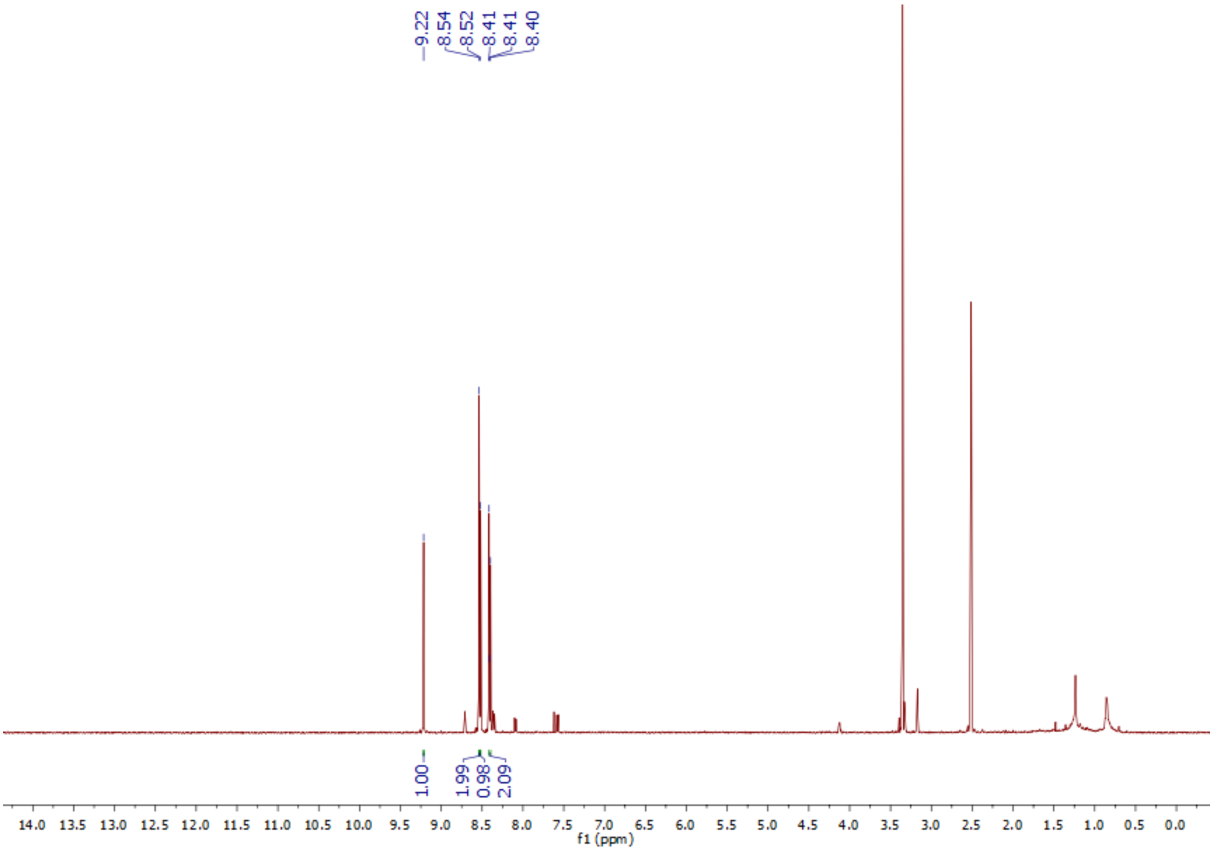
9.22
8.54
8.52
8.41
8.41
8.40
1.00
1.99
0.98
2.09
14.0 13.5 13.0 12.5 12.0 11.5 11.0 10.5 10.0 9.5 9.0 8.5 8.0 7.5 7.0 6.5 6.0 5.5 5.0 4.5 4.0 3.5 3.0 2.5 2.0 1.5 1.0 0.5 0.0
f1 (ppm)

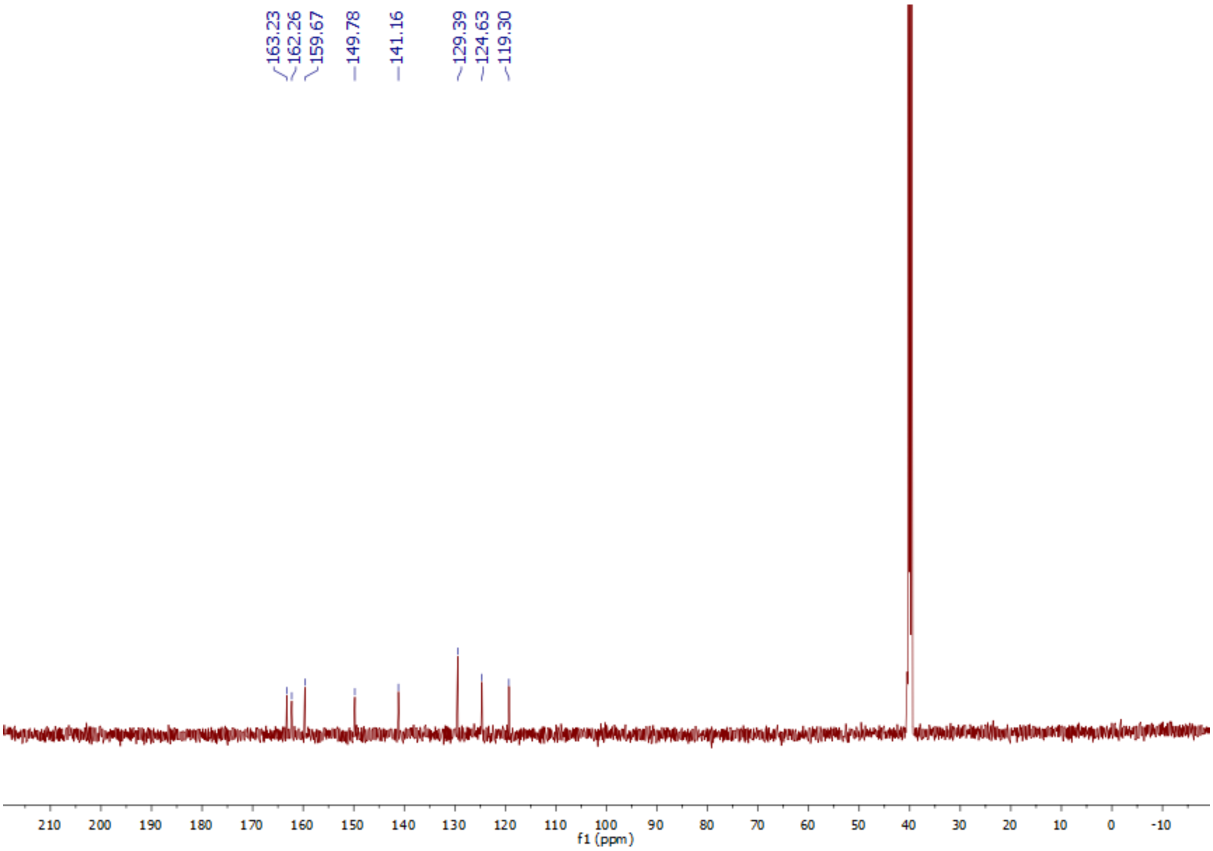
163.23
162.26
159.67
149.78
141.16
129.39
124.63
119.30
210 200 190 180 170 160 150 140 130 120 110 100 90 80 70 60 50 40 30 20 10 0 -10
f1 (ppm)

7, FM008

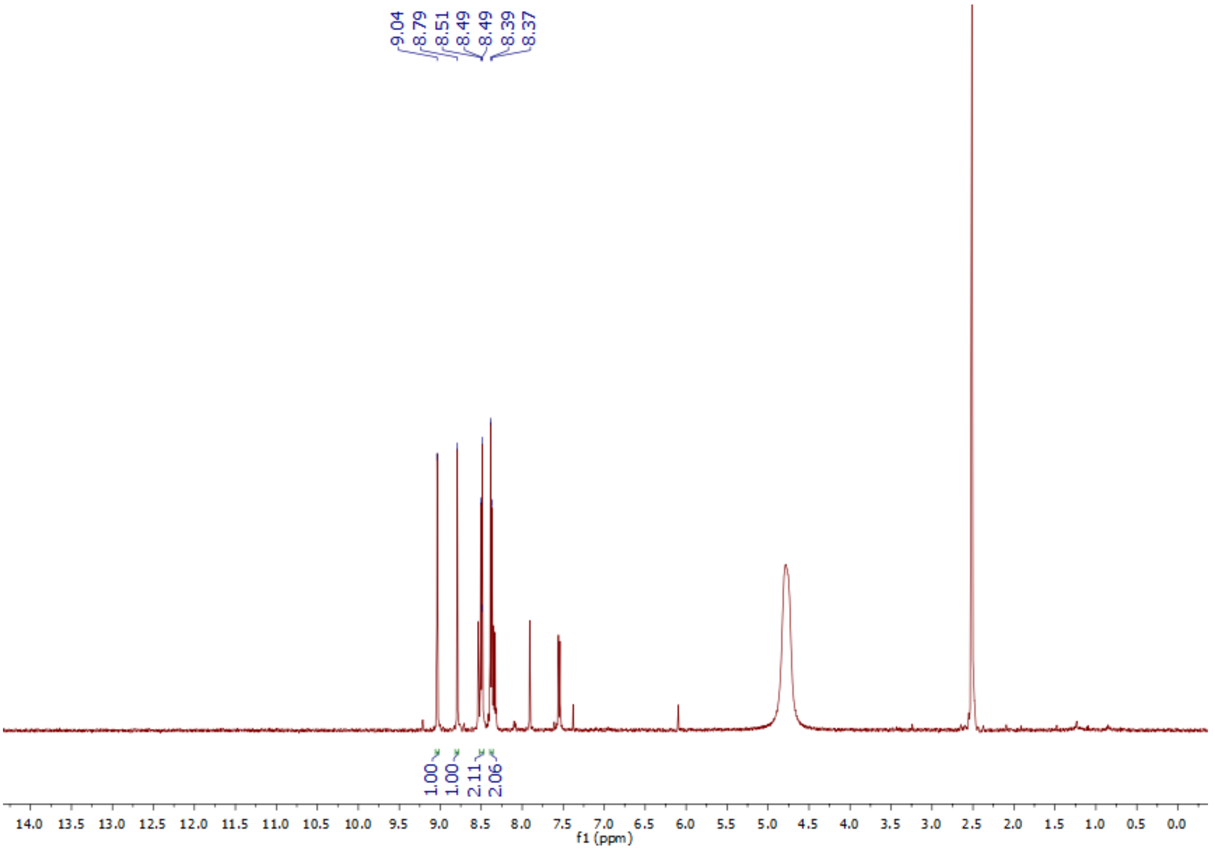

9.04
8.79
8.51
8.49
8.49
8.39
8.37
1.00
1.00
2.11
2.06
14.0 13.5 13.0 12.5 12.0 11.5 11.0 10.5 10.0 9.5 9.0 8.5 8.0 7.5 7.0 6.5 6.0 5.5 5.0 4.5 4.0 3.5 3.0 2.5 2.0 1.5 1.0 0.5 0.0
f1 (ppm)

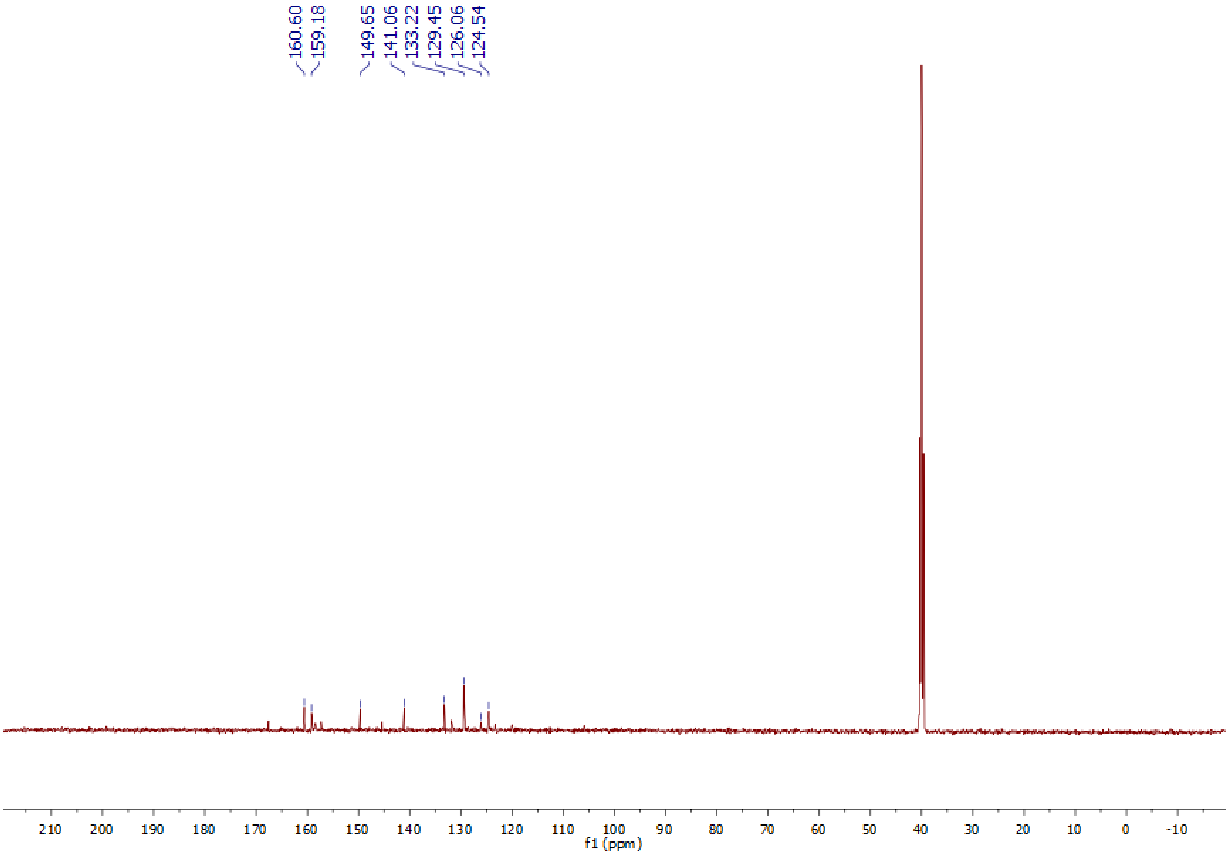

160.60
159.18
149.65
141.06
133.22
129.45
126.06
124.54
210 200 190 180 170 160 150 140 130 120 110 100 90 80 70 60 50 40 30 20 10 0 -10
f1 (ppm)

11a, FM023

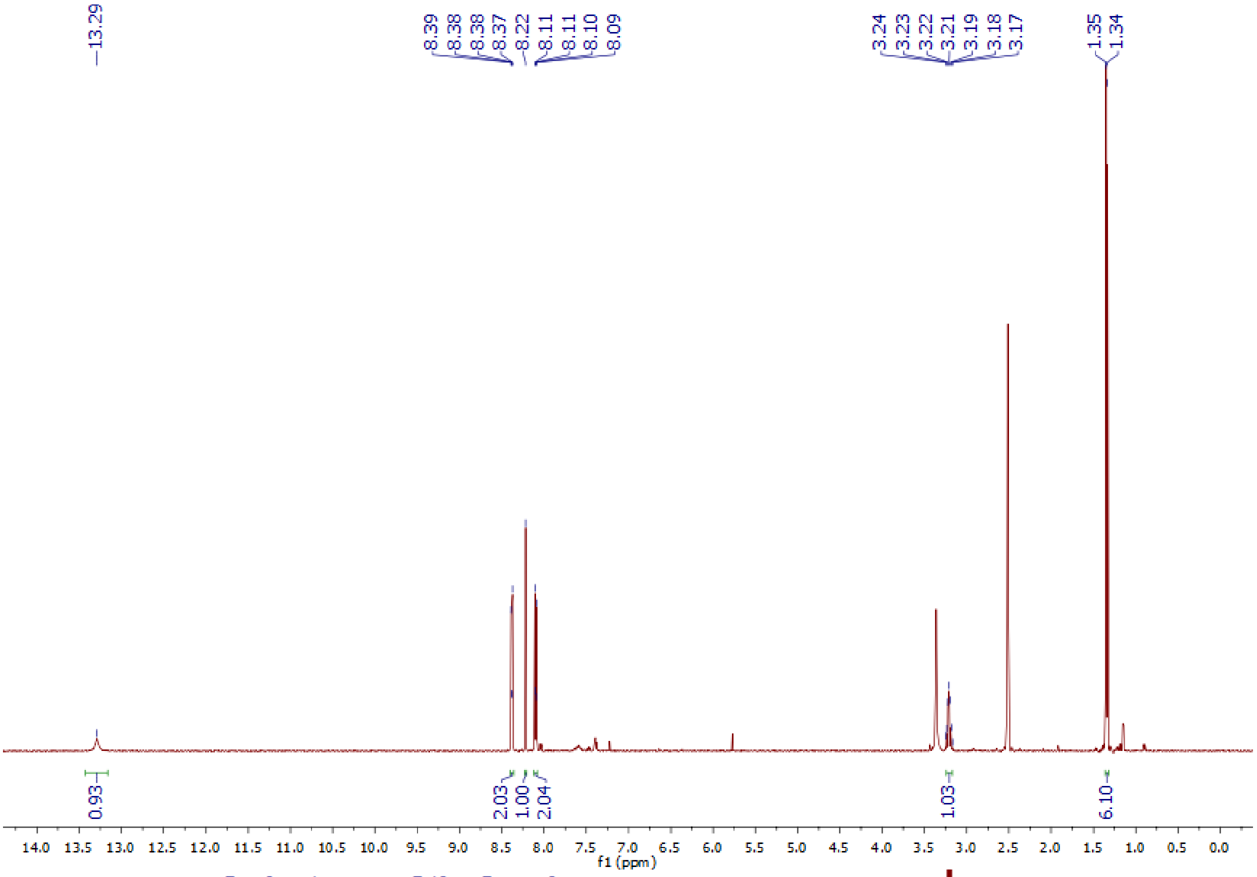

13.29
8.39
8.38
8.38
8.37
8.22
8.11
8.11
8.10
8.09
3.24
3.23
3.22
3.21
3.19
3.18
3.17
1.35
1.34
0.93
2.03
1.00
2.04
1.03
6.10
14.0 13.5 13.0 12.5 12.0 11.5 11.0 10.5 10.0 9.5 9.0 8.5 8.0 7.5 7.0 6.5 6.0 5.5 5.0 4.5 4.0 3.5 3.0 2.5 2.0 1.5 1.0 0.5 0.0
f1 (ppm)

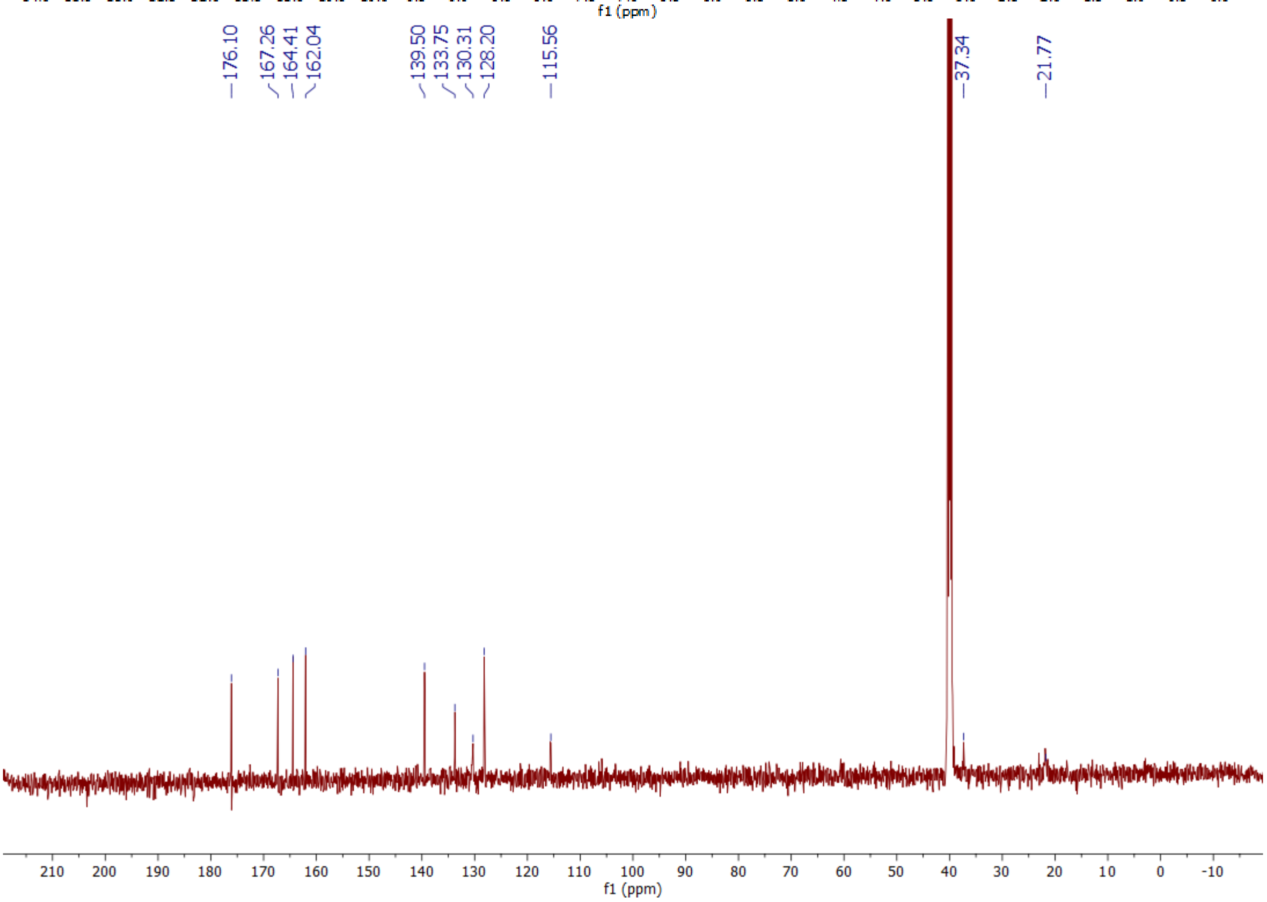

176.10
167.26
164.41
162.04
139.50
133.75
130.31
128.20
115.56
37.34
21.77
210 200 190 180 170 160 150 140 130 120 110 100 90 80 70 60 50 40 30 20 10 0 -10
f1 (ppm)

11, FM024

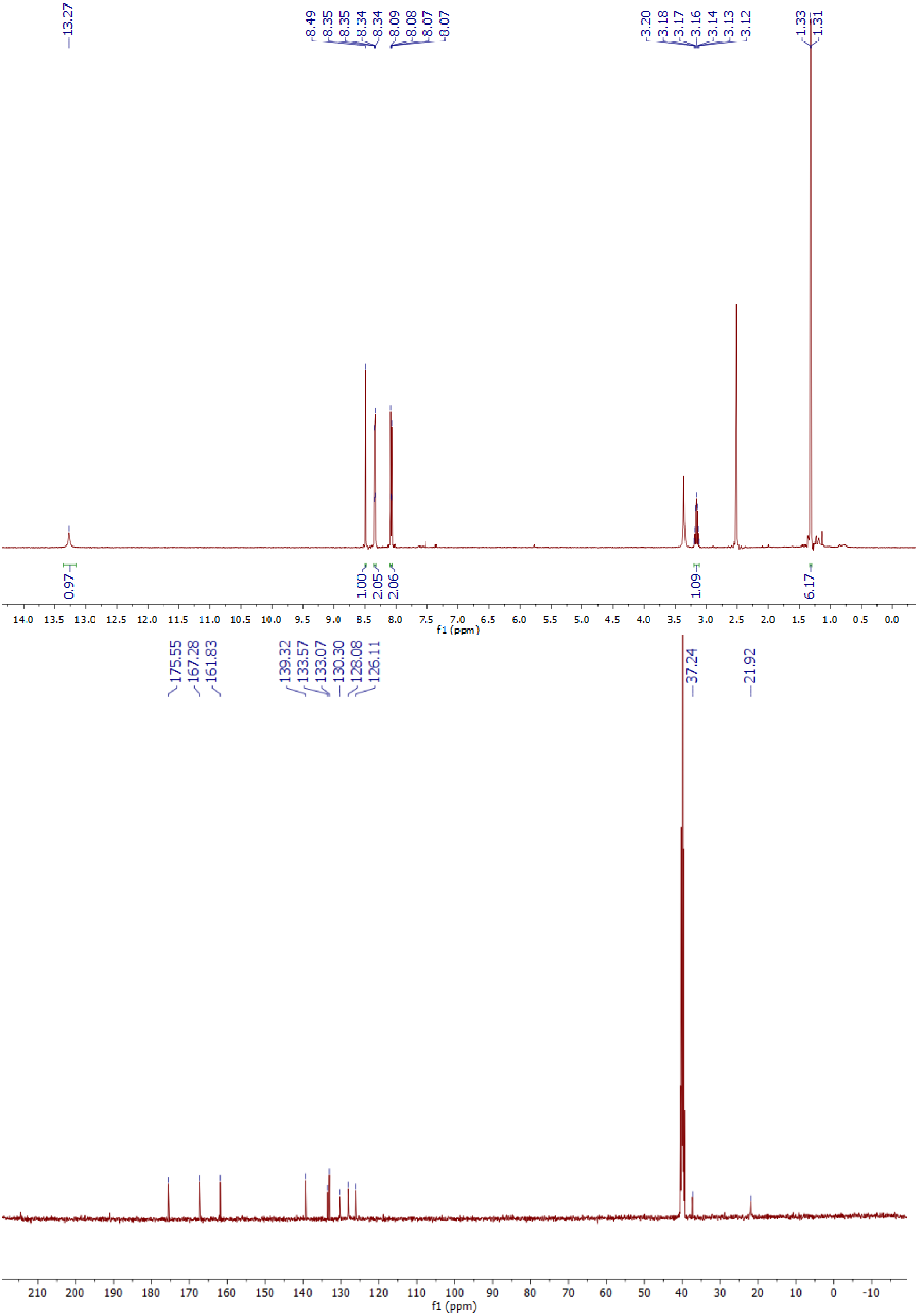

13.27
8.49
8.35
8.35
8.34
8.34
8.09
8.08
8.07
8.07
3.20
3.18
3.17
3.16
3.14
3.13
3.12
1.33
1.31
0.97
1.00
2.05
2.06
1.09
6.17
f1 (ppm)
175.55
167.28
161.83
139.32
133.57
133.07
130.30
128.08
126.11
37.24
21.92
f1 (ppm)

8a, ZP308

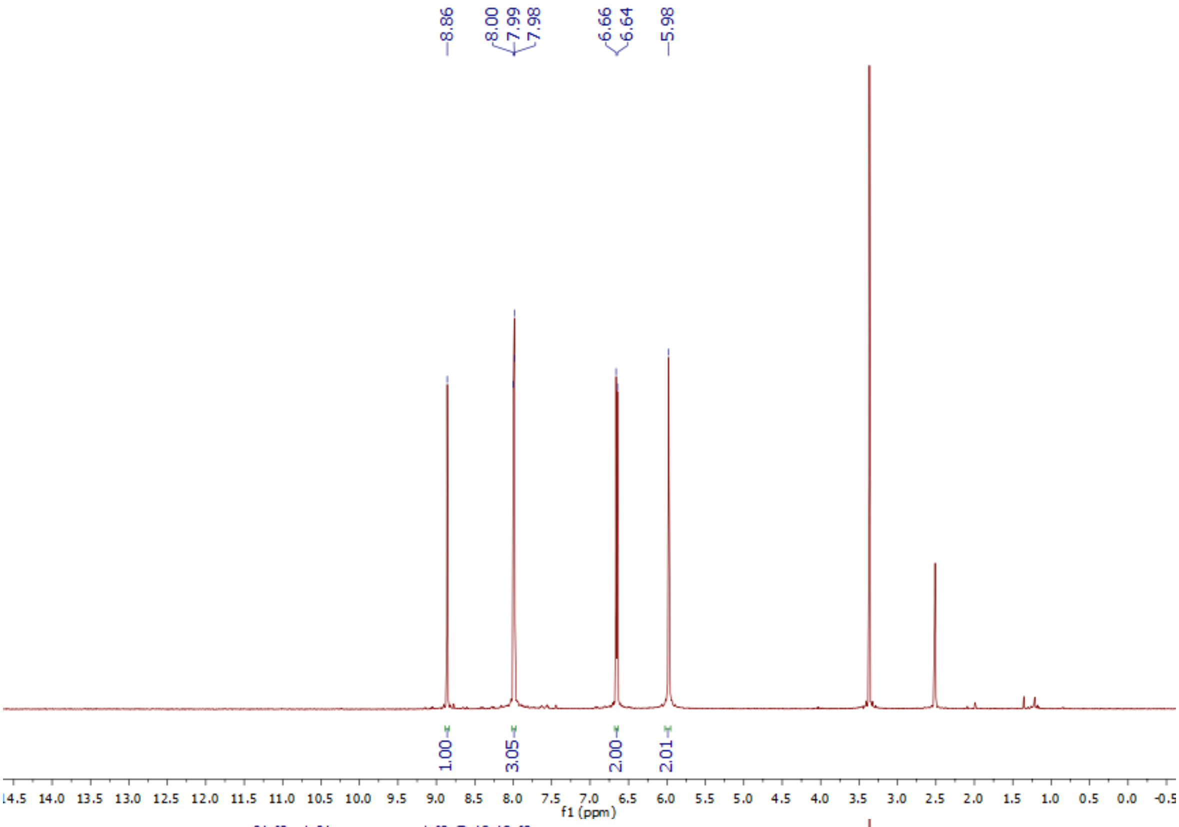

8.86
8.00
7.99
7.98
6.66
6.64
5.98
1.00
3.05
2.00
2.01
14.5 14.0 13.5 13.0 12.5 12.0 11.5 11.0 10.5 10.0 9.5 9.0 8.5 8.0 7.5 7.0 6.5 6.0 5.5 5.0 4.5 4.0 3.5 3.0 2.5 2.0 1.5 1.0 0.5 0.0 -0.5
f1 (ppm)

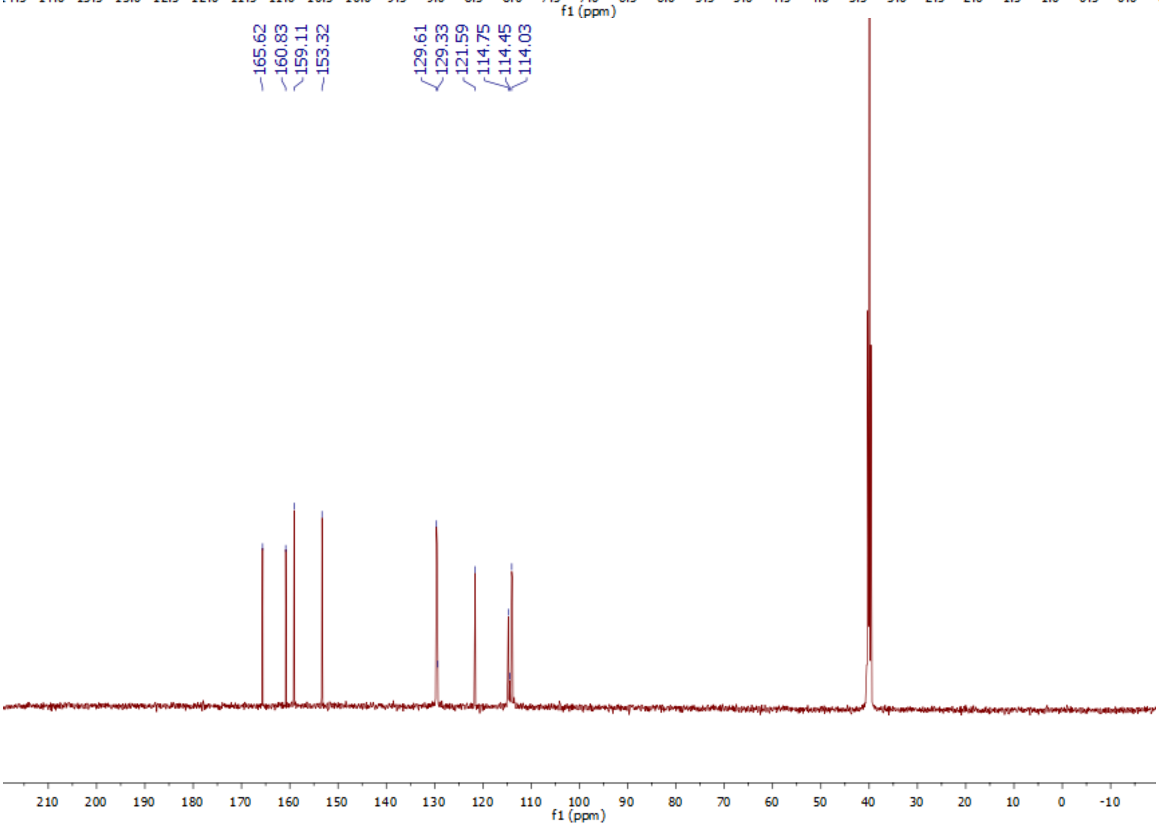

165.62
160.83
159.11
153.32
129.61
129.33
121.59
114.75
114.45
114.03
210 200 190 180 170 160 150 140 130 120 110 100 90 80 70 60 50 40 30 20 10 0 -10
f1 (ppm)

13, ZP254

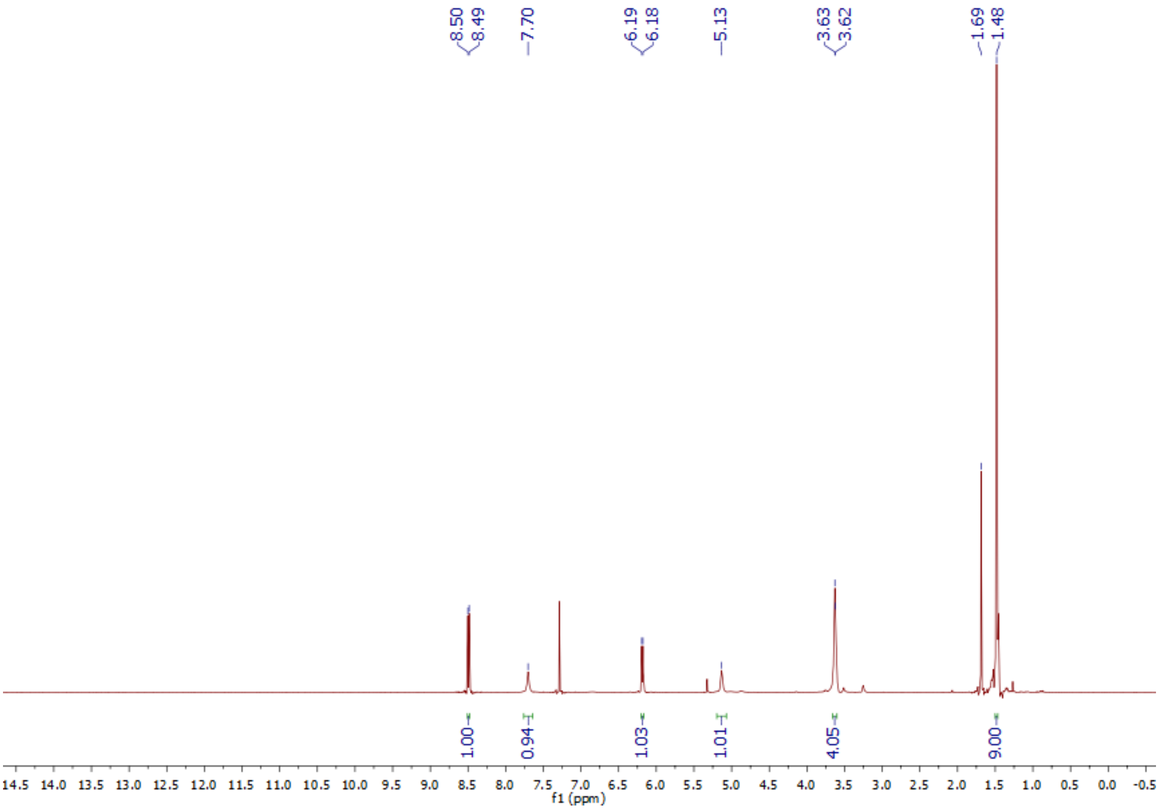
8.50
8.49
7.70
6.19
6.18
5.13
3.63
3.62
1.69
1.48
1.00
0.94
1.03
1.01
4.05
9.00
14.5 14.0 13.5 13.0 12.5 12.0 11.5 11.0 10.5 10.0 9.5 9.0 8.5 8.0 7.5 7.0 6.5 6.0 5.5 5.0 4.5 4.0 3.5 3.0 2.5 2.0 1.5 1.0 0.5 0.0 -0.5
f1 (ppm)

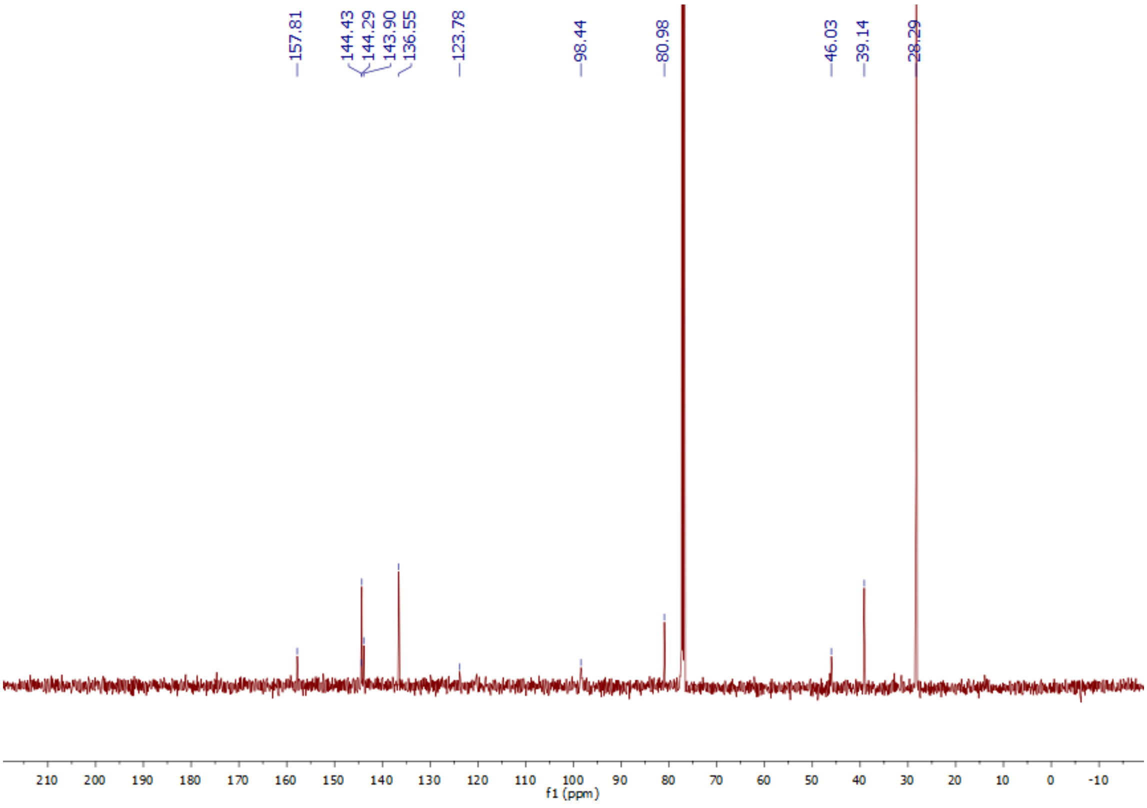
157.81
144.43
144.29
143.90
136.55
123.78
98.44
80.98
46.03
39.14
28.29
210 200 190 180 170 160 150 140 130 120 110 100 90 80 70 60 50 40 30 20 10 0 -10
f1 (ppm)

14, ZP306

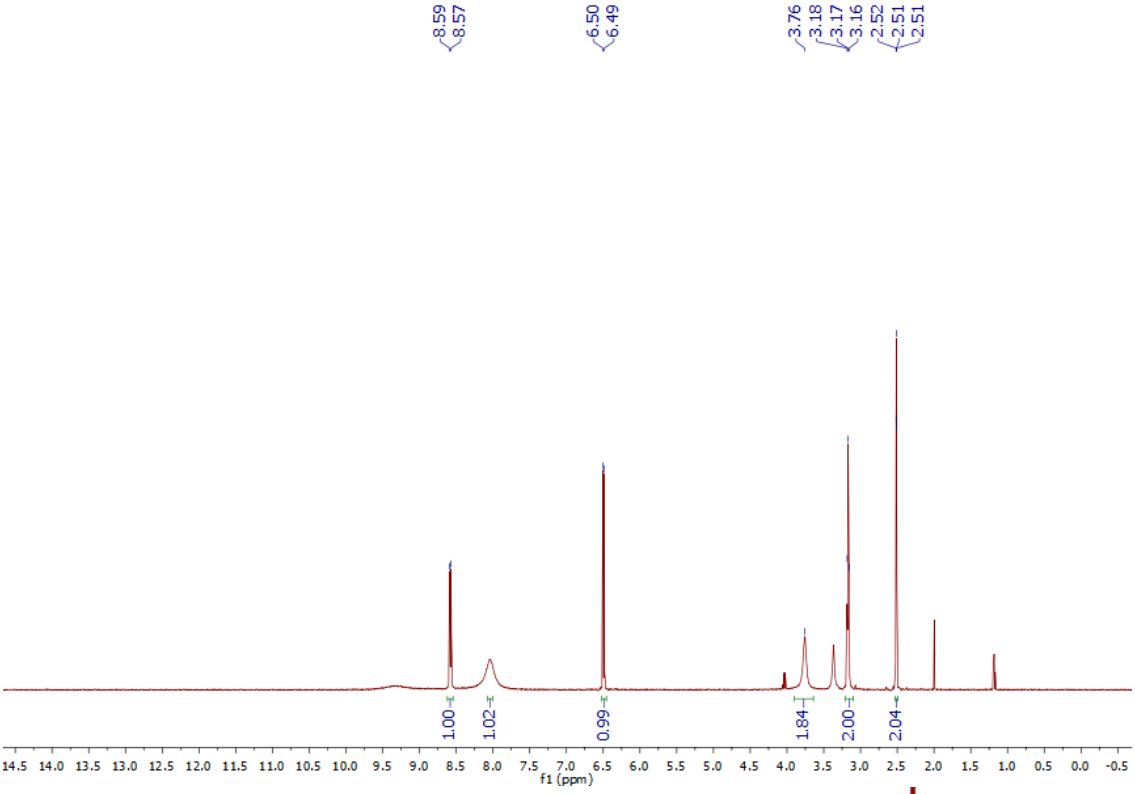
8.59
8.57
6.50
6.49
3.76
3.18
3.17
3.16
2.52
2.51
2.51
1.00
1.02
0.99
1.84
2.00
2.04
f1 (ppm)

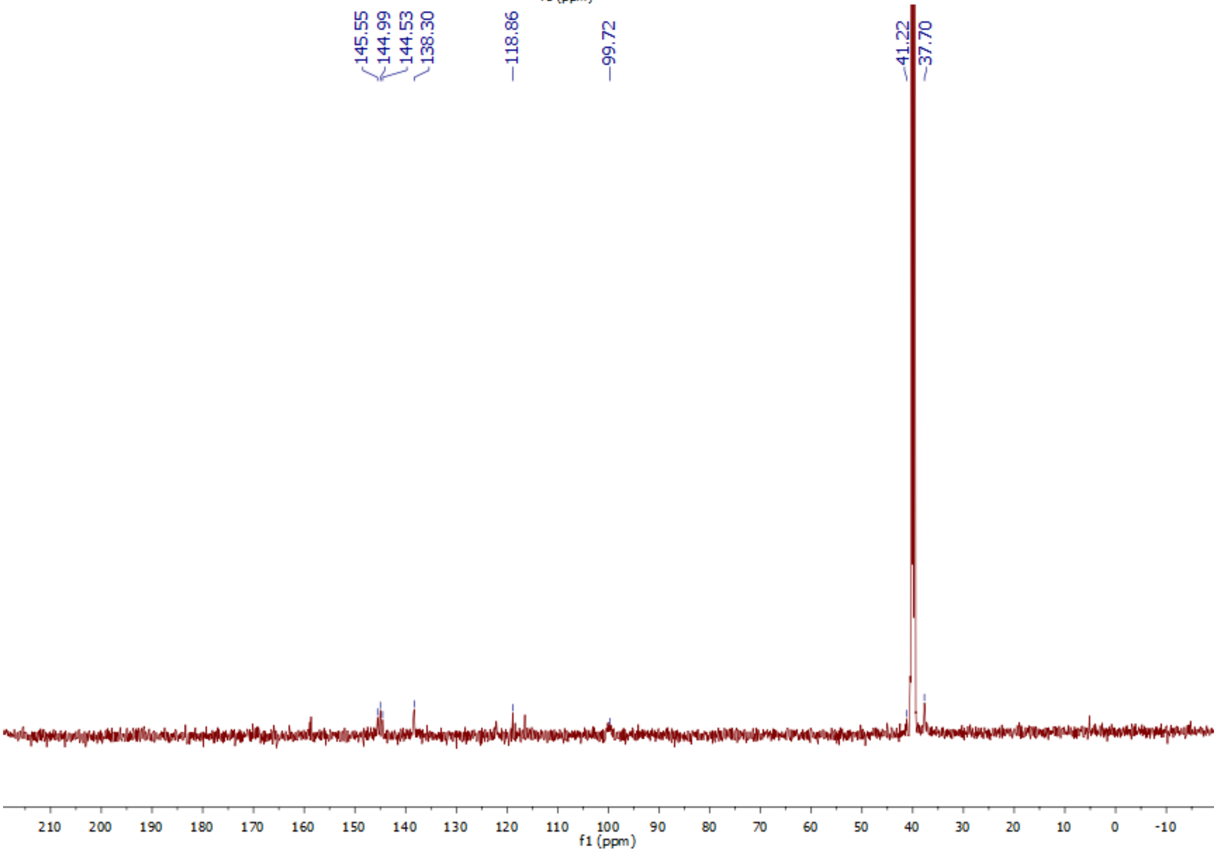
145.55
144.99
144.53
138.30
118.86
99.72
41.22
37.70
f1 (ppm)

15, ZP330

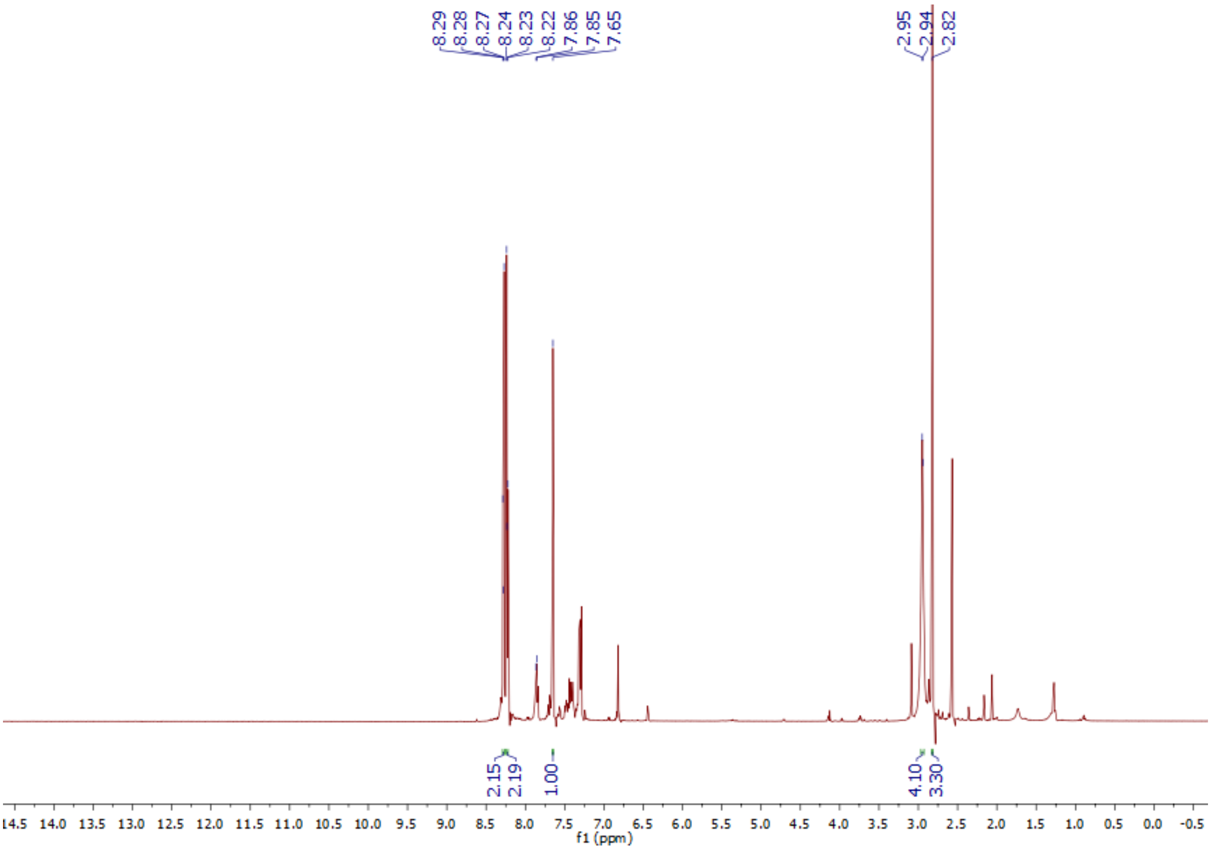
8.29
8.28
8.27
8.24
8.23
8.22
7.86
7.85
7.65
2.95
2.94
2.82
2.15
2.19
1.00
4.10
3.30
14.5 14.0 13.5 13.0 12.5 12.0 11.5 11.0 10.5 10.0 9.5 9.0 8.5 8.0 7.5 7.0 6.5 6.0 5.5 5.0 4.5 4.0 3.5 3.0 2.5 2.0 1.5 1.0 0.5 0.0 -0.5
f1 (ppm)

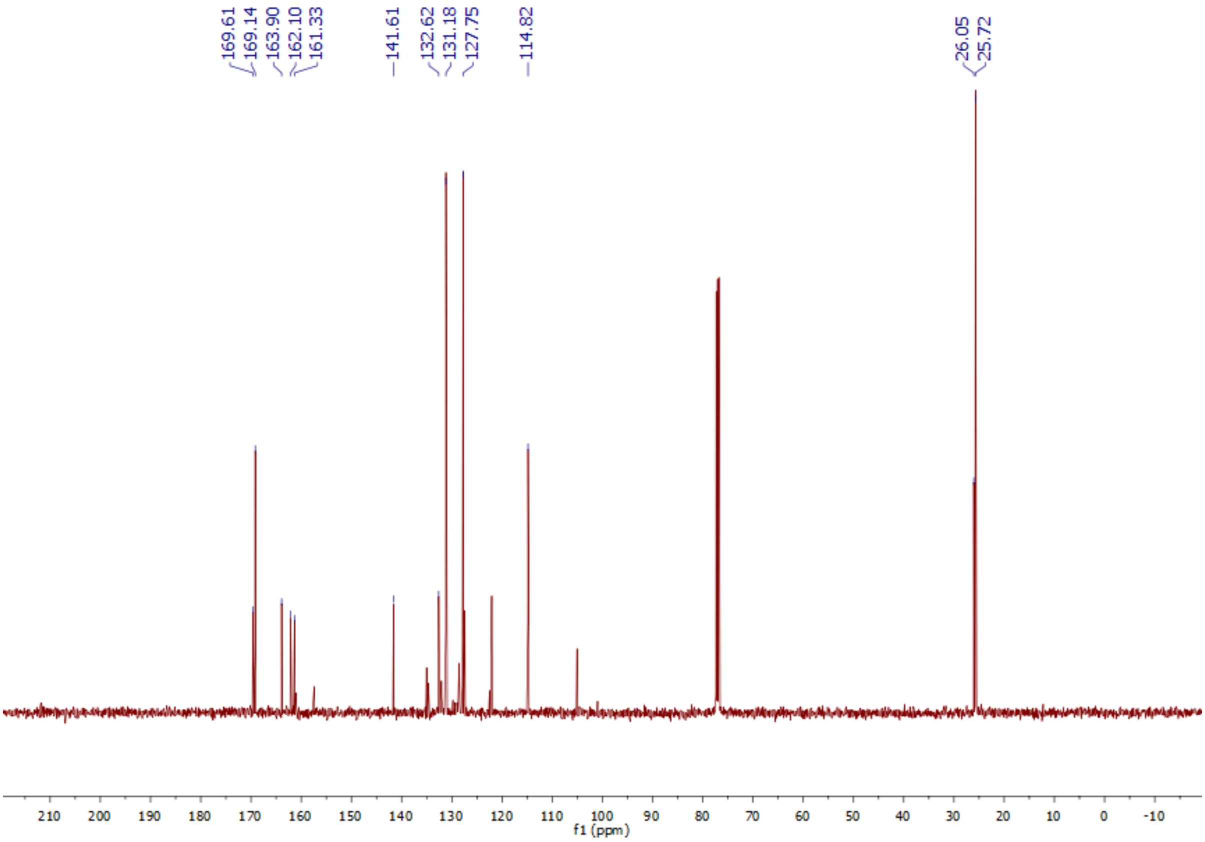
169.61
169.14
163.90
162.10
161.33
141.61
132.62
131.18
127.75
114.82
26.05
25.72
210 200 190 180 170 160 150 140 130 120 110 100 90 80 70 60 50 40 30 20 10 0 -10
f1 (ppm)

8, ZP307

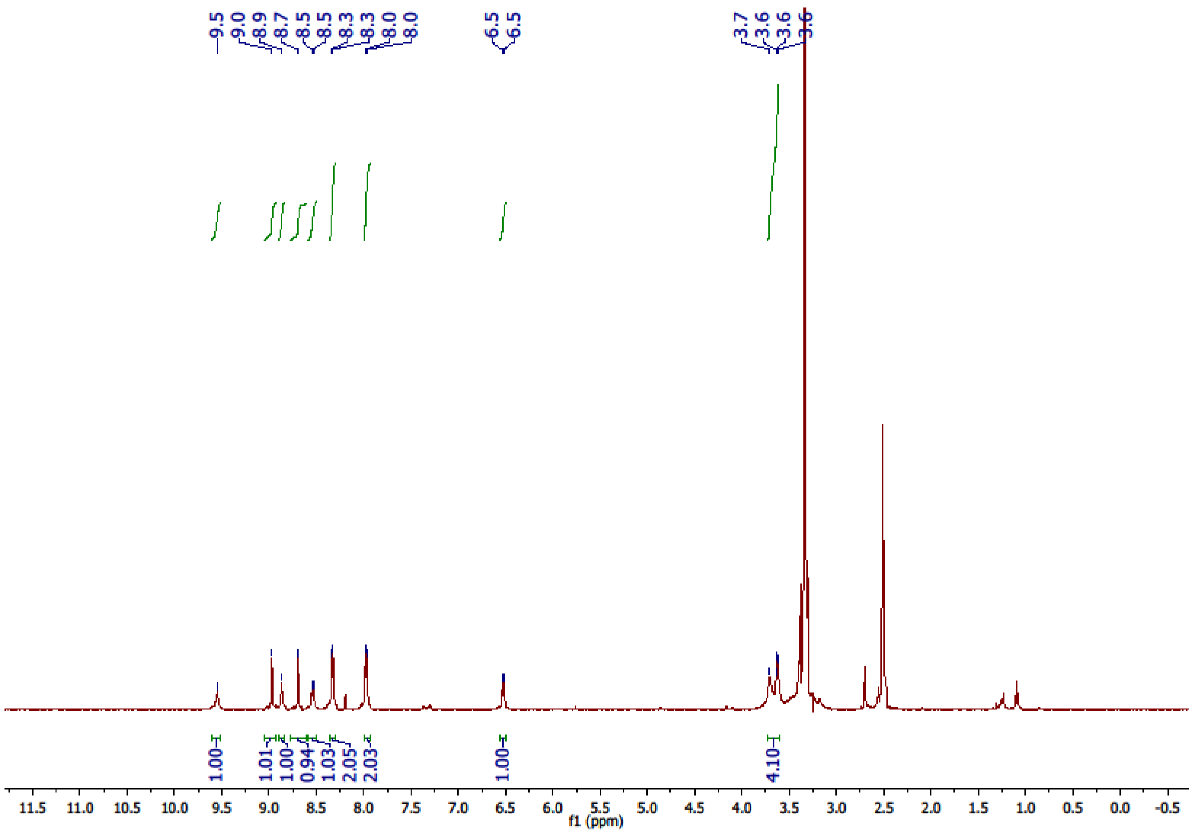
9.5
9.0
8.9
8.7
8.5
8.5
8.3
8.3
8.0
8.0
6.5
6.5
3.7
3.6
3.6
3.6
1.00
1.01
1.00
0.94
1.03
2.05
2.03
1.00
4.10
11.5
11.0
10.5
10.0
9.5
9.0
8.5
8.0
7.5
7.0
6.5
6.0
5.5
5.0
4.5
4.0
3.5
3.0
2.5
2.0
1.5
1.0
0.5
0.0
-0.5
f1 (ppm)

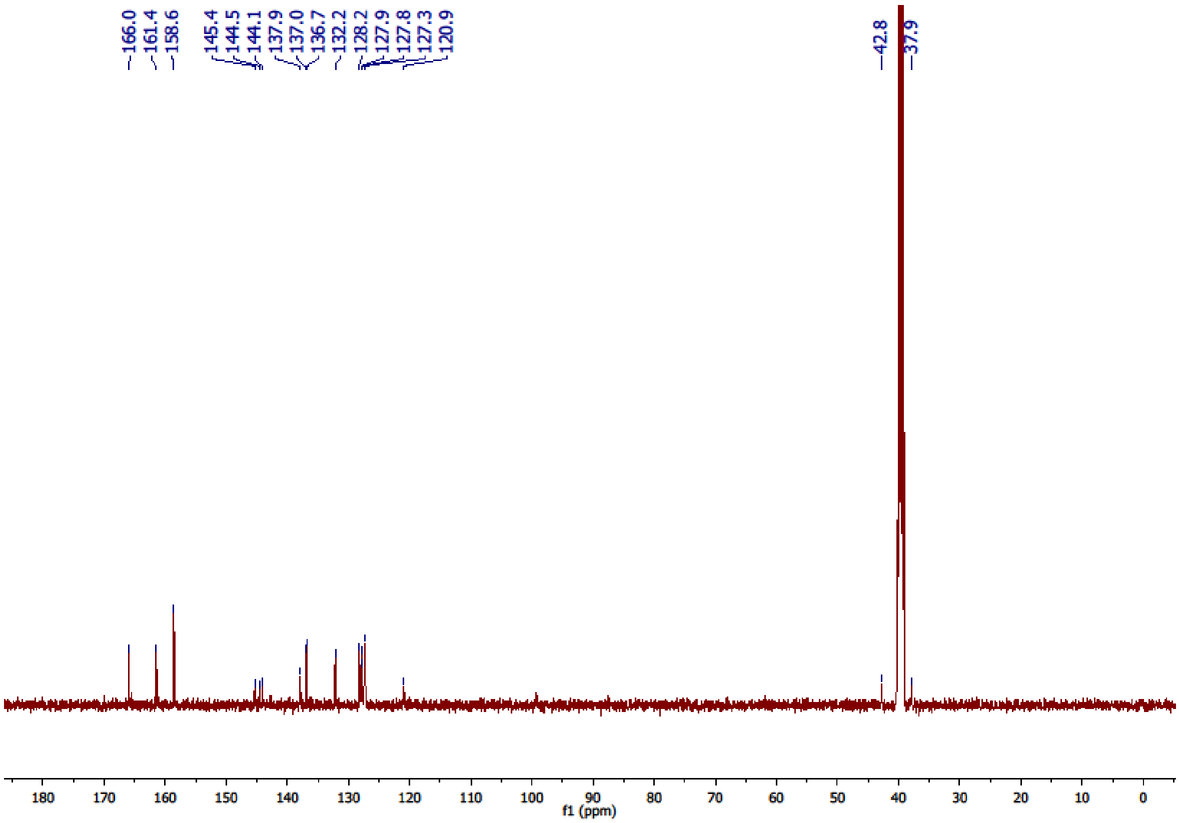
166.0
161.4
158.6
145.4
144.5
144.1
137.9
137.0
136.7
132.2
128.2
127.9
127.8
127.3
120.9
42.8
37.9
180
170
160
150
140
130
120
110
100
90
80
70
60
50
40
30
20
10
0
f1 (ppm)

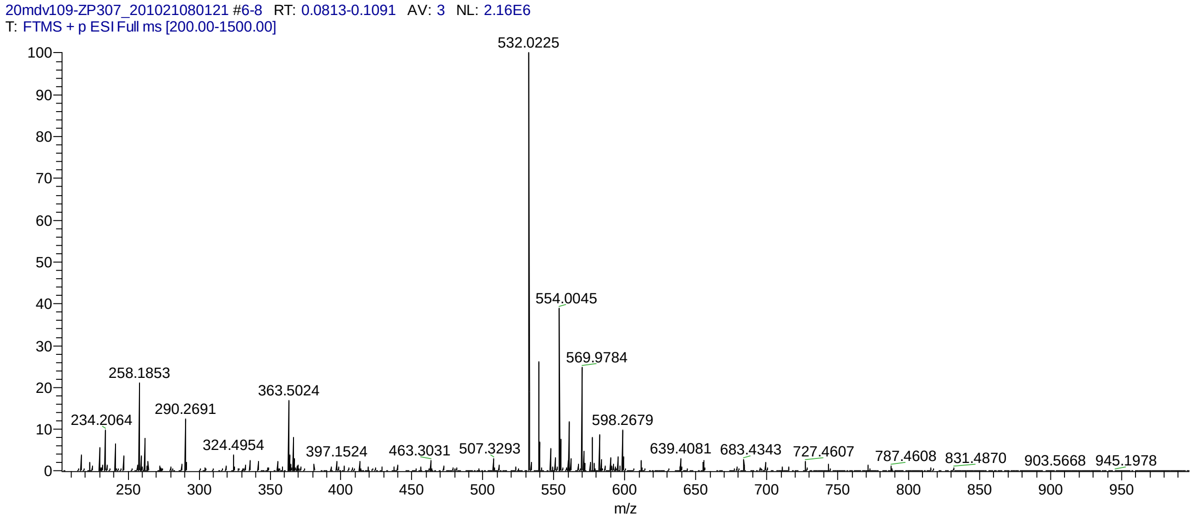
20mdv109-ZP307_201021080121 #6-8 RT: 0.0813-0.1091 AV: 3 NL: 2.16E6
T: FTMS + p ESI Full ms [200.00-1500.00]
532.0225
554.0045
569.9784
258.1853
363.5024
290.2691
234.2064
598.2679
324.4954
397.1524
463.3031
507.3293
639.4081
683.4343
727.4607
787.4608
831.4870
903.5668
945.1978
m/z

9, FM031

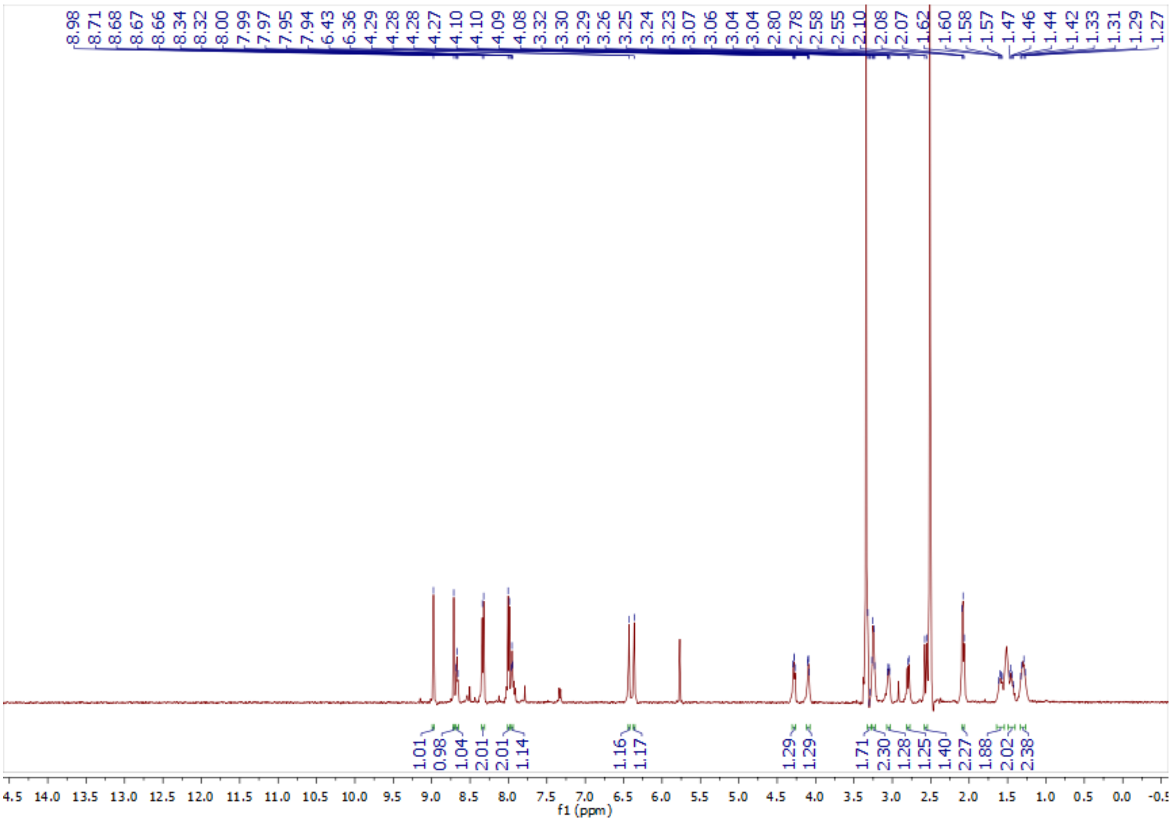
8.98 8.71 8.68 8.67 8.66 8.34 8.32 8.00 7.99 7.97 7.95 7.94 6.43 6.36 4.29 4.28 4.28 4.27 4.10 4.10 4.09 4.08 3.32 3.30 3.29 3.26 3.25 3.24 3.23 3.07 3.06 3.04 3.04 2.80 2.78 2.58 2.55 2.10 2.08 2.07 1.62 1.60 1.58 1.57 1.47 1.46 1.44 1.42 1.33 1.31 1.29 1.27
1.01 0.98 1.04 2.01 2.01 1.14 1.16 1.17 1.29 1.29 1.71 2.30 1.28 1.25 1.40 2.27 1.88 2.02 2.38
f1 (ppm)

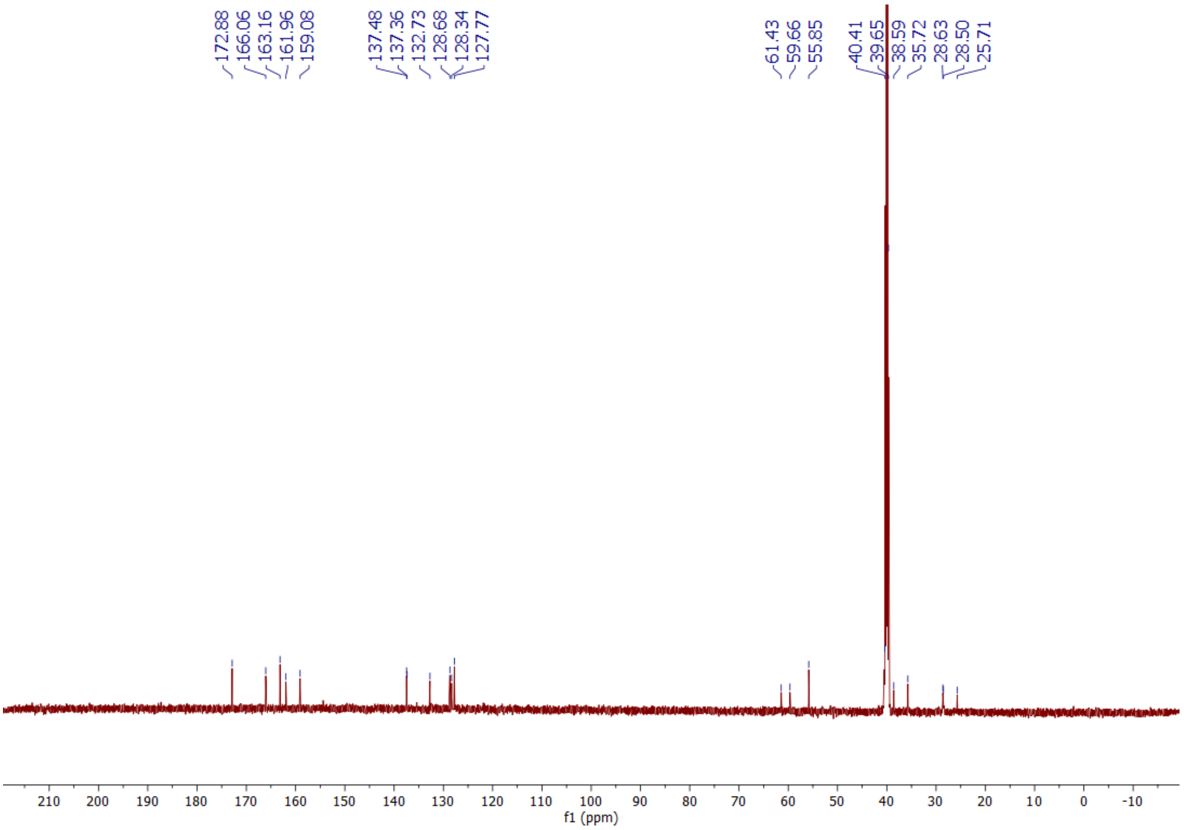
172.88 166.06 163.16 161.96 159.08 137.48 137.36 132.73 128.68 128.34 127.77 61.43 59.66 55.85 40.41 39.65 38.59 35.72 28.63 28.50 25.71
f1 (ppm)

21mdv011-15-fm031 #297 RT: 4.4780 AV: 1 NL: 6.15E5
T: FTMS + p ESI Full ms [200.00-1200.00]

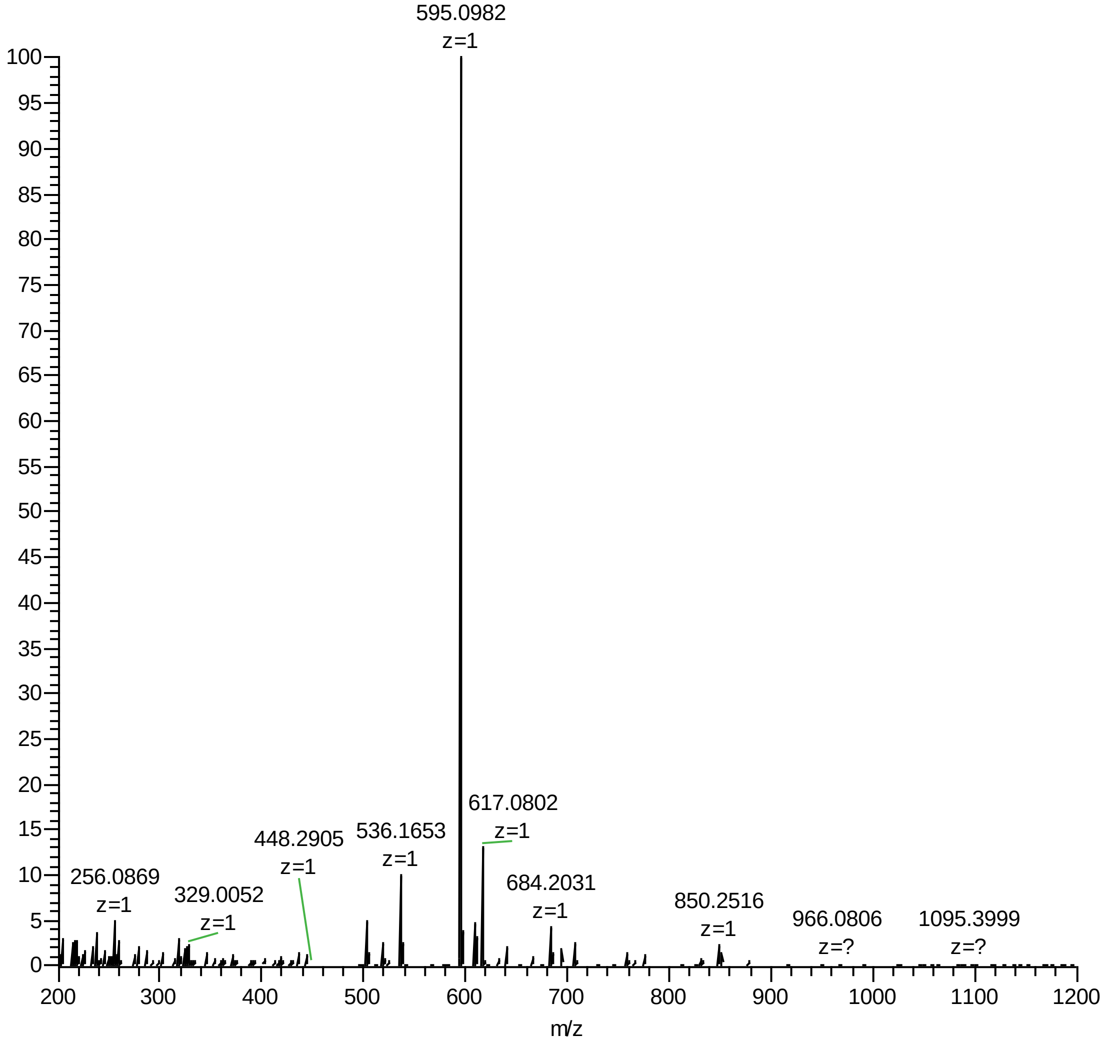

Supplement: Supplementary file 1 — Supporting Information [file CHEM-28-0-s001.pdf]
